# Substituent effects of fluorinated bambusurils on their anion transport†

**DOI:** 10.1039/d5ob00400d

**Published:** 2025-04-10

**Authors:** Matúš Chvojka, Vladimír Šindelář, Hennie Valkenier

**Affiliations:** a Department of Chemistry, Faculty of Science, Masaryk University Kamenice 5 625 00 Brno Czech Republic sindelar@chemi.muni.cz; b RECETOX, Faculty of Science, Masaryk University Kamenice 5 625 00 Brno Czech Republic; c Engineering of Molecular NanoSystems, École Polytechnique de Bruxelles, Université libre de Bruxelles Avenue F. Roosevelt 50 CP165/64 1050 Brussels Belgium hennie.valkenier@ulb.be

## Abstract

Anionophores are molecules that can transport ions across membranes. Several structural design criteria must be met for anionophores to be highly active. Fluorinated anionophores are usually more potent than their non-fluorinated analogues due to their higher lipophilicity and increased affinity for anions. Clear structure–activity relationships have been described for small and relatively simple anionophores. However, such studies are more challenging for large and macrocyclic anionophores, as their preparation is usually more complicated, limiting the number of compounds tested in anion transport studies. Here we present a series of twelve macrocyclic bambusuril anion transporters to investigate how variations in fluorinated substituents affect their transport properties. Measurements of Cl^−^/HCO_3_^−^ antiport activities in liposomes revealed links between parameters such as lipophilicity or substituent polarity and transport activity. For some bambusurils, an unusually large effect of the presence of cholesterol in the membrane on transport activity was found. Further studies showed that for very potent anion receptors, such as the bambusurils described here, the binding selectivity towards anions becomes more important than the absolute binding affinity to anions when considering anion exchange across the membrane.

## Abstract 2

Eight different types of fluorinated groups were used to functionalise benzylated bambusuril macrocycles, resulting in a library of eleven anion receptors with high chloride affinities and very different anion transport rates.

## Graphical abstract

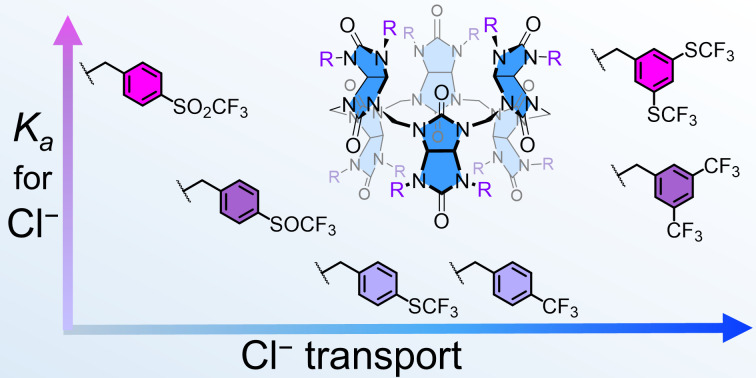

## Introduction

Ion transport is an important biological process regulating homeostasis, cell volume, membrane excitability, *etc*. As cell membranes are impermeable to charged and large hydrophilic species, these are transported across the membrane by specialised proteins. In healthy cells, an ensemble of different transmembrane proteins is present to maintain precise ion gradients across the membrane for normal biological activity. The absence and/or malfunction of any of the ion transporters has serious consequences. Some of the channelopathies associated with insufficient anion transport include cystic fibrosis, Dent's disease, Bartter syndrome, and Fahr's syndrome.^1–4^

One way of tackling these diseases is to use synthetic transporters, either as artificial channels or as mobile carriers (ionophores). These synthetic transporters have the advantage of being able to mediate ion transport independently of the existing natural proteins and therefore may be complementary to other types of treatment.^5^ In this context, transporters capable of transporting chloride and bicarbonate are relevant for cystic fibrosis (CF) treatment.^6,7^ Several studies showed promising results that synthetic anion transporters can indeed restore ion transport in CF cells.^8–12^

For the biological application of anionophores, several criteria must be met, such as high activity, low toxicity and selectivity for the desired anion to be transported. Highly active compounds are sought, as they would allow lower dosages with a reduced risk of undesirable side effects and lower costs for both production and patients. There is already a large pool of different anion receptors serving as anionophores.^13–17^ These have been constructed from moieties that interact with anions *via e.g.* hydrogen or halogen bond donor groups, which are attached to various scaffolds, including steroids,^18^ carbazoles,^19,20^ polyamines^21–23^ or macrocycles.^24–27^

In addition to selecting a scaffold and binding groups, the choice of substituents is crucial to obtain a highly active transporter. Substituents must be chosen with care to regulate parameters such as lipophilicity or anion binding strength. The impact of substituents on transport activities is well documented, particularly for relatively small and simple anionophores.^28–30^ Anionophores with fluorinated substituents usually exhibit high levels of activity, attributable to the strongly electron-withdrawing property of fluorine atoms. This results in the polarization of the anion-binding site, thereby enhancing anion binding. An increase in anion binding generally leads to an enhancement in transport rates up to a point where anion decomplexation might become the rate limiting process in the anion transport.^31–33^ Additionally, most fluorinated molecules possess a high lipophilicity, which further aids diffusion across the lipid bilayer and increases transport activity.^34,35^

Fluorinated substituents in anion transporters are usually limited to phenyl rings containing fluorine atoms^34,36–41^ or –CF_3_ groups.^21,33,34,36,37,42–50^ A few examples of anionophores containing –OCF_3_ ^28,29,40^ and –SF_5_ ^47,51–54^ groups in their structure have also been reported. Examples containing non-aromatic fluorinated substituents are rare.^35,55^ Moreover, reports with fluorinated transporters usually only include a few derivatives per series.

The effect of substituents on macrocyclic anion transporters is less explored as the preparation of a larger series of macrocycles is more challenging than for simpler anionophores. Macrocyclic anion transporters are often larger, stronger in anion binding, and more lipophilic than simple anion transporters. Examples of macrocyclic anionophores include those based on calixarenes,^26,32,56–60^ calixpyrroles,^61–66^ resorcinarenes,^27,67^ cyclic peptides,^68–70^ biotin[6]urils^71^ and aza- and thiobambusuril derivatives.^72–74^ However, it should be noted that only a few of these reported macrocycles have a fluorinated substituent in their structure.^26,32,61,62,66,67,75^

An outstanding example of macrocyclic anionophores are fluorinated bambusurils^76–78^ (BUs), which have been identified as receptors with particularly high anion affinities and as the most effective Cl^−^/HCO_3_^−^ transporters to date.^14^ These fluorinated BUs transport anions *via* mobile carrier mechanism, *i.e.*, the complex of BU and anion diffuses through the membrane. Interestingly, the Cl^−^/HCO_3_^−^ antiport by BUs could be brought to a halt upon addition of the even stronger bound anions NO_3_^−^ or ClO_4_^−^. We have also reported examples of BU transporters that contain an atypical –SCF_3_ group within their structure.^76,78^ BUs contain 12 benzyl substituents per molecule and therefore small changes to the substituent are amplified by the number of the substituents. Investigating a series of BU derivatives would facilitate the evaluation of the effect of fluorinated substituents on the anion transport properties of larger anion receptors.

**Fig. 1:**
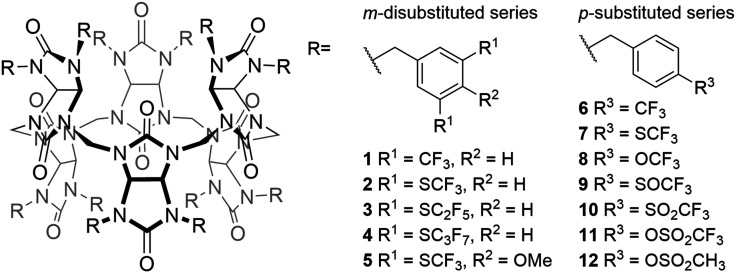
Structures of the investigated bambusurils.

In this work, the anion transport activity of eleven fluorinated BU derivatives and a non-fluorinated analogue across phospholipid bilayers is presented (Fig. 1). BUs 1 and 7 have previously been reported as highly active Cl^−^/HCO_3_^−^ transporters. Here we compare the rates of Cl^−^/HCO_3_^−^ transport by all twelve BUs, showing how slight variations in the substituents impact the transport activity of the fluorinated BUs. Furthermore, we present the impact of changes in the lipid composition as well as a comparison of the transport of different anions.

## Results and discussion

### Design and synthesis

Based on highly active transporter 1, we have recently reported the synthesis and evaluation of the anion binding properties of BUs 2–5.^79^ These thioether derivatives are predicted to be more lipophilic than the parent BU 1 (see Table 1) and the lipophilicity of these derivatives is increased by extending the length of the fluoroalkyl chains on the BU benzyl substituents, going from –SCF_3_ to –SC_2_F_5_ and –SC_3_F_7_ groups for BUs 2, 3 and 4, respectively. BU 5 resembles BU 2 but with addition of a –OMe group on the benzyl substituent, which did however not change the anion binding strength,^79^ and the calculated log *P* values for BUs 2 and 5 suggest that the lipophilicity is not significantly impacted either.

**Table 1:** The properties and anion affinities of the different BUs and their transport rate constants (*k*) and initial transport rates (*I*) for Cl^−^/HCO_3_^−^ antiport with BUs preincorporated at 0.02 mol% in the lucigenin assay

BU	Substituent *para*	Substituent *meta*	MW (g mol^−1^)	Log *P* of single GU^a^	*S* log *P*^b^	*σ*_m_ or *σ*_p_	Log *K*_a_ in MeCN Cl^−^ (M^−1^)	*k* (10^3^ s^−1^) (0% chol.)	*k* (10^3^ s^−1^) (30% chol.)	*I* (10^3^ s^−1^) (0% chol.)	*I* (10^3^ s^−1^) (30% chol.)
1	–H	–CF_3_	3638	6.6	39	0.43	11.2^e^	72	100	33	61
2	–H	–SCF_3_	4408	11.3	54	0.40	11.7^e^	78	12	42	21
3	–H	–SC_2_F_5_	5608	12.6	69	—	—c	11	9	14	8
4	–H	–SC_3_F_7_	6808	15.1	84	—	—c	4	<3	5	<6
5	–OCH_3_	–SCF_3_	4768	11.1	54	—	11.7^f^	31	—d	31	—d
6	–CF_3_	–H	2822	4.8	27	0.54	9.7–10.5	32	6	34	9
7	–SCF_3_	–H	3207	7.1	34	0.50	10.6^f^	17	25	14	35
8	–OCF_3_	–H	3014	6.0	26	0.35	9.7	12	13	10	17
9	–SOCF_3_	–H	3497	4.6	22	0.69	11.2^f^	3	—d	4	—d
10	–SO_2_CF_3_	–H	3591	4.7	18	0.96	11.5^f^	—d	—d	—d	—d
11	–OSO_2_CF_3_	–H	3783	5.1	18	0.53	10.5	42	—d	38	—d
12	–OSO_2_CH_3_	–H	3135	0.7	7	0.36	9.0^f^	—d	—d	—d	—d

a Calculated in ChemDraw (Fig. S43†).

b Calculated using TorchLite (ESI, section 5†).

c Not determined.

d No transport observed.

e Value from ref. 79.

f Value from ref. 80. Errors on the log *K*_a_ values are ±0.2–0.3 and errors on the quantification of the transport rate constants (*k*) and initial transport rates (*I*) are up to 15%.

The second series of BUs investigated here consists of BU derivatives with *para*-functionalized benzyl substituents. Herein, BUs 7, 9, 10 and 12 have been previously reported as anion receptors.^80^ Moreover, BU 7 was also shown to be an effective anionophore.^76^ The –SCF_3_ group in BU 7 was replaced by –CF_3_ and –OCF_3_ groups to obtain BUs 6 and 8, respectively. It is anticipated that this alternation will result in a variation in lipophilicity and anion binding strength when compared to BU 7. BU 11 was synthesised for comparison to 10, with an additional oxygen atom placed in between the –SO_2_CF_3_ group and the benzyl substituent, resulting in a –OSO_2_CF_3_ group. This variation is predicted to lead to a reduction in anion binding strength according to the *σ*_p_ Hammett substituent parameter (Table 1) and calculated electrostatic potential map (Fig. S44†). BU 12 serves as a non-fluorinated analogue of 11.

**Fig. 2:**
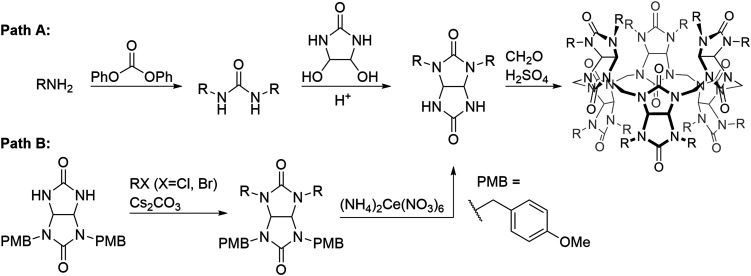
General synthetic routes towards the BU derivatives discussed in this work.

The BU anionophores presented in this work were obtained utilizing a macrocyclization reaction of glycolurils (GU) and formaldehyde in the presence of sulphuric acid, which acted both as an acid catalyst and a source of HSO_4_^−^ as template, promoting the formation of a six-membered macrocycle (Fig. 2). The GU building blocks were prepared using one of the two synthetic strategies shown in Fig. 2. In the first strategy (Path A), a substituted benzylamine was used as the starting material. It was first reacted with diphenyl carbonate to give a 1,3-disubstituted urea. The urea was then condensed under acidic conditions with 4,5-dihydroxy-2-imidazolidinone, leading to a GU. When a substituted benzyl halide (chloride or bromide) was used as the starting material, this was reacted with a GU bearing two *para*-methoxybenzyl (PMB) protecting groups under basic conditions (Path B). The PMB groups were subsequently cleaved using ceric ammonium nitrate. The GU building blocks for BUs 1 and 6–8 were prepared using Path A;^76^ Path B was used to prepare GU building blocks for BUs 2–5 and 7.^78,79^ GU building blocks for BUs 9 and 10 were prepared by oxidation of the GU building block for BU 7.^80^ GU building blocks for BUs 11 and 12 were prepared by functionalization of GU bearing *para*-hydroxybenzyl groups with triflyl or mesyl groups, respectively.^80^ BUs 6, 8 and 11 are prepared as new compounds and their detailed synthetic procedures and characterisation can be found in the ESI (section 1†).

### Anion binding studies

The binding strength of the BUs to chloride was studied using NMR spectroscopy in CD_3_CN, with tetrabutylammonium (TBA) serving as the counter cation. Given the strong binding of chloride by BUs 1–12, which precludes the use of a single titration experiment for the determination of the association constant *K*_a_, competition experiments were required. Another complication arises from the difficulties to obtain BUs 2–4 and 9–10 without any anion inside their cavity. However, exchange for another anion was feasible, meaning that the templating HSO_4_^−^ anion could be replaced by Cl^−^.

A series of titrations with different anions was carried out for anion free BU 7. In these, BU 7 was first titrated with TBA^+^CF_3_SO_3_^−^, monitored by both ^1^H and ^19^F NMR spectroscopy and the changes in the chemical shifts were analysed by a 1 : 1 binding model to obtain the *K*_a_ of BU 7 for CF_3_SO_3_^−^. Subsequently, BU 7 was titrated with ReO_4_^−^ in the presence of excess CF_3_SO_3_^−^, enabling the determination a selectivity ratio for the two anions and thus of the *K*_a_ of BU 7 for ReO_4_^−^. Further competition studies with ClO_4_^−^ and Cl^−^ resulted in the determination of *K*_a_ = (4 ± 2) × 10^10^ M^−1^ between BU 7 and Cl^−^.^80^ Anion binding data for non-fluorinated BU 12 were obtained using this strategy as well.^80^

BU 7 was then used to determine *K*_a_ values of BUs 1, 2, 5, 6 and 8–11 towards Cl^−^ in experiments where 7 competed with other BUs for the chloride anions, as analysed using ^19^F NMR spectroscopy.^79,80^ In these studies BU 7 was used as an anion-free compound, while the other BU was introduced as either a ∼1 : 1 complex with TBACl or as anion-free compound, in which case a small amount of TBACl was added to the system. The sum of the concentrations of both BUs was larger than that of chloride, ensuring that all chloride was distributed between the two BUs according to their relative ratio in *K*_a_ values. This relative difference was then calculated from the areas corresponding to the signals of anion-free and anion-bound BUs. Unfortunately, the *K*_a_ values of BUs 3 and 4 could not be obtained as the peaks of anion-free and complexed BU overlapped.^79^ A similar problem was encountered for competition experiments with BU 6, for which the peaks corresponding to complexed and anion-free 6 were poorly resolved, allowing only a more rough estimation of its *K*_a_ to chloride. The ^10^logartim of all *K*_a_ values are reported in Table 1.

**Fig. 3:**
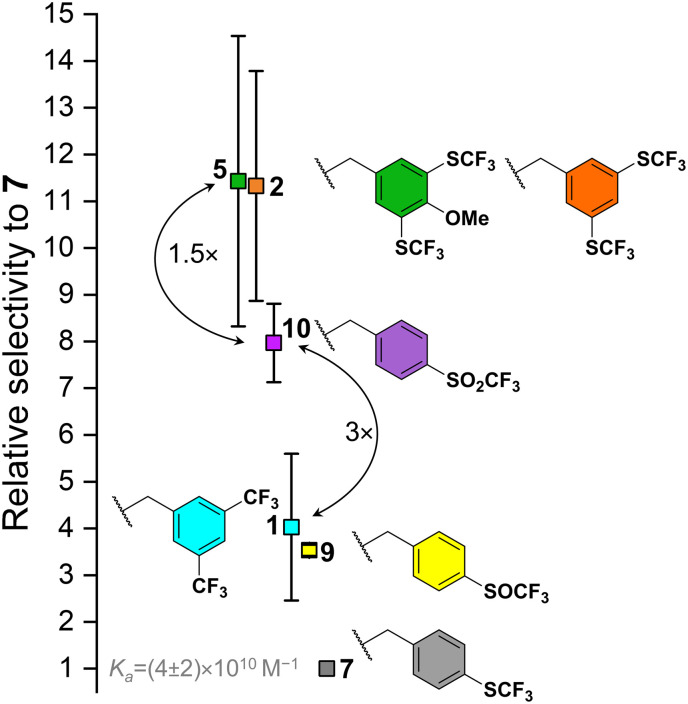
The relative binding affinities of BUs towards chloride in CD_3_CN with respect to BU 7 as determined by competition experiments (average values and standard deviations of 4–9 experiments); BUs binding chloride weaker than 7 are not shown.

In general, the increase in electron-withdrawing effect of the fluorinated groups on the BU benzyl substituent causes an increase in the *K*_a_ of BU to anions. Fig. 3 provides a visual comparison of BUs binding chloride stronger than 7 with error bars representing standard deviations from the competition experiments between the individual BUs and 7. Despite the very strong binding of chloride by all the BUs, BUs 2 and 5 can be clearly distinguished as the strongest receptors of the series. Even though it was impossible to determine *K*_a_ values for BUs 3 and 4, it is expected, that they would possess *K*_a_ values comparable to those of 2 and 5, as the increase in fluoroalkyl chain length is not expected to induce a substantial alternation in the electron-withdrawing properties of the substituent. The direct competition experiment between BUs 5 and 10 confirmed that 10 binds chloride 1.5-fold weaker than 5. A lower binding strength was found for BUs 1 and 9 (both having a similar *K*_a_ to chloride), followed by 7. BUs 6, 8, 11 and 12 were found to be weaker in chloride binding than 7 (Table 1). We note that the estimated *K*_a_ for BU 6 is lower than for 7, which corresponds well to the result obtained for BUs 1 and 2, where the derivative with –SCF_3_ groups (2) has stronger binding than the one with –CF_3_ (1) groups.

It is important to note that the absolute values of the *K*_a_ values for all BUs in this series (except for 12) are based on the *K*_a_ of BU 7 to chloride. Consequently, any absolute error in the determination of this *K*_a_ would result in a shift of the *K*_a_ of all BUs to either higher or lower absolute values, yet the selectivities of the BUs for chloride would remain unaltered.

### Cl^−^/HCO_3_^−^ transport studies in POPC liposomes

**Fig. 4:**
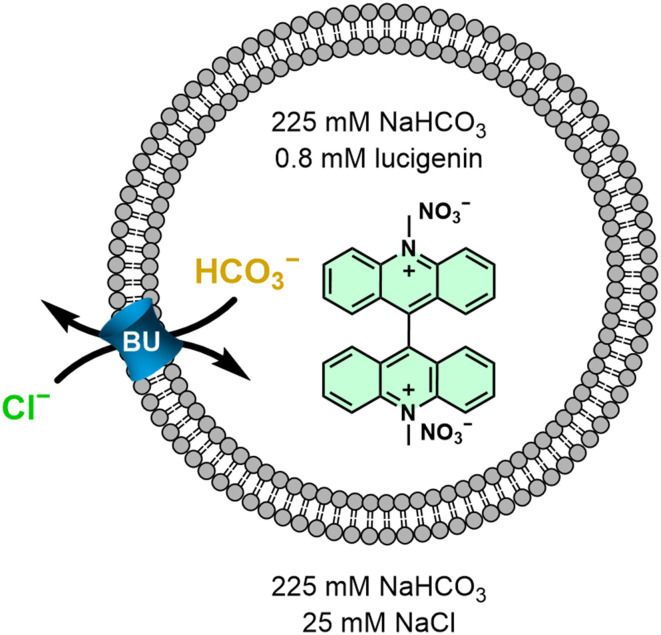
Schematic representation of the lucigenin assay.

**Fig. 5:**
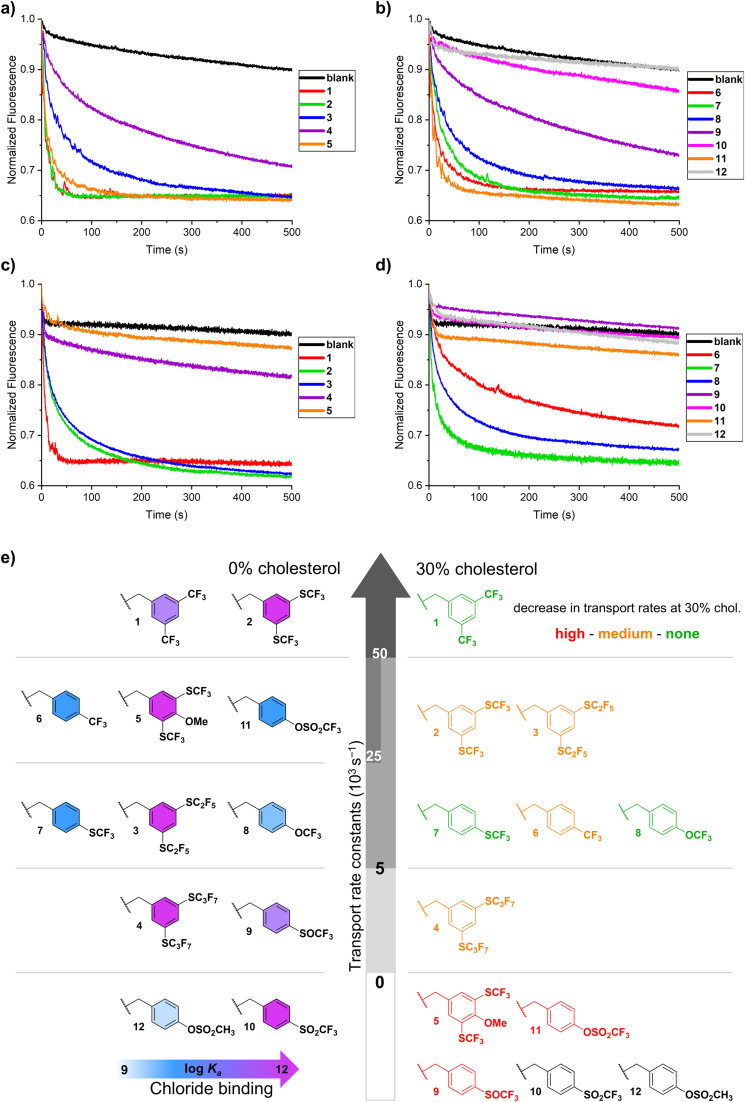
The transport of chloride *via* Cl^−^/HCO_3_^−^ antiport by BUs (pre-incorporated at 0.02 mol%) monitored by the lucigenin assay in 225 mM NaHCO_3_, upon addition of 25 mM NaCl to LUVs composed of either POPC only (a and b), or POPC/cholesterol 7 : 3 (c and d); control experiments without any BU are indicated as ‘blank’; (e) graphical visualization of the anion transport rate constants at 0% (left part) and 30% (right part) cholesterol content in the membrane, with the binding strength to chloride indicated in the left part (light blue = the weakest binding, purple = the strongest binding) and the effect of cholesterol on transport rates indicated in the right panel (red = transport stopped, orange = transport decreased, green = no significant effect).

The ability of the twelve BU derivatives to perform Cl^−^/HCO_3_^−^ antiport activity was investigated in large unilamellar vesicles (LUVs) using the lucigenin assay (Fig. 4).^81^ The chloride-sensitive probe lucigenin was encapsulated in the vesicles to monitor the influx of chloride anions into the LUVs mediated by BUs. LUVs with a diameter of approximately 180 nm (Fig. S37†) were prepared from 1-palmitoyl-2-oleoyl-*sn*-glycero-3-phosphocholine (POPC) with the BUs preincorporated in the membranes at a concentration of 0.02 mol% with regard to the total lipid amount. The first series of experiments was performed without cholesterol in the membranes, after which the anion transport experiments were repeated with 30% cholesterol (*vide infra*). LUVs containing encapsulated lucigenin were dispersed in a NaHCO_3_ solution (225 mM interior and exterior) and the experiment was initiated by adding a NaCl solution (25 mM) to the LUV exterior to establish a chloride gradient. A quenching of the fluorescence of lucigenin over time can be observed, which is due to the transport of chloride anions into the LUVs (Fig. 5a–d).

For quantification of the obtained transport data, they were plotted as the inverse of the normalised curves (*F*_0_/*F*, see the ESI Fig. S38†) which corresponds to the chloride concentrations inside the LUVs. The plotted curves were analysed using single and double exponential functions to obtain rate constants and initial transport rates, respectively (Table 1 and Fig. 5e). The results from the competition studies described above were used for the visual categorisation of the Cl^−^ affinities of the BUs in the left panel of Fig. 5e, ranging from the weakest (light blue colour) in binding to the strongest ones (purple colour).

BUs 1 and 2 were clearly the most active compounds of the series of BUs with *meta*-substituted benzyl groups (red and green curves in Fig. 5a). No clear difference could be made in the activity of BUs 1 and 2 at a concentration of 0.02 mol%. Therefore, the transport experiment was repeated at a 10-fold lower BU concentration (0.002 mol%) indicating that BU 1 was more active than 2 (Fig. S39, Table S4†). BU 2 has a higher lipophilicity and binding strength than 1, and the impacts of small changes in these two parameters on transport activity might be counteracting. The higher lipophilicity of BU 2 could enhance the diffusion rate through the apolar membrane interior. However, the very high binding strength of BU 2 could slow down the release of the anion at the membrane interface. We note that for compound 1 anion release was previously found to be rate limiting in the transport process.

As the lipophilicity and size of BUs increased when going from 2 to 3 and 4, a clear decrease in transport activity was found (green, blue and violet curves in Fig. 5a). A too high lipophilicity might prevent BU from residing at the membrane/aqueous interface, preventing the effective exchange of the anions with the aqueous solution. Additionally, a too large size might slow down the diffusion of the BU through the membrane.

The presence of the –OMe group in BU 5 resulted in a slight decrease in activity in comparison to 2, as concluded from the obtained transport curves (orange and green curves in Fig. 5a) and fitted transport rate constants (Table 1) and confirmed from an experiment at a 10-fold lower concentration (Fig. S39, Table S4†). The polar –OMe group could interact with lipid headgroups or be hydrated at the membrane interface, inducing a penalty to the transport process by BU 5 as compared to 2. A decrease in transport activity for compounds containing ether groups was already observed for decalin-based transporters.^10^

BUs 6–8 exhibited similar anion transport rates (red, green and blue curves in Fig. 5b), with 6 transporting slightly faster than 7 and 8 (Table 1). It can be deduced from the results that the variation of the –CF_3_ group to either –SCF_3_ or –OCF_3_ does not induce a significant change in the overall anion transport ability. This result corresponds to the similar activities found for BUs 1 and 2, which bear two –CF_3_ or –SCF_3_ groups per benzyl substituent. The lipophilicity of BUs 6 and 8 is predicted to be similar, with 7 being slightly more lipophilic, while the differences in the *K*_a_ values for chloride between these three BUs are also within one order of magnitude.

When the –SCF_3_ group in BU 7 was altered to its oxidised sulfoxide and sulfone variants, a decrease in the transport activity was found for 9, while for 10 no significant transport was observed (green, violet and pink curves in Fig. 5b). On one hand, an increase in the binding strength can slow down the transport process if binding is too strong, but on the other hand, the *K*_a_ of BU 2 for Cl^−^ is higher than that of either 9 or 10, yet 2 is still among the most active anionophores. An alternative explanation for the lower activity of BUs 9 and 10 can be proposed by considering that –SOCF_3_ and –SO_2_CF_3_ groups could be strongly hydrated, thereby impeding the detachment of the corresponding BUs from the membrane interface as required for the diffusion through the membrane.

In contrast to the inactive sulfone-containing BU 10, BU 11 with triflate groups was found to be a highly active anionophore (orange curve in Fig. 5b). This result is quite surprising given the only subtle differences between these two compounds. The lipophilicity of BUs 10 and 11 is predicted to be the same by TorchLite (Table 1), while the chloride binding strength of 11 is around one order of magnitude lower than that of 10. However, as observed for BUs 1–2 and 6–8, such minor variations in binding strength of these very strongly binding macrocycles should not affect the transport capability of the BUs. The oxygen atom positioned between the triflyl group and the benzene ring in BU 11 affects the orientation of the triflyl group towards the benzene ring in comparison to 10. This variation could potentially influence the accessibility of the oxygen atoms in the triflyl group to form hydrogen bonds. A comparison of BU 11 with most active compounds 1, 2, and 5 at a 10-fold lower concentration confirmed that 11 was almost as active as 1 (Fig. S39, Table S4†). In contrast, BU 12, the non-fluorinated analogue of BU 11, did not exhibit any transport activity. Based on chloride binding strength and predicted lipophilicities of these two compounds, it is possible that the lipophilicity of BU 12 and/or the *K*_a_ of 12 for Cl^−^ are too low to be an effective anionophore.

Several conclusions can be drawn when considering the transport results obtained for all twelve BU derivatives. Changes (up to two orders of magnitude) in the BU anion binding strength do not seem to affect the transport significantly. However, the overall high affinity of the fluorinated BU macrocycles to anions might be important for effective transport. Conversely, a too high lipophilicity or a too large size cause a decrease in the transport activity. The preferred conformation of the BU substituents could be affected by the fluorinated groups and may also be a contributing factor, as it should have an impact on the shape and lipophilic surface of the individual BU derivatives. Unfortunately, this is not readily evaluated, especially as the lipid membrane is a complex environment and the substituents are flexible and relatively free to rotate.

### Cl^−^/HCO_3_^−^ transport studies with cholesterol present in the membrane

We have discovered in our recent work on BUs conjugated with bile acid residues that their transport activity was drastically impacted by an increased cholesterol content in the membrane.^82^ Therefore, we were curious whether the anion transport by the fluorinated BUs investigated in this work would be impacted as well. We have thus tested the Cl^−^/HCO_3_^−^ antiport activities of all BUs using the lucigenin assay with BUs preincorporated at 0.02 mol% inside the membrane of LUV composed of POPC and cholesterol in a molar ratio of 7 : 3 (Fig. 5c and d).^83^ Strikingly, the presence of cholesterol in the membrane has a drastic negative impact on the anion transport by particular compounds, while other derivatives remain unaffected or are only slightly affected (Fig. 5e, right panel).

**Fig. 6:**
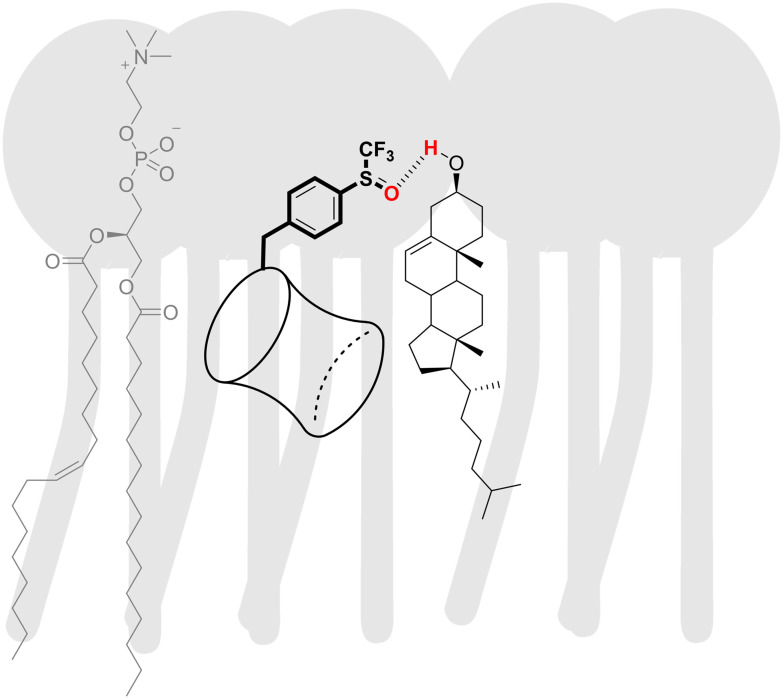
A schematic representation of an interaction between a polar BU substituent and cholesterol in the membrane.

The sensitivity of the anion transport by BUs to the cholesterol content in the membrane appears to be associated with the presence of hydrogen bond accepting groups on the BU substituents. For instance, BUs 5 and 11 are highly active in the LUVs made of pure POPC, but complete inactive in the presence of cholesterol. Similarly, moderately active BU 9 lost its activity in the presence of cholesterol. BU 8, on the other hand, was not impacted despite containing an oxygen atom in the substituent. Potential reasons could be the steric hindrance by the –CF_3_ group or a decrease in the electron density on the oxygen atom, both of which would reduce the hydrogen bond accepting ability of the oxygen. We hypothesise that the –OH group of the cholesterol might interact with these hydrogen bond acceptors in BUs 5, 9, and 11, as visualised schematically in Fig. 6.

BUs 2–4 and 6 exhibited a slight decrease in their transport activities in LUVs composed of POPC/cholesterol, as is commonly observed for anionophores.^82^ As the BU anionophores are relatively large molecules, a tighter packing of the lipids with cholesterol can slow down the diffusion of the BUs through the membrane.

### Transport studies of BU 1 in liposomes of different lipid compositions

Interestingly, the transport activity of the most active BU 1 remained unaffected by the presence of cholesterol in the membrane. Therefore, we were curious if changes in the membrane thickness could influence the transport activity of BU 1. The transport rates of 1 in LUVs made of phosphocholines (PCs) with two monounsaturated fatty acids with varied chain lengths were measured. If the exchange of anions at the membrane periphery would be the rate-limiting step of the transport process, the transport rates should be similar regardless of the membrane thickness. On the other hand, a decrease in the transport activity with increasing length of the PC acyl chains would suggest that the diffusion of 1 is the rate-determining step of the transport process.

**Fig. 7:**
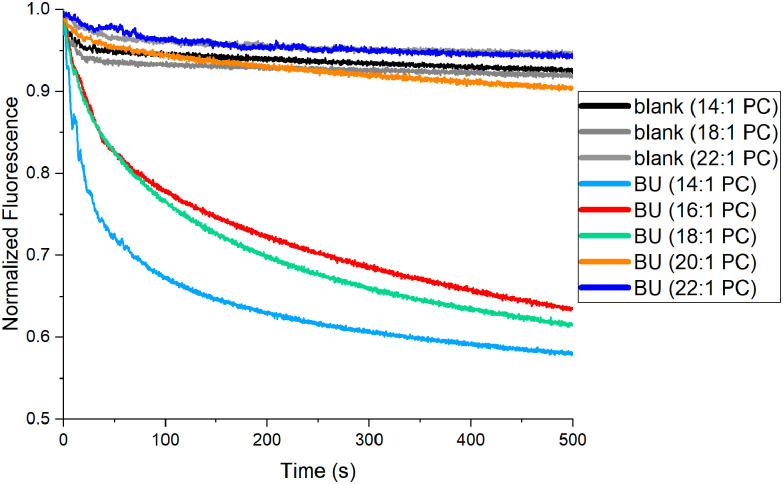
Cl^−^/NO_3_^−^ antiport activity of BU 1 through membranes of varying thickness measured using the SPBA assay, the BU was preincorporated at 0.1 mol% in the LUVs made of PC/cholesterol 7 : 3, LUVs were suspended in 225 mM NaNO_3_ (internal and external) at 0.2 mM total lipid concentration, the transport was initiated with 25 mM NaCl pulse. Control experiments without BU 1 are indicated as ‘blank’.

An initial attempt to study Cl^−^/HCO_3_^−^ antiport by 1 in these liposomes was unsuccessful as the runs in absence of 1 revealed significant leakage of lucigenin through the membrane, making these experiments unreliable. Therefore, we have switched to measuring Cl^−^/NO_3_^−^ antiport activity of 1 using *N*,*N*′-bis(3-sulfonatopropyl)-9,9′-biacridinium (SPBA), a more hydrophilic analogue of lucigenin, as fluorescent probe.^81^ The experiments were conducted in a homologous series of PCs (with 14 : 1, 16 : 1, 18 : 1, 20 : 1 and 22 : 1 acyl chains), in LUVs made of PC/cholesterol 7 : 3 (Fig. 7). It was observed that in the LUVs with the thinnest membrane (14 : 1 PC, light blue curve) the transport is the fastest, while it became slightly slower in the LUVs made of 16 : 1 and 18 : 1 PC (red and green curves, respectively). Interestingly, transport completely stopped in LUVs with thicker membranes (20 : 1 and 22 : 1 PC; orange and blue curves, respectively). This finding could suggest channel-like behaviour for 1, which is, however, unlikely based on the inactivity of 1 in LUVs made of DPPC at temperatures below the DPPC transition temperature.^76^ Our trend found for 1 is different from the results obtained for cholapod anion transporters, for which a steady decrease of the transport rates with increasing PC acyl chain lengths was observed.^31^ A decrease in the transport rate with increasing membrane thickness was also observed for anion transport relays.^84,85^ However, it should be noted that the mechanism of transport by these relays is different from the one by BUs.

### Transport of different anions by BU 1

The studies of the Cl^−^/NO_3_^−^ antiport activity described above had to be carried out at a higher concentration of 1 to obtain clear transport curves. This is in line with previous observations that the Cl^−^/NO_3_^−^ antiport process by fluorinated BUs is much slower than Cl^−^/HCO_3_^−^ antiport. This was attributed to the possibility of formation of a 2 : 1 complex of BU 1 with Cl^−^ and HCO_3_^−^ anions, removing the need for full decomplexation of the BU during the transport process.^76^ Moreover, the high affinity of BU 1 towards NO_3_^−^ and selectivity for NO_3_^−^ over Cl^−^ disfavour the decomplexation of NO_3_^−^. The same trend was observed for HCO_3_^−^/Cl^−^, which was significantly faster than HCO_3_^−^/NO_3_^−^ antiport in experiments using the [Eu·L^1^]^+^ probe to directly monitor HCO_3_^−^ transport into LUVs.^77^ The results for the selectivities of transport processes by BU 1 obtained from these two assays show that the relative amounts or absolute concentrations of anions present in the medium do not play a significant role. For example, 10 mM HCO_3_^−^ was used in the [Eu·L^1^]^+^ assay (added to 225 mM NaCl), while 225 mM NaHCO_3_ was used in the lucigenin assay (to which 25 mM NaCl was added), resulting in similar rates of Cl^−^/HCO_3_^−^ antiport.

To further investigate the relationship between the selectivity of BU 1 to different anions and the selectivities of the transport processes, we investigated Br^−^/NO_3_^−^, I^−^/NO_3_^−^, Br^−^/HCO_3_^−^ and I^−^/HCO_3_^−^ antiport by 1, using the lucigenin and SPBA assays. Additionally, Cl^−^, NO_3_^−^, and SO_4_^2−^ uniport activities by 1 were measured using the HPTS assay. Other previously investigated transport processes by BU 1 were Cl^−^/AcO^−^ and Cl^−^/SO_4_^2−^ antiport^76,81^ and the transport of F^−^.^86^ All these transport processes by BU 1 are summarised in Table 2.

**Table 2:** Ranking of different anion transport processes by BU 1

Definition	Transport classification	Antiport	Uniport
100% transport in <500 s at low conc. (≤0.004 mol%)	Exceptionally good	Cl^−^/HCO_3_^−^a^,^^76^, HCO_3_^−^/Cl^−^b^ ,^^77^	Cl^−^ c^,^^d^, HCO_3_^−^ b^ ,^^77^, NO_3_^−^ c^,^^d^
100% transport in <500 s at medium conc. (0.1 mol%)	Good	Cl^−^/NO_3_^−^ a^,^^76^, Br^−^/NO_3_^−^ a^,^^d^, I^−^/NO_3_^−^ a^,^^d^, Br^−^/HCO_3_^−^ a^,^^d^	
50%–100% transport in <500 s at medium conc. (0.1 mol%)	Medium	F^−^/Cl^−^ b^,^^86^, F^−^/NO_3_^−^ b^,^^86^, Cl^−^/AcO^−^ a^,^^76^, I^−^/HCO_3_^−^ a^,^^d^	F^−^ b^ ,^^86^
<50% transport in <500 s at medium conc. (0.04–0.1 mol%)	Poor	Cl^−^/SO_4_^2−^ a^ ,^^81^, HCO_3_^−^/NO_3_^−^ b^,^^77^	
No transport observed, even at high concentration	None		OH^−^ c^ ,^^86^ (or H^+^), SO_4_^2−^ c^,^^d^
		

a Lucigenin or SPBA assays.

b [Eu·L^1^]^+^ assay.

c HPTS assay.

d This work.

The ability of BU 1 to transport halides (Cl^−^, Br^−^, I^−^) in nitrate or bicarbonate solutions was investigated using the lucigenin and SPBA assays, as all three halides are known to quench the fluorescence of these fluorescent probes. BU 1 was preincorporated in LUVs made of POPC and cholesterol (7 : 3 ratio) at 0.1 mol%. The transport experiments were initiated with the addition of 25 mM NaX. It should be noted that I^−^ and Br^−^ quench lucigenin and SPBA stronger than Cl^−^, resulting in lower fluorescence levels at the final plateaus of the transport experiments compared to the experiments with Cl^−^.

**Fig. 8:**
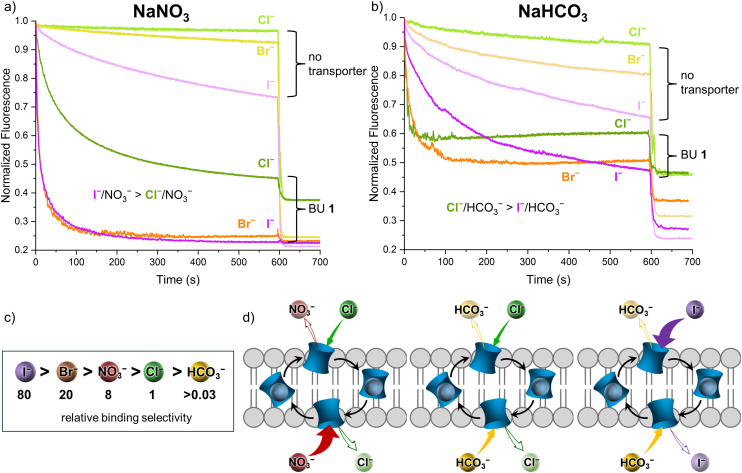
(a) X^−^/NO_3_^−^ antiport activity of BU 1 measured using the SPBA assay in 225 mM NaNO_3_ (internal and external); (b) X^−^/HCO_3_^−^ antiport activity of BU 1 measured using the lucigenin assay in 225 mM NaHCO_3_ (internal and external). BU **1** was preincorporated at 0.1 mol% in the LUVs made of POPC/cholesterol 7 : 3 at 0.4 mM total lipid concentration. The transport was initiated with a 25 mM NaX pulse; (c) schematic representation of anion binding selectivity for BU 1; (d) schematic representation of the anion exchange during antiport processes.

Based on the hydration energies and the Hofmeister series, it was expected that the transport rates would be in the order I^−^ > Br^−^ > Cl^−^.^87^ Indeed, this trend was found when the transport experiments were done in NaNO_3_ solution. I^−^/NO_3_^−^ and Br^−^/NO_3_^−^ antiport processes were observed to be considerably faster (*k* of 0.013 and 0.023 s^−1^ respectively) than Cl^−^/NO_3_^−^ antiport (0.006 s^−1^) by BU 1 (Fig. 8a). On the other hand, a rather surprising trend was found in the NaHCO_3_ solution (Fig. 8b). The Cl^−^/HCO_3_^−^ antiport by 1 is the fastest (*k* of 0.126 s^−1^), followed by Br^−^/HCO_3_^−^ antiport (0.017 s^−1^) and very slow I^−^/HCO_3_^−^ antiport (0.004 s^−1^). Furthermore, the experiments with hydrophobic I^−^ show that I^−^ can permeate the LUV membrane to some extent even without any anion transporter (light purple curves in Fig. 8), meaning that part of the observed I^−^/HCO_3_^−^ antiport is due to spontaneous diffusion of I^−^, making this process slower than Cl^−^/NO_3_^−^ antiport.

It is expected that due to such high affinities of BU 1 to anions, decomplexation does not occur at the membrane interface, but a direct exchange of the anions would be preferred (Fig. 8d).^33^ In this case, a balanced selectivity of 1 for both anions involved in the antiport process would be beneficial. The anion binding selectivity of 1 in acetonitrile is following the order: I^−^ > Br^−^ > NO_3_^−^ > Cl^−^ > HCO_3_^−^ (see Fig. 8c, *K*_a_ values provided in Table S3†). This would match the observation that Cl^−^/HCO_3_^−^ antiport, with both anions having similar *K*_a_ values (less than 30-fold difference) is faster than I^−^/HCO_3_^−^ antiport where a larger binding selectivity of BU 1 for I^−^ is present (up to 2400-fold). Inversely, I^−^/NO_3_^−^ and Br^−^/NO_3_^−^ antiport are more efficient as both anions are very strongly bound in these cases. It was hinted previously for more structurally simple anionophores that a balanced selectivity for binding and transport of individual anions gives the fastest exchange rates in the antiport process.^88^

As mentioned above, F^−^ can also be transported by 1 across lipid bilayers as investigated previously using the emissive probe [Eu·L^1^]^+^ to directly monitor the concentration of F^−^ inside LUVs (which cannot be done with lucigenin or SPBA).^86^ F^−^ uniport, F^−^/Cl^−^ and F^−^/NO_3_^−^ antiport processes were found to be possible by 1 (Table 2). Nevertheless, when considering halides, F^−^ is the slowest to be transported, which might be linked to the low affinity that BU macrocycles have for F^−^ compared to other halides in combination with the strong solvation of F^−^.^89,90^

Electrogenic uniport of Cl^−^, NO_3_^−^or SO_4_^2−^ by 1 was investigated using the HPTS assay.^91^ LUVs of ∼180 nm diameter were prepared from POPC and cholesterol in a 7 : 3 molar ratio with 1 preincorporated in the membranes at various concentrations. Instead of Na^+^, protonated *N*-methyl-d-glucamine (NMDGH^+^) was used as non-transportable counter cation for both the internal and external aqueous phases.^92^ LUVs with encapsulated HPTS were dispersed in NMDGH^+^ solution with the counter anion of which the transport was to be studied (100 mM internal and external), containing HEPES (10 mM) at pH 6.8. The experiment was initiated by adding an NMDG solution (5 mM) to the exterior of the LUVs, creating a pH gradient across the vesicle membrane.

Previously we have reported rather low activities for Cl^−^ and NO_3_^−^ uniport by 1 when the proton channel gramicidin was used for proton transport.^76^ However, we found much higher Cl^−^ uniport rates when employing carbonyl cyanide *m*-chlorophenyl hydrazone (CCCP) as a protonophore.^86^ Indeed, 1 exhibited a high activity as a uniporter of both Cl^−^ and NO_3_^−^, even at low concentrations (0.002 mol%, Fig. S41a and S42a†). An intriguing difference was observed when Cl^−^ uniport by 1 was tested using the HPTS assay in NaCl instead of NMDG H^+^Cl^−^ solution. In NaCl, the Cl^−^ uniport by 1 was found to be considerably slower than in NMDGH^+^Cl^−^ solution (Fig. S41b†), suggesting that cations might have an impact on the anion transport properties of BUs. However, it is the question whether they are directly involved in the transport process or if they might impact anion exchange at the membrane periphery by BUs. This phenomenon is a subject of future investigations.

Sulfate uniport by 1 was not observed even at 0.1 mol% concentration of 1 in either Na^+^ or NMDGH^+^ sulfate solutions (Fig. S42b†). However, slow but clear Cl^−^/SO_4_^2−^ antiport by 1 was observed using the lucigenin assay.^81^ The ability of 1 to transport SO_4_^2−^ in this case may be driven by the Cl^−^ transport, resulting in the creation of a charge gradient across the membrane. The transport of highly hydrated SO_4_^2−^ is not easy, and it is generally assumed, that it cannot be transported. Only a few urea-based transporters have been reported to be able to transport this anion.^23,34,68,93,94^

## Conclusions

In this work, we have investigated the anion transport properties of twelve BU derivatives, only two of which had been previously studied and three of which were prepared for the first time. Measurements of their Cl^−^/HCO_3_^−^ antiport activities in the lucigenin assay provided a comprehensive study of the effect of variation of substituents on large macrocyclic anionophores on their transport properties. As the affinities of the tested BUs to anions are generally very high, the absolute *K*_a_ values seem not to be an important parameter, in contrast to what is commonly observed in the literature for smaller compounds. All tested BUs (apart from 12) are predicted to be highly lipophilic and further increases in the lipophilicity were found to have a negative impact on the transport abilities.

The complexity of the BU anionophores is further highlighted by their strongly varied response to the addition of cholesterol to the membrane compositions, where compounds with hydrogen bond accepting oxygen atoms seem to be most impacted. Further investigations are therefore required in the context of biological applications,^95^ as cell membranes are much more complex than the model LUVs used here. Despite several variations in the BU substituents, 1 remains the most active BU macrocycle-based transporter to date and the most active Cl^−^/HCO_3_^−^ antiporter known.^14^ The study of different antiport processes between halides and oxoanions by 1 has shown that a balanced binding selectivity for the transported anions is preferred for an effective antiport process by these BUs with very high anion affinities.

## Conflicts of interest

There are no conflicts to declare.

## Supplementary Material

OB-023-D5OB00400D-s001

## Data Availability

The datasets supporting this article have been uploaded as part of the ESI.†
