# Supplementary material for: Substituent effects of fluorinated bambusurils on their anion transport
Source: Org Biomol Chem. 2025 Apr 10;23(43):9908–21. doi: 10.1039/d5ob00400d (PMC12001018; doi:10.1039/d5ob00400d)
Supplement: OB-023-D5OB00400D-s001 [file OB-023-D5OB00400D-s001.pdf]

Supplementary Information (SI) for Organic & Biomolecular Chemistry.
This journal is © The Royal Society of Chemistry 2025

Electronic Supplementary Information

Substituent effects of fluorinated bambusurils on their anion transport

Matúš Chvojka,[a,b,c] Vladimír Šindelář[a,b*] and Hennie Valkenier[c*]

[a.] *Department of Chemistry, Faculty of Science, Masaryk University, Kamenice 5, 625 00 Brno, Czech Republic.*

[b.] *RECETOX, Faculty of Science, Masaryk University, Kamenice 5, 625 00 Brno, Czech Republic.*

[c.] *Engineering of Molecular NanoSystems, École Polytechnique de Bruxelles, Université libre de Bruxelles, Avenue F.D. Roosevelt 50, CP165/64, 1050 Brussels, Belgium.*

*E-mail: sindelar@chemi.muni.cz, hennie.valkenier@ulb.be

Table of Contents

1. Synthesis of new compounds S3
1.1. Materials and methods S3
1.2. Bambusuril **6** S3
1.2.1. 1,3-Bis(4-(trifluoromethyl)benzyl)urea **6b** S3
1.2.2. 2,4-Bis(4-(trifluoromethyl)benzyl)glycoluril **6c** S4
1.2.3. Dodekakis(4-(trifluoromethyl)benzyl)bambus[6]uril **6** S4
1.3. Bambusuril **8** S5
1.3.1. 1,3-Bis(4-(trifluoromethoxy)benzyl)urea **8b** S5
1.3.2. 2,4-Bis(4-(trifluoromethoxy)benzyl)glycoluril **8c** S6
1.3.3. Dodekakis(4-(trifluoromethoxy)benzyl)bambus[6]uril **8** S6
1.4. Bambusuril **11** S7
1.4.1. 2,4-Bis(4-(triflyloxy)benzyl)glycoluril **11b** S7
1.4.2. Dodekakis(4-(triflyloxy)benzyl)bambus[6]uril **11** S8
2. Characterization S9
2.1. **6b** S9
2.2. **6c** S11

2.3. **6** S13
2.4. **8b** S15
2.5. **8c** S17
2.6. **8** S19
2.7. **11b** S21
2.8. **11** S23
3. Description of the competition experiment between two BUs S25
3.1. Experimental S25
3.2. Association constants obtained from competition experiments S26
3.3. Examples of the results obtained from the competition experiment S27
4. Anion transport experiments S29
4.1. Materials and methods S29
4.1.1. Lucigenin assay to study Cl^-/HCO_3^- antiport S30
4.1.2. Lucigenin and SPBA assays to study X^-/NO_3^- antiport by **1** S30
4.1.3. SPBA assay to study Cl^-/NO_3^- antiport by 1 in liposomes made of various length lipids S31
4.1.4. HPTS assay to study A^- uniport by **1** S31
4.1.5. Analysis of the transport curves obtained for studies using lucigenin or SPBA assays S31
4.2. Results obtained from the lucigenin assay S33
4.3. Results obtained from the HPTS assay for **1** S34
5. Computational studies S35
6. References S36

1. Synthesis of new compounds

1.1. Materials and methods

All reagents and solvents were purchased from commercial suppliers and used without further purification. Synthesis of **1-5**, **7**, **9, 10**, **12** and **11a** was reported previously.[1,2] Mili-Q grade water was prepared by a Barnstead™ MicroPure™ Water Purification System. All reactions that require increased temperature were heated with a DrySyn heating block on an electromagnetic stirrer. Mixing of the reaction mixtures was done by a magnetic stirrer. Reactions exposed to microwave irradiation were placed in suitable pressurized vials and irradiated in Discovery SP microwave reactor. Separation using flash chromatography was performed on CombiFlash® NextGen 300+ (Teledyne ISCO).

The NMR spectra were measured on one of the following spectrometers at 25 °C: Bruker Avance III 300 MHz (^{1}H: 300 MHz, ^{19}F: 282 MHz), Bruker Avance III 500 MHz (^{1}H: 500 MHz, ^{13}C: 126 MHz, ^{19}F 471 MHz), JEOL JNM-ECZ400R/S3 (^{1}H: 400 MHz, ^{13}C: 101 MHz) or Jeol JNM-ECZ600R/S3 (^{1}H: 600 MHz, ^{13}C: 151 MHz, ^{19}F 565 MHz). Chemical shifts (δ) are reported in parts per million (ppm) and coupling constants (*J*) are given in Hertz (Hz). The ^{1}H and ^{13}C NMR spectra were referenced to the solvent residual signals; ^{19}F NMR spectra were unreferenced.[3] The NMR spectra were processed using MestReNova 15.0.0.

HRMS spectra were recorded on an Agilent 6224 Accurate-Mass TOF mass spectrometer. Samples were ionized by electrospray ionization (ESI) or atmospheric-pressure chemical ionization (APCI). For recording MALDI-TOF mass spectra MALDI-TOF Axima CFR spectrometer was used. Samples were ionized with the aid of a nitrogen laser (wavelength 337 nm, maximum power 6 MW). Gentisic acid (DHB) or α-cyano-4-hydroxycinnamic acid (HCCA) were used as matrices.

1.2. Bambusuril 6

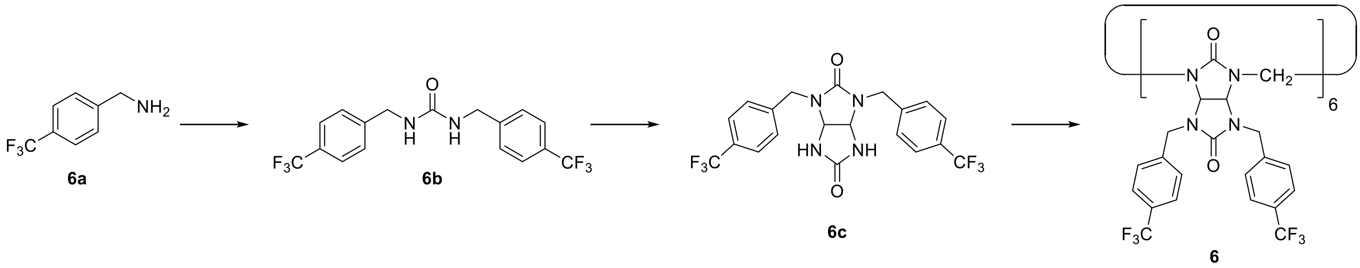

1.2.1. 1,3-Bis(4-(trifluoromethyl)benzyl)urea 6b

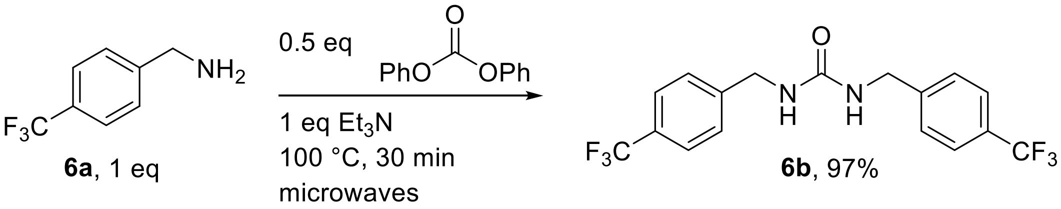

Finely ground diphenyl carbonate (1.607 g, 7.5 mmol, 0.5 eq), **6a** (2.627 g, 15 mmol, 1 eq) and triethylamine (2.1 mL, 15 mmol, 1 eq) were combined in a 35 mL microwave vessel and subjected to microwave irradiation at 100 °C for 30 min (300 W max. power). H_2O (50 mL) was added, and the mixture was sonicated and filtered. The product was washed with hot H_2O (40 mL), aqueous

HCl (10%, 20 mL), aqueous $NaHCO_3$ (saturated, 20 mL) and hot H_2O (30 mL). Drying in vacuum provided compound **6b** as a white solid (2.741 g, 97%).

^{1}H NMR (500 MHz, DMSO-*d*$_6$): δ = 7.67 (d, *J* = 8.0 Hz, 4H; Ar-H), 7.46 (d, *J* = 7.9 Hz, 4H; Ar-H), 6.68 (t, *J* = 6.2 Hz, 2H; NH), 4.32 (d, *J* = 6.1 Hz, 4H; CH_2). **^{13}C{^{1}H} NMR** (126 MHz, DMSO-*d*$_6$): δ = 158.1, 145.9 (q, *J* = 1 Hz), 127.5, 127.2 (q, *J* = 32 Hz), 125.5, 125.0 (q, *J* = 4 Hz), 42.6. **^{19}F NMR** (471 MHz, DMSO-*d*$_6$): δ = −60.77. **HRMS** (APCI): m/z calcd. for $C_{17}H_{14}F_6N_2O+H^+$: 377.1083 $[M+H]^+$; found: 377.1087.

1.2.2. 2,4-Bis(4-(trifluoromethyl)benzyl)glycoluril 6c

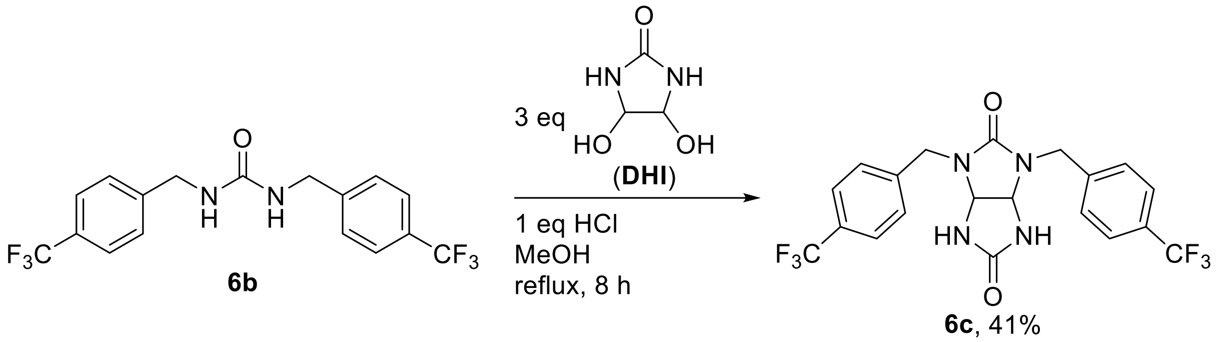

6b (1.882 g, 5 mmol, 1 eq) was suspended together with **DHI** (1.771 g, 15 mmol, 3 eq) in MeOH (25 mL) + aqueous HCl (35%, 0.444 mL, 5 mmol, 1 eq) and heated to reflux for 8 h. Then the reaction mixture was hot filtered through a Celite pad. The filtrate was loaded on Celite and purified by column chromatography (SiO_2, PhMe/MeCN gradient) to provide compound **6c** as a white solid (0.950 g, 41%).

^{1}H NMR (600 MHz, CD_3CN): δ = 7.68 (d, *J* = 7.7 Hz, 4H; Ar-H), 7.50 (d, *J* = 7.9 Hz, 4H; Ar-H), 5.84 (s, 2H; NH), 5.19 (s, 2H; CH), 4.66 (d, *J* = 16.2 Hz, 2H; CH_2), 4.35 (d, *J* = 16.2 Hz, 2H; CH_2). **^{13}C{^{1}H} NMR** (151 MHz, CD_3CN): δ = 161.6, 159.0, 143.5, 129.8 (q, *J* = 32 Hz), 129.3, 126.4 (q, *J* = 4 Hz), 125.5 (q, *J* = 271 Hz), 67.5, 45.8. **^{19}F NMR** (565 MHz, CD_3CN): δ = −62.83. **MS** (APCI): m/z calcd. for $C_{20}H_{16}F_6N_4O_2+H^+$: 459.1250 $[M+H]^+$; found: 459.1253.

1.2.3. Dodekakis(4-(trifluoromethyl)benzyl)bambus[6]uril 6

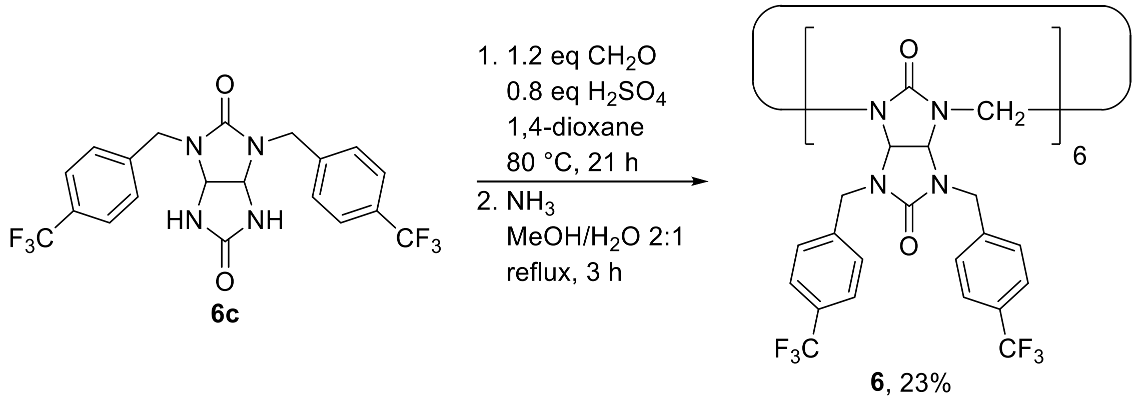

6c (458 mg, 1 mmol, 1 eq), paraformaldehyde (36 mg, 1.2 mmol, 1.2 eq), were suspended in 1,4-dioxane (2 mL) and after the addition of H_2SO_4 (42.9 µL, 0.8 mmol, 0.8 eq) the solids dissolved. The reaction mixture was heated to 80 °C for 21 h. The brown solid was collected by filtration and washed with dioxane (2×1 mL) and Et_2O (3×2 mL). The solid was suspended in a mixture of

MeOH (10 mL), mili-Q water (4 mL) and aqueous NH_3 (24%, 1 mL) and heated to reflux for 3 h. The solid was collected by centrifugation and washed with MeOH (5 mL), mili-Q water (10 mL), MeOH (5 mL) and Et_2O (15 mL). Each washing step was done by dispersing the solid in a liquid, sonication and centrifugation, then the mother liquor was discarded. Drying in vacuum provided compound **6** as a white solid (107 mg, 23%). (Note: complete removal of the anion was not successful, therefore **6** was characterised in the presence of excess Me_4NI as an I^- complex.)

^{1}H NMR (400 MHz, CD_3CN): δ = 7.59 (d, *J* = 8.0 Hz, 24H; Ar-H), 7.45 (d, *J* = 8.0 Hz, 24H; Ar-H), 5.81 (s, 12H; CH), 4.91 (d, *J* = 16.8 Hz, 12H; CH_2), 4.68 (d, *J* = 16.9 Hz, 12H; CH_2), 4.10 (s, 12H; CH_2). **^{13}C{^{1}H} NMR** (101 MHz, CD_3CN): δ = 160.7, 160.0, 145.1, 129.6 (q, *J* = 32 Hz), 127.9, 126.3, 125.4 (q, *J* = 271 Hz), 70.9, 48.5, 48.3. **^{19}F NMR** (565 MHz, CD_3CN): δ = −62.85. **MS** (MALDI): m/z calcd. for $C_{126}H_{96}F_{36}N_{24}O_{12}$+$Na^+$: 2843.696 $[M+Na]^+$; found: 2843.732.

1.3. Bambusuril 8

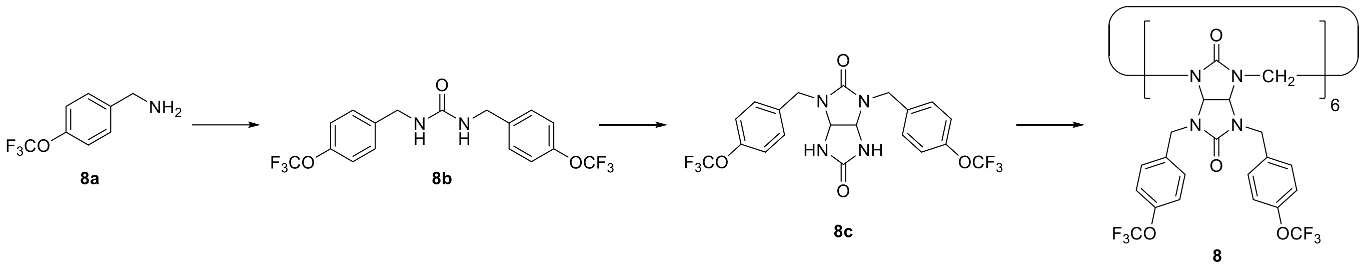

1.3.1. 1,3-Bis(4-(trifluoromethoxy)benzyl)urea 8b

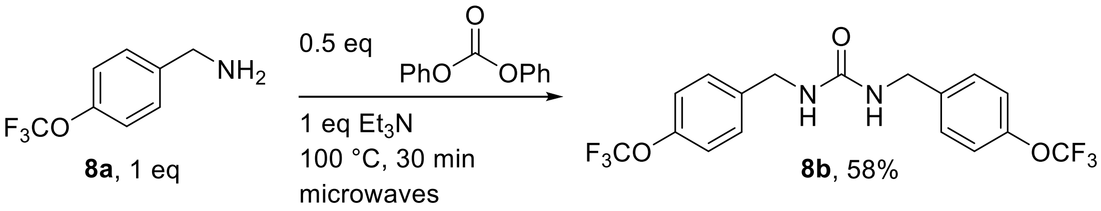

Finely ground diphenyl carbonate (5.356 g, 25 mmol, 0.5 eq), **8a** (9.558 g, 50 mmol, 1 eq) and triethylamine (6.9 mL, 50 mmol, 1 eq) were combined in a 35 mL microwave vessel and subjected to microwave irradiation at 100 °C for 30 min (300 W max. power). MeOH (40 mL) was added, and the mixture was sonicated and filtered. The product was washed with MeCN (2×30 mL), MeOH (50 mL) and Et_2O (50 mL). Mother liquors were concentrated and after the addition of cold Et_2O (100 mL), additional product emerged, which was filtered together with the first fraction, and both were washed with cold MeCN (20 mL) and Et_2O (2×30 mL). Drying in vacuum provided compound **8b** as a white solid (5.972 g, 58%).

^{1}H NMR (500 MHz, DMSO-*d*$_6$): δ = 7.36 (d, *J* = 8.7 Hz, 4H; Ar-H), 7.30 (d, *J* = 7.8 Hz, 4H; Ar-H), 6.56 (t, *J* = 6.1 Hz, 2H; NH), 4.25 (d, *J* = 6.1 Hz, 4H; CH_2). **^{13}C{^{1}H} NMR** (126 MHz, DMSO-*d*$_6$): δ = 168.0, 147.0 (q, *J* = 2 Hz), 140.5, 128.7, 120.8, 120.1 (q, *J* = 256 Hz), 42.3. **^{19}F NMR** (471 MHz, DMSO-*d*$_6$): δ = −56.86. **HRMS** (APCI): m/z calcd. for $C_{17}H_{14}F_6N_2O_3$+H^+: 409.0981 $[M+H]^+$; found: 409.0985.

1.3.2. 2,4-Bis(4-(trifluoromethoxy)benzyl)glycoluril 8c

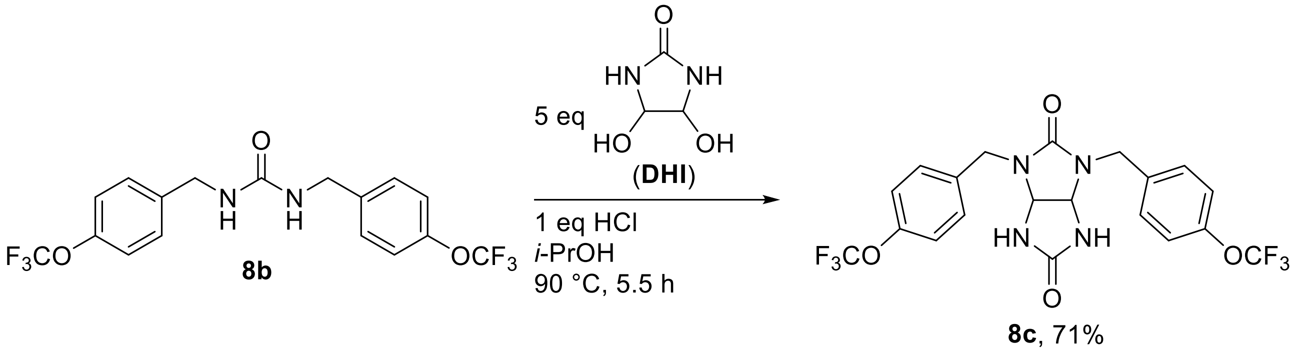

8b (4.083 g, 10 mmol, 1 eq) was suspended together with 1 eq of **DHI** (1.181 g, 10 mmol) (total **DHI** amount was 5 eq (7.086 g, 50 mmol)) in *i*-PrOH (100 mL) + aqueous HCl (35%, 0.887 mL, 10 mmol, 1 eq) and heated to 90 °C. The rest of **DHI** was added in 8 portions (0.5 eq per addition) in 30 min intervals from the start. 1.5 h after the last **DHI** was added, the reaction mixture was hot filtered through a Celite pad and the filter cake was washed with hot *i*-PrOH (2×30 mL). The filtrates were concentrated, and water (50 mL) was added. The suspension was heated to a boiling point and filtered. The solid was washed with boiling water (2×50 mL) and dried in vacuum (4823 mg). It was loaded on SiO_2 and purified by column chromatography (SiO_2, PhMe/MeCN gradient) to provide compound **8c** as a white solid (3.490 g, 71%).

^{1}H NMR (500 MHz, DMSO-d_6): δ = 7.61 (s, 2H; NH), 7.40 (d, J = 8.7 Hz, 4H; Ar-H), 7.34 (d, J = 8.3 Hz, 4H; Ar-H), 5.13 (s, 2H; CH), 4.60 (d, J = 15.8 Hz, 2H; CH_2), 4.14 (d, J = 15.8 Hz, 2H; CH_2). **^{13}C{^{1}H} NMR** (126 MHz, DMSO-d_6): δ = 160.8, 157.3, 147.5 (q, J = 2 Hz), 137.0, 129.5, 121.1, 120.1 (q, J = 256 Hz), 65.5, 43.5. **^{19}F NMR** (471 MHz, DMSO-d_6): δ = −56.80. **HRMS** (APCI): m/z calcd. for $C_{20}H_{16}F_6N_4O_4+H^+$: 491.1149 $[M+H]^+$; found: 491.1151.

1.3.3. Dodekakis(4-(trifluoromethoxy)benzyl)bambus[6]uril 8

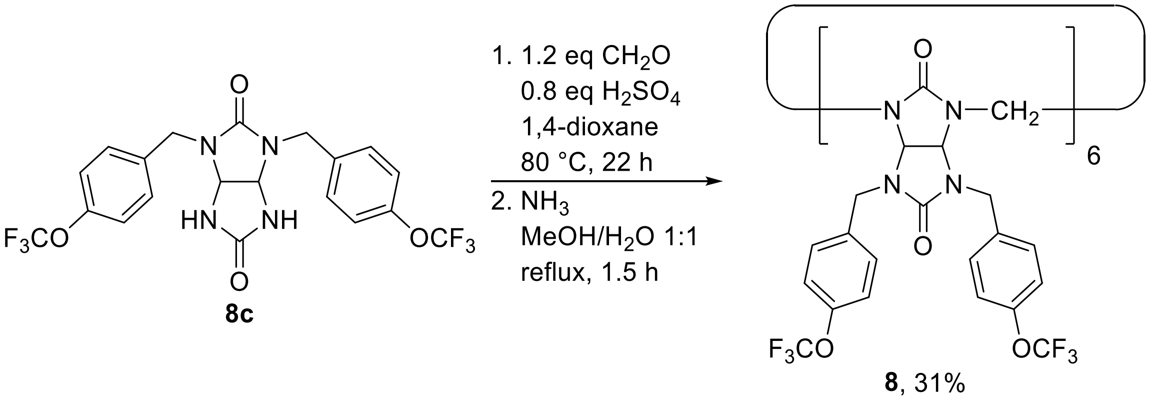

8c (981 mg, 2 mmol, 1 eq), paraformaldehyde (73 mg, 2.4 mmol, 1.2 eq), were suspended in 1,4-dioxane (4 mL) and after the addition of H_2SO_4 (85.7 µL, 1.6 mmol, 0.8 eq) the solids dissolved. The reaction mixture was heated to 80 °C for 22 h. The solid was collected by filtration and washed with dioxane (2×2 mL) and Et_2O (2×5 mL). The solid was suspended in a mixture of MeOH (10 mL), mili-Q water (8 mL) and aqueous NH_3 (24%, 2 mL) and heated to reflux for 1.5 h. The solid was then filtrated and washed with mili-Q water (2×10 mL) and MeOH (2×10 mL). Drying in vacuum provided compound **8** as a white solid (312 mg, 31%).

¹H NMR (400 MHz, CD_3CN): δ = 7.38 (d, *J* = 8.7 Hz, 24H; Ar-H), 7.22 (d, *J* = 8.5 Hz, 24H; Ar-H), 5.26 (s, 12H; CH), 4.81 (d, *J* = 16.5 Hz, 12H; CH_2), 4.60 (d, *J* = 16.5 Hz, 12H; CH_2), 4.12 (s, 12H; CH_2). **¹³C{¹H} NMR** (101 MHz, CD_3CN): δ = 160.9, 159.6, 149.2, 138.7, 129.7, 122.0, 121.5 (q, *J* = 255 Hz), 71.0, 49.0, 48.4 (Note: low-intensity peaks corresponding to the quartet of the carbon atom in the -CF_3 are poorly resolved from the noise). **¹⁹F NMR** (565 MHz, CD_3CN): δ = −58.55. **MS** (ESI): m/z calcd. for $C_{126}H_{96}F_{36}N_{24}O_{24}+NH_4^+$: 3031.6827 $[M+NH_4]^+$; found: 3031.6742.

1.4. Bambusuril 11

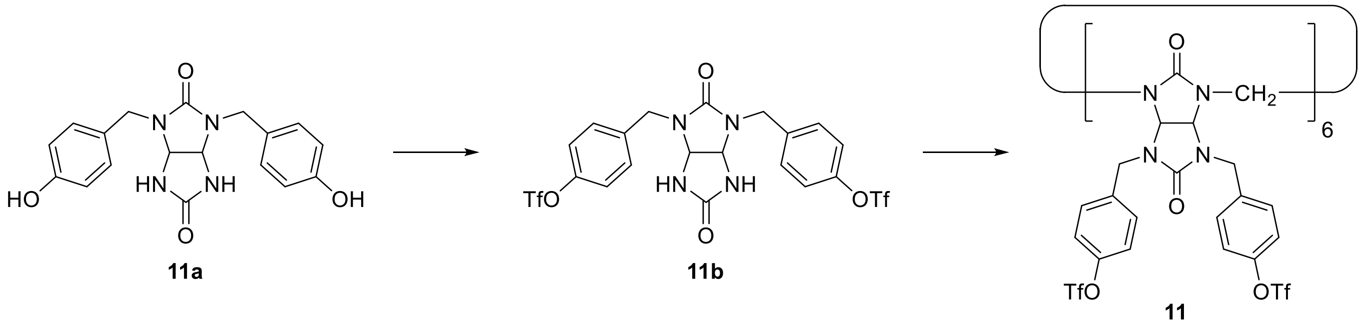

1.4.1. 2,4-Bis(4-(triflyloxy)benzyl)glycoluril 11b

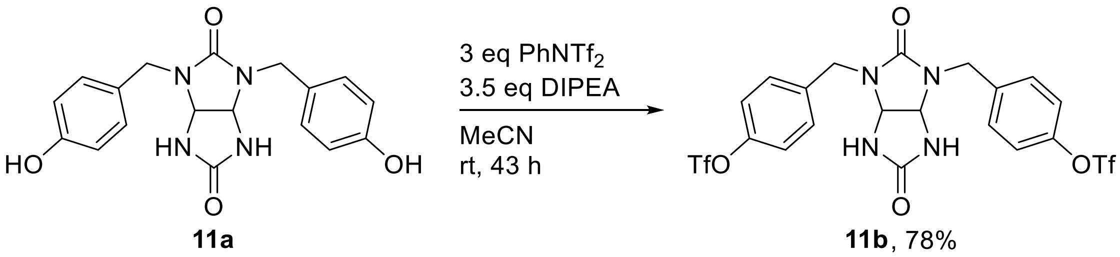

11a (709 mg, 2 mmol, 1 eq) and $PhNTf_2$ (2143 mg, 6 mmol, 3 eq) were flushed with argon. Dry MeCN (20 mL) and DIPEA (1219 µL, 7 mmol, 3.5 eq) were added and the reaction mixture was stirred at ambient temperature for 43 h under an argon atmosphere. The brown solution was concentrated, then diluted with EtOAc (40 mL). The resulting solution was washed with aqueous HCl (10%, 30 mL), water (40 mL) and brine (50 mL). The organic phase was dried over $MgSO_4$ and filtered. The filtrate was loaded on Celite and purified by flash chromatography (SiO_2, EtOAc in cyclohexane 50 to 100%) to provide compound **11b** as a beige solid (963 mg, 78%).

¹H NMR (500 MHz, acetone-*d*₆): δ = 7.56 (d, *J* = 8.7 Hz, 4H; Ar-H), 7.42 (d, *J* = 8.8 Hz, 4H; Ar-H), 6.94 (s, 2H; NH), 5.40 (s, 2H; CH), 4.78 (d, *J* = 15.9 Hz, 2H; CH_2), 4.35 (d, *J* = 15.9 Hz, 2H; CH_2). **¹³C{¹H} NMR** (126 MHz, acetone-d_6): δ = 161.7, 158.7, 149.8, 139.9, 130.9, 122.4, 119.7 (q, *J* = 320 Hz), 67.2, 45.0. **¹⁹F NMR** (471 MHz, acetone-d_6): δ = −74.24. **MS** (APCI): m/z calcd. for $C_{20}H_{16}F_6N_4O_8S_2+H^+$: 619.0387 $[M+H]^+$; found: 619.0390.

1.4.2. Dodekakis(4-(triflyloxy)benzyl)bambus[6]uril 11

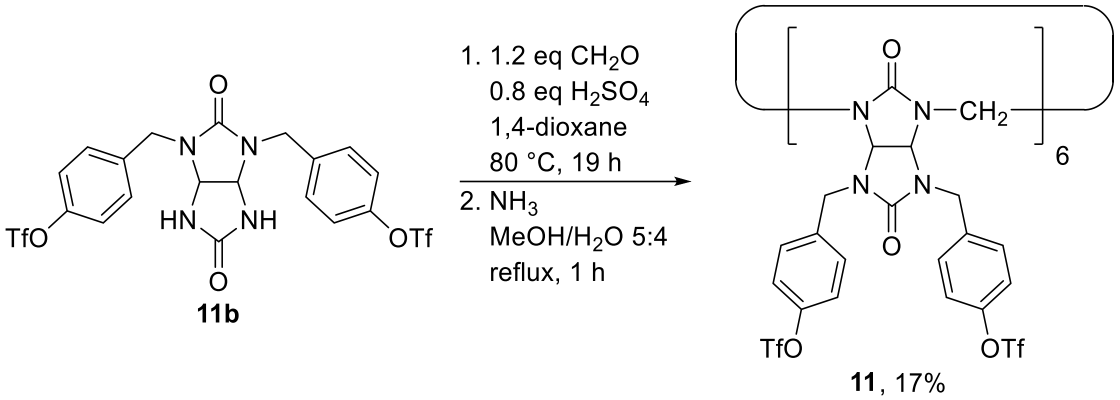

11b (618 mg, 1 mmol, 1 eq), paraformaldehyde (36 mg, 2.4 mmol, 1.2 eq), were suspended in 1,4-dioxane (2 mL) and after the addition of H_2SO_4 (42.9 µL, 0.8 mmol, 0.8 eq) the solids dissolved giving a yellow solution. The reaction mixture was heated to 80 °C for 19 h. The solid was collected by filtration and washed with 1,4-dioxane (2×0.5 mL) and Et_2O (3×3 mL). The solid was suspended in a mixture of MeOH (5 mL), mili-Q water (3 mL) and aqueous NH_3 (24%, 1 mL) and heated to reflux for 1 h. The solid was collected by centrifugation and washed with mili-Q water (2×5 mL), MeOH (5 mL) and Et_2O (2×5 mL). Each washing step was done by dispersing the solid in a liquid, sonication and centrifugation, then the mother liquor was discarded. Drying in vacuum provided compound **11** as a white solid (109 mg, 17%).

^{1}H NMR (400 MHz, CD_3CN): δ = 7.45 (d, *J* = 8.8 Hz, 24H; Ar-H), 7.32 (d, *J* = 8.8 Hz, 24H; Ar-H), 5.27 (s, 12H; CH), 4.85 (d, *J* = 16.7 Hz, 12H; CH_2), 4.63 (d, *J* = 16.7 Hz, 12H; CH_2), 4.10 (s, 12H; CH_2). **^{13}C{^{1}H} NMR** (101 MHz, CD_3CN): δ = 161.0, 159.6, 149.7, 140.5, 130.0, 122.6, 71.2, 48.9, 48.5 (Note: some peaks corresponding to the quartet of the carbon atom in the -CF_3 overlap with other signals, therefore it is not listed here). **^{19}F NMR** (565 MHz, CD_3CN): δ = −73.81. **MS** (MALDI): m/z calcd. for $C_{126}H_{96}F_{36}N_{24}O_{48}S_{12}$+$I^-$: 3908.096 $[M+I]^-$; found: 3907.750.

2. Characterization

2.1. 6b

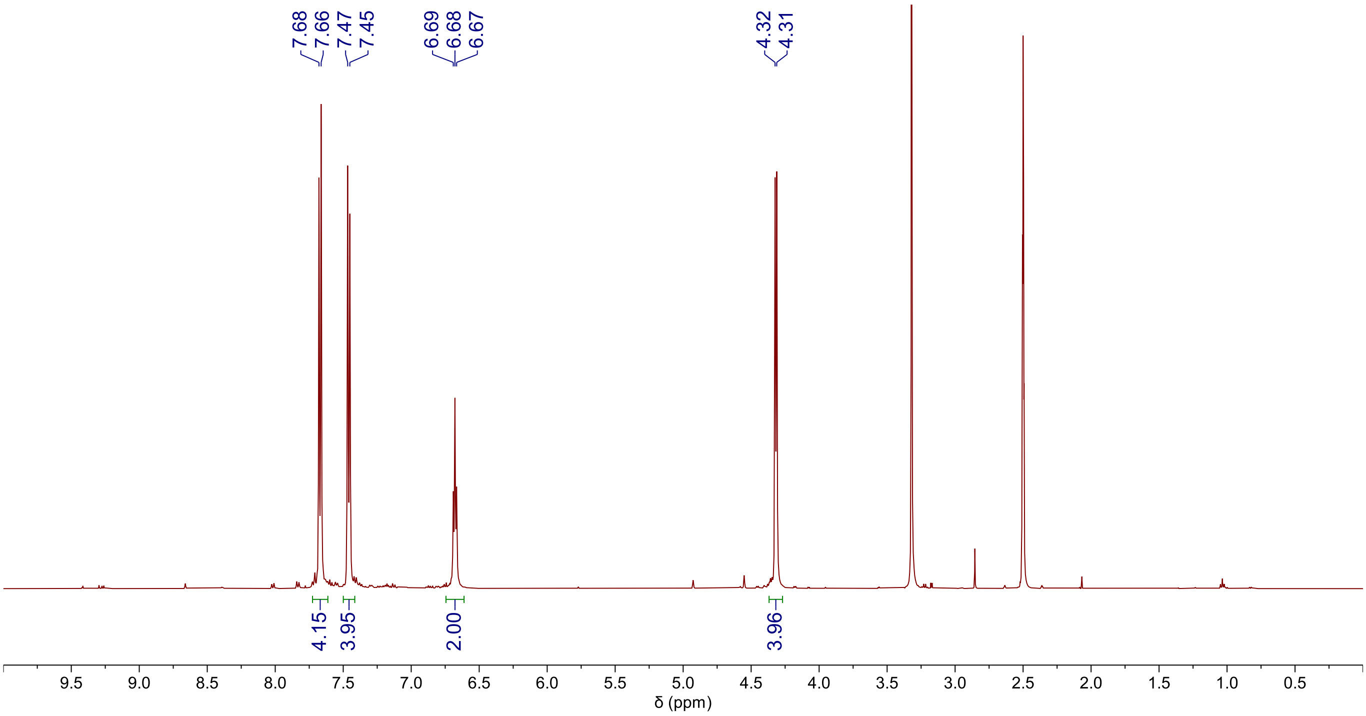

Figure S1: ^{1}H NMR spectrum of **6b** (500 MHz, DMSO-d_6).

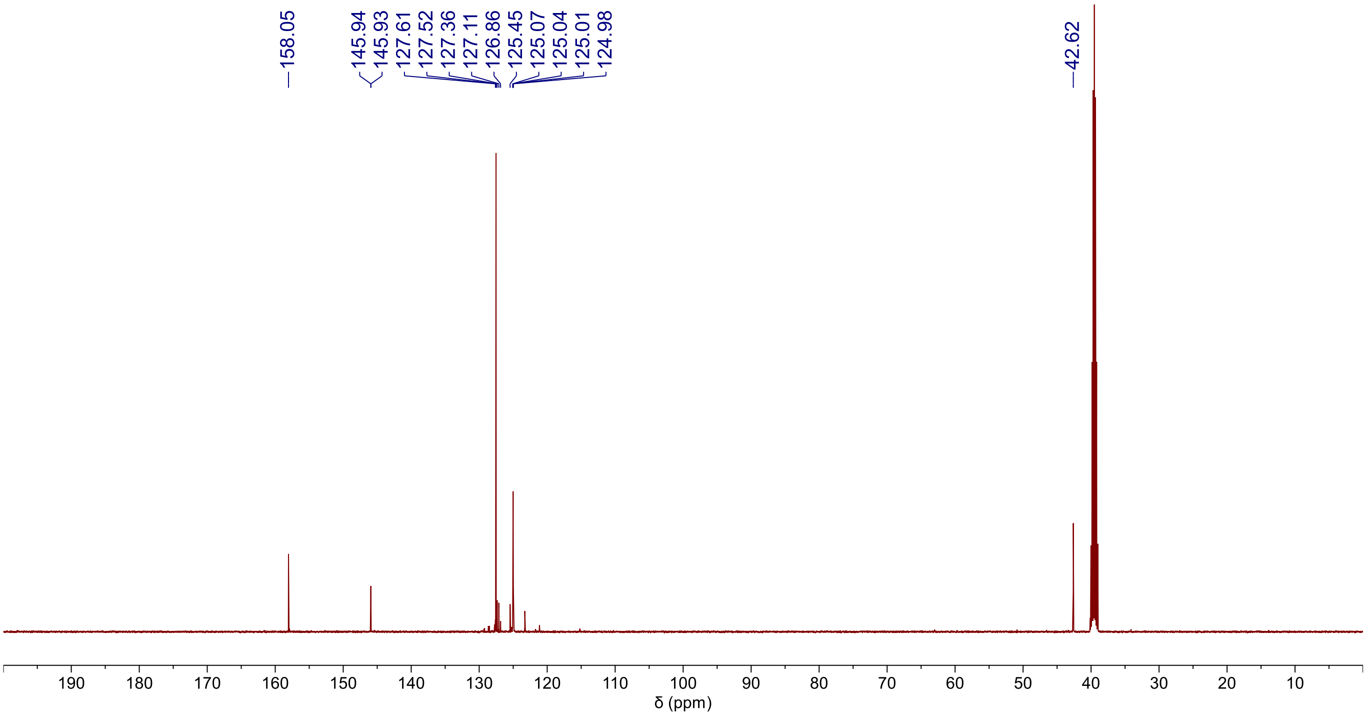

Figure S2: $^{13}C\{^{1}H\}$ NMR spectrum of **6b** (126 MHz, DMSO-d_6).

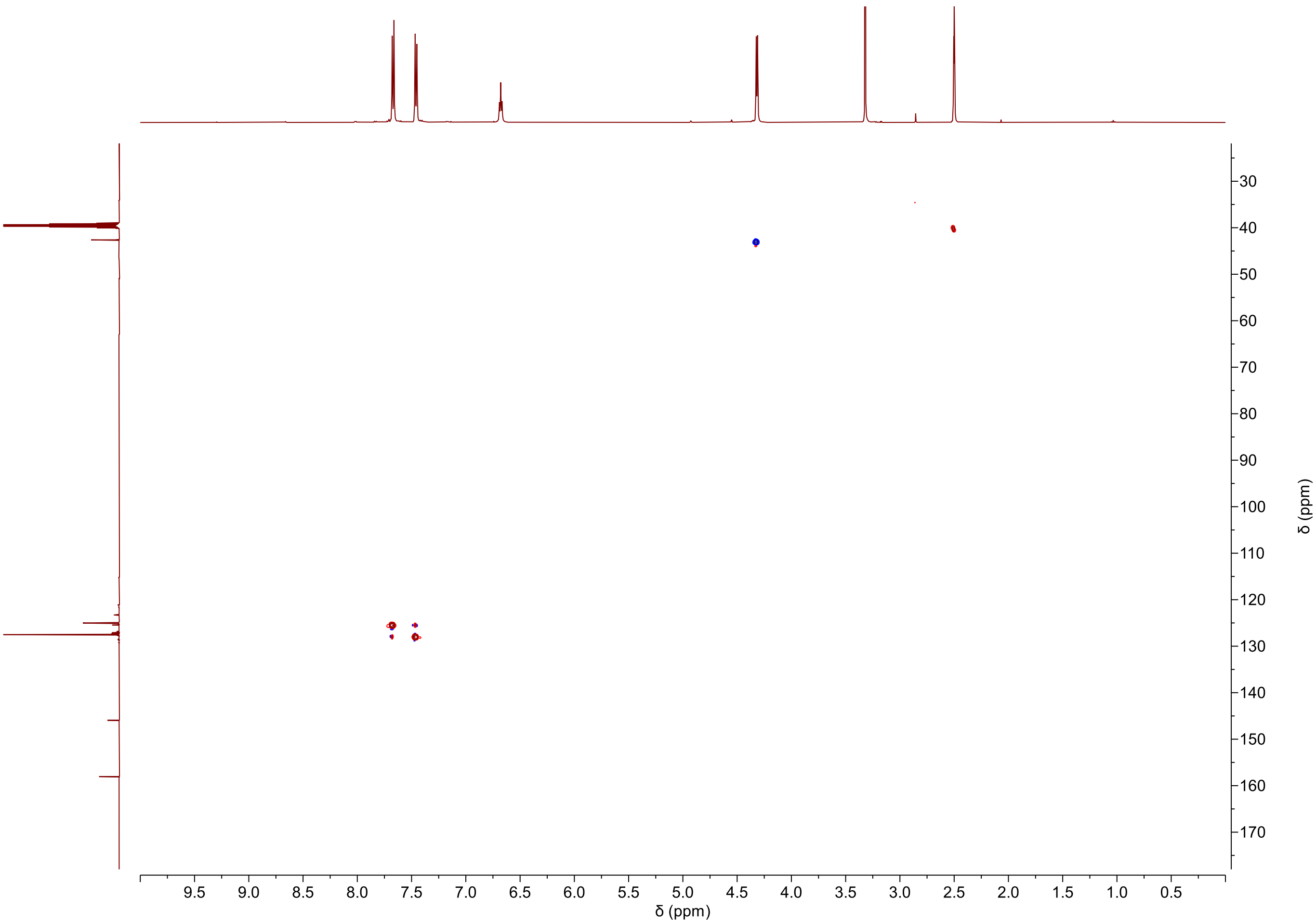

Figure S3: ^{1}H-^{13}C HSQC NMR spectrum of **6b** (500 MHz, 126 MHz, DMSO-d_6).

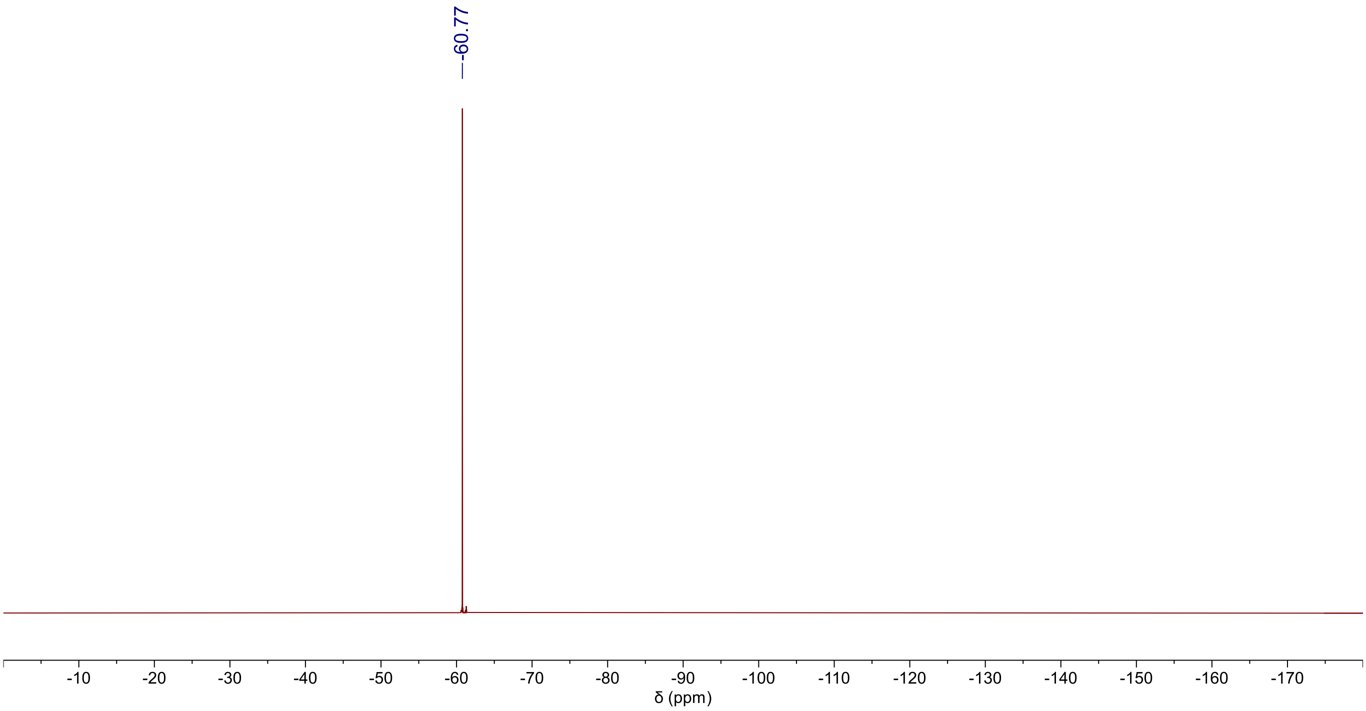

Figure S4: ^{19}F NMR spectrum of **6b** (471 MHz, DMSO-d_6).

2.2. 6c

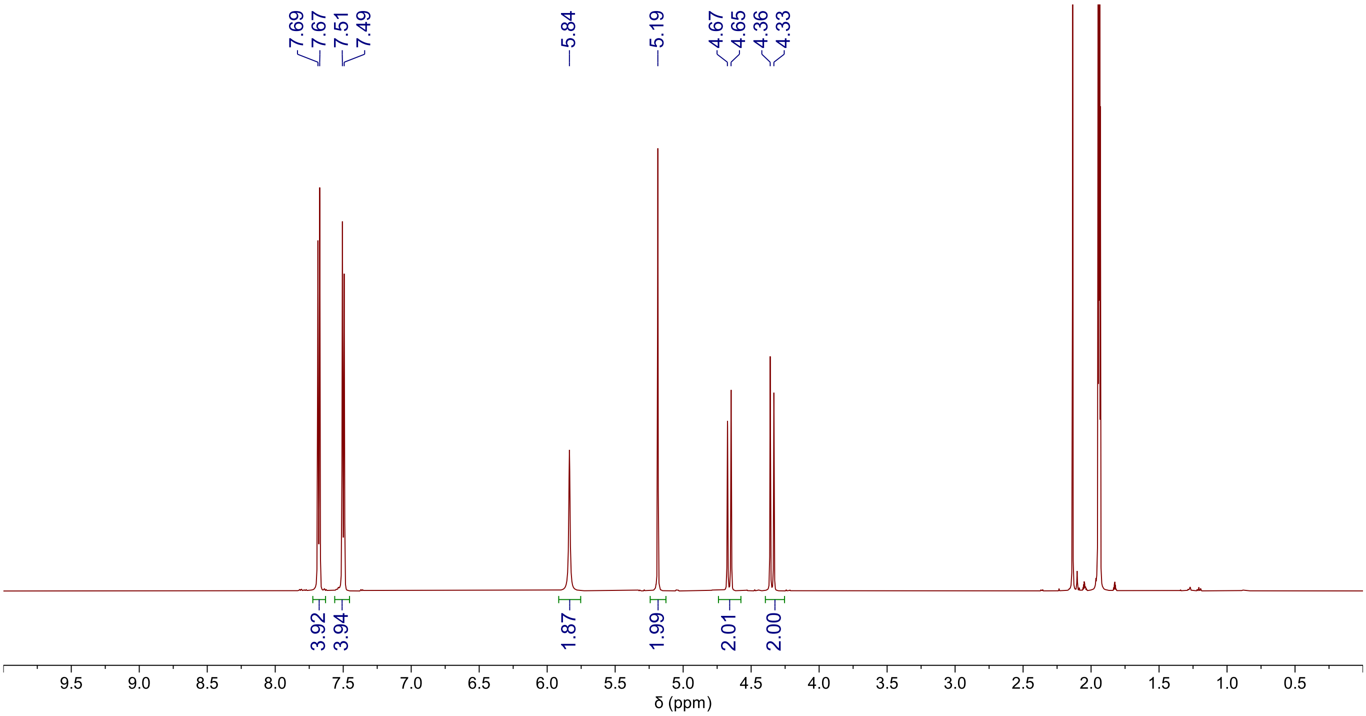

Figure S5: ^{1}H NMR spectrum of **6c** (500 MHz, CD_3CN).

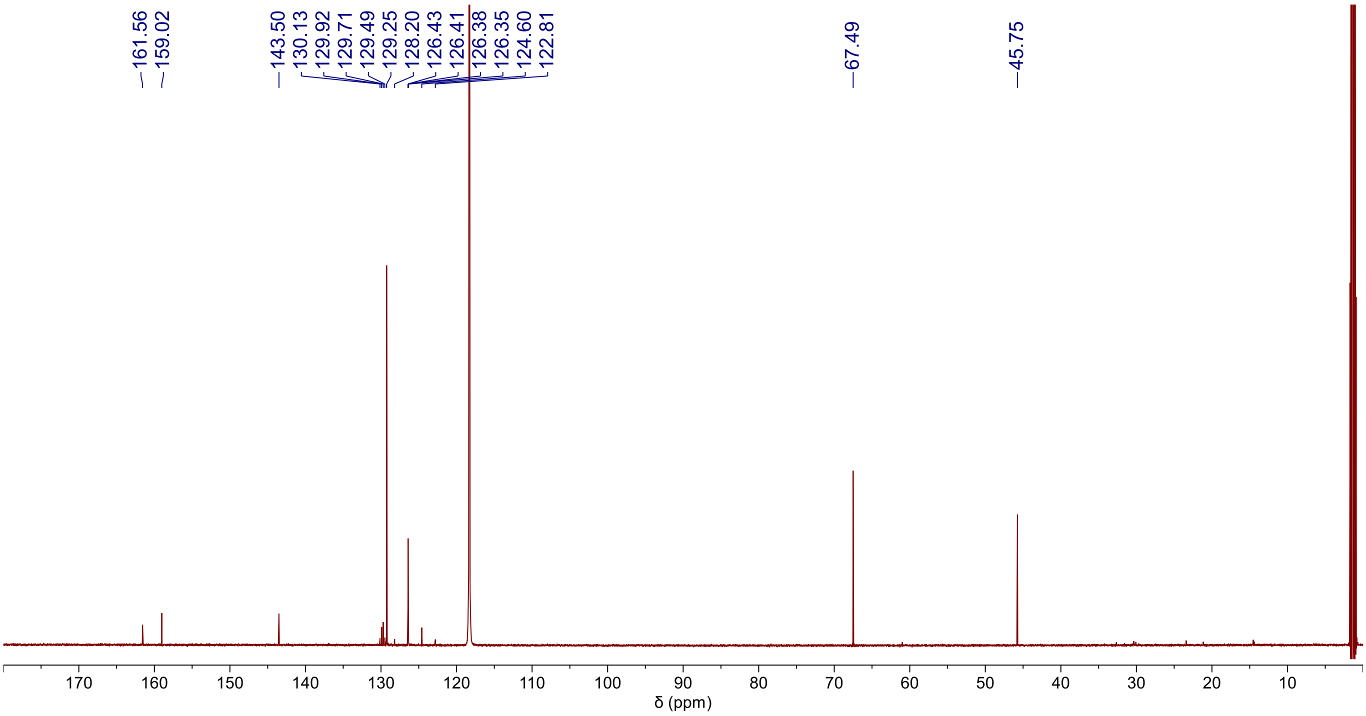

Figure S6: $^{13}C\{^{1}H\}$ NMR spectrum of **6c** (151 MHz, CD_3CN).

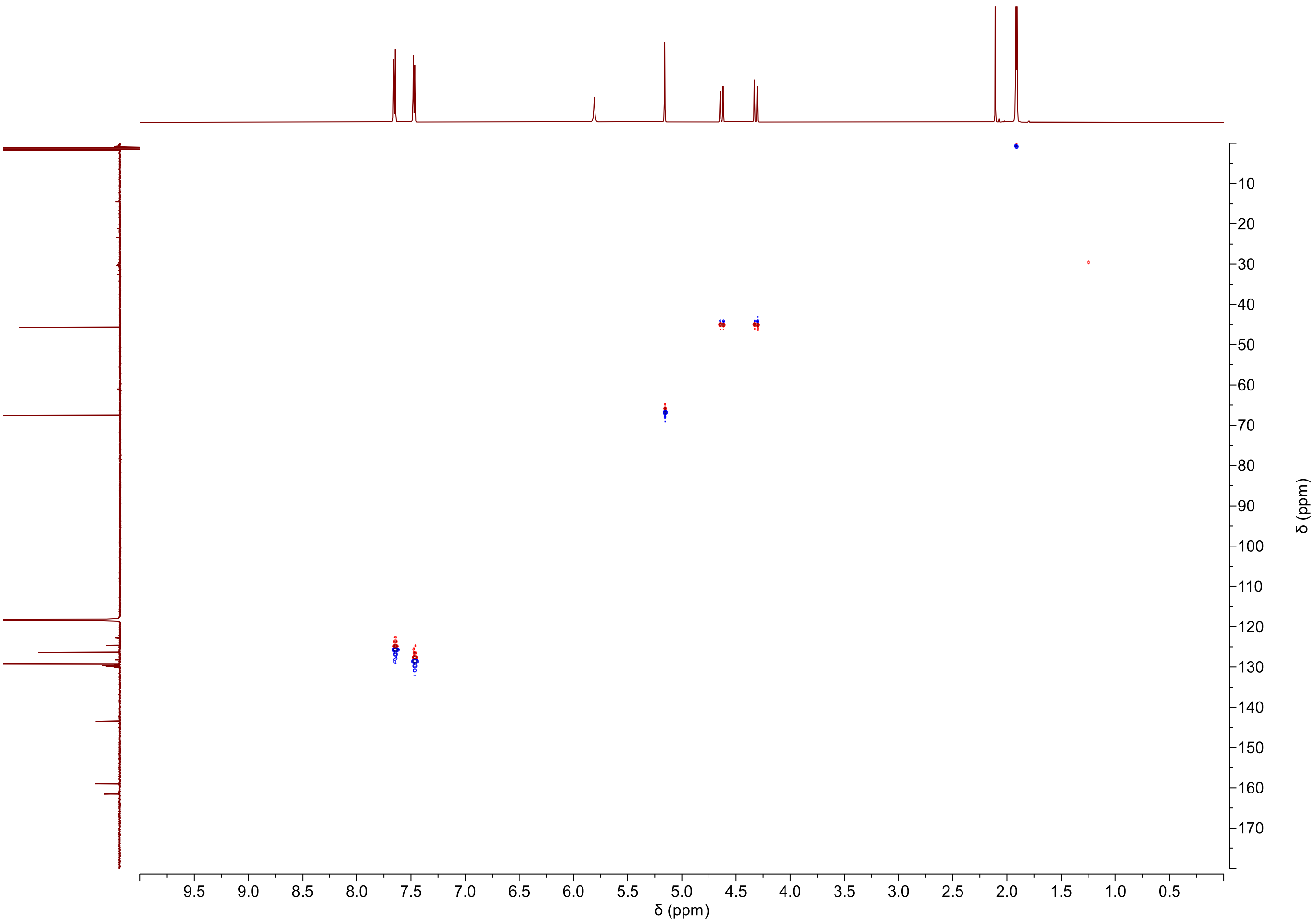

Figure S7: ^{1}H-^{13}C HSQC NMR spectrum of **6c** (600 MHz, 151 MHz, CD_3CN).

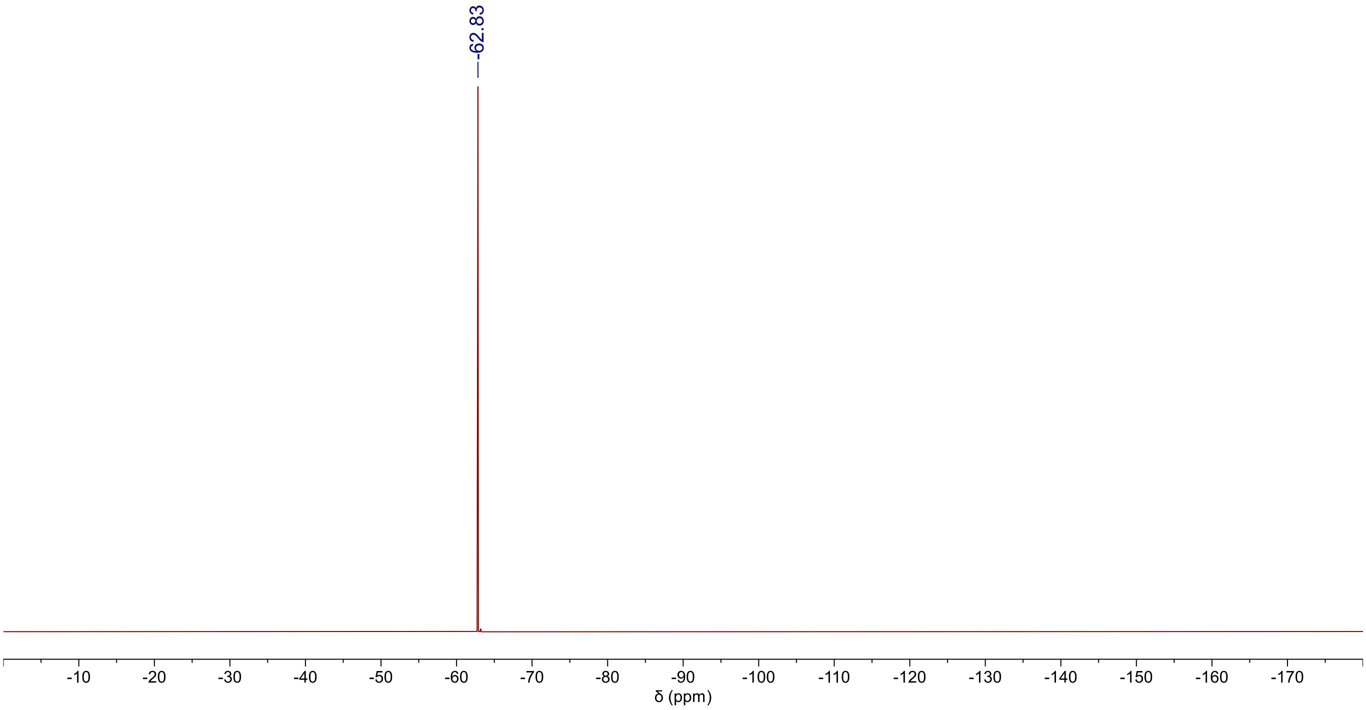

Figure S8: ^{19}F NMR spectrum of **6c** (565 MHz, CD_3CN).

2.3. 6

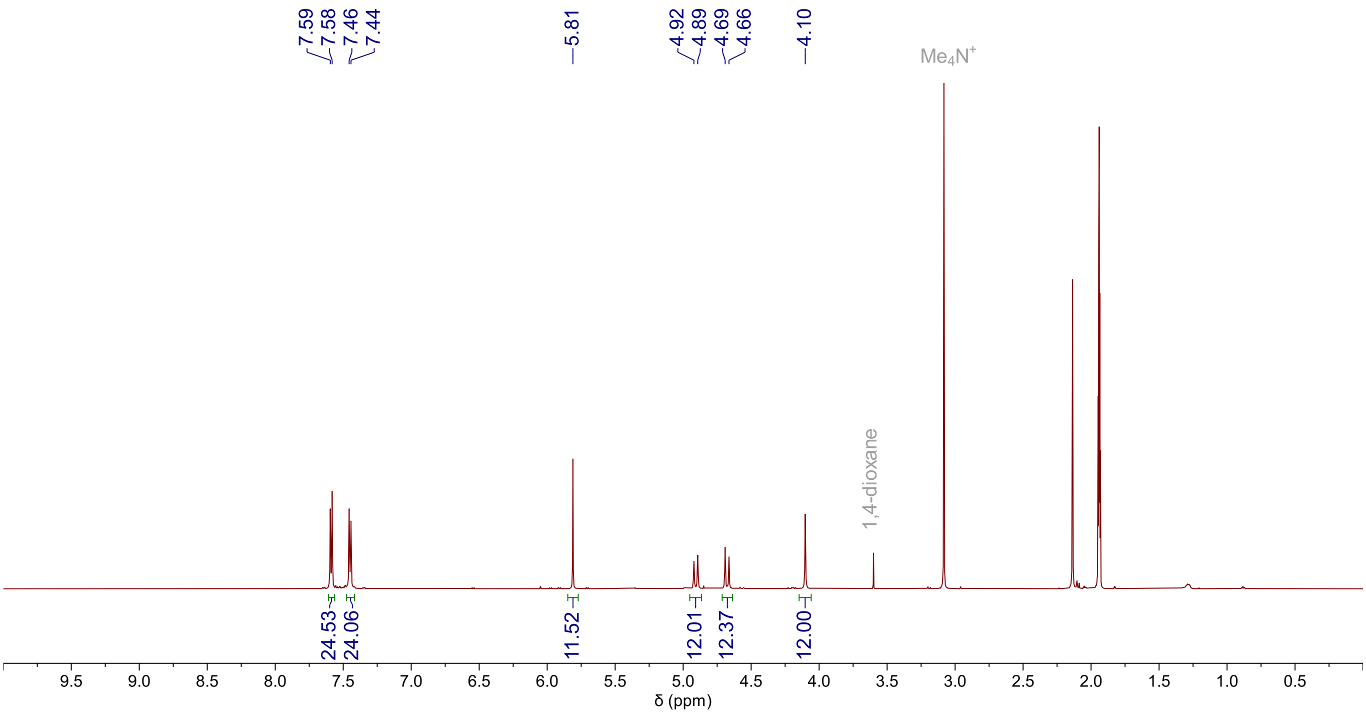

Figure S9: ^{1}H NMR spectrum of **6** in the presence of excess Me_4NI (600 MHz, CD_3CN).

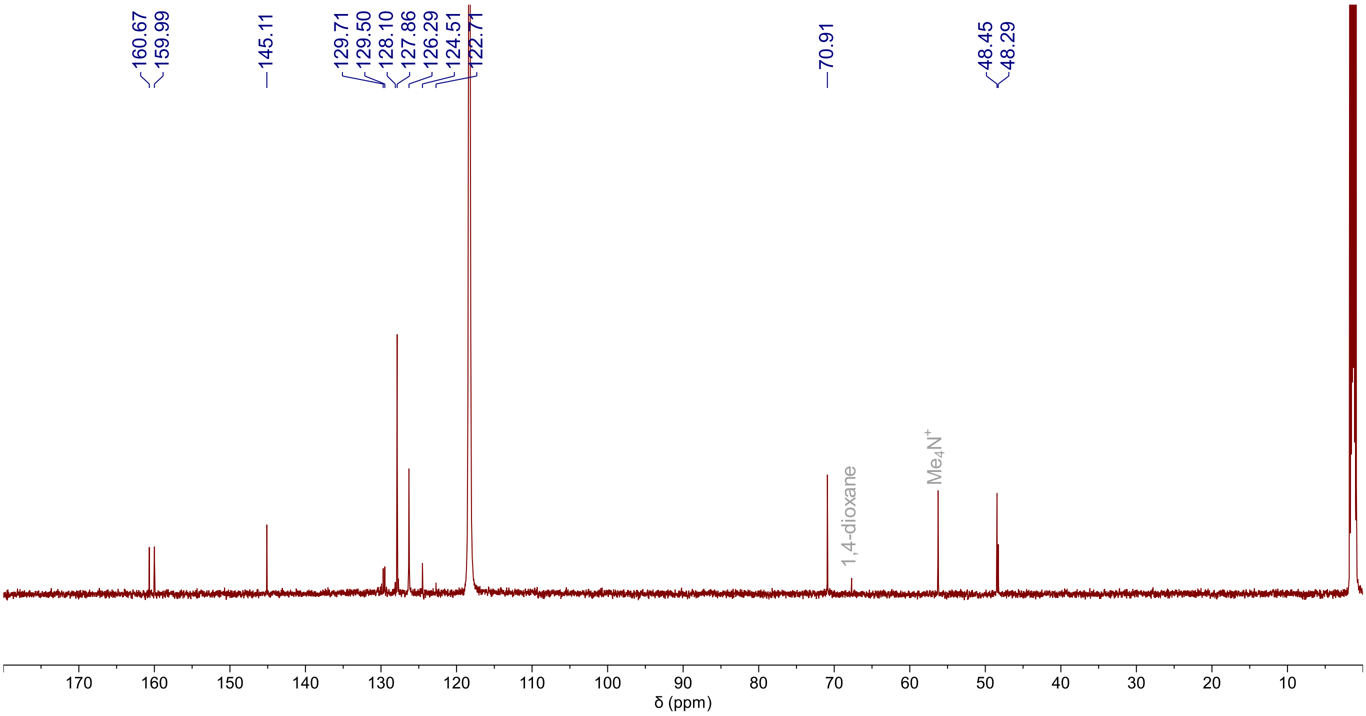

Figure S10: $^{13}C\{^{1}H\}$ NMR spectrum of **6** in the presence of excess Me_4NI (151 MHz, CD_3CN).

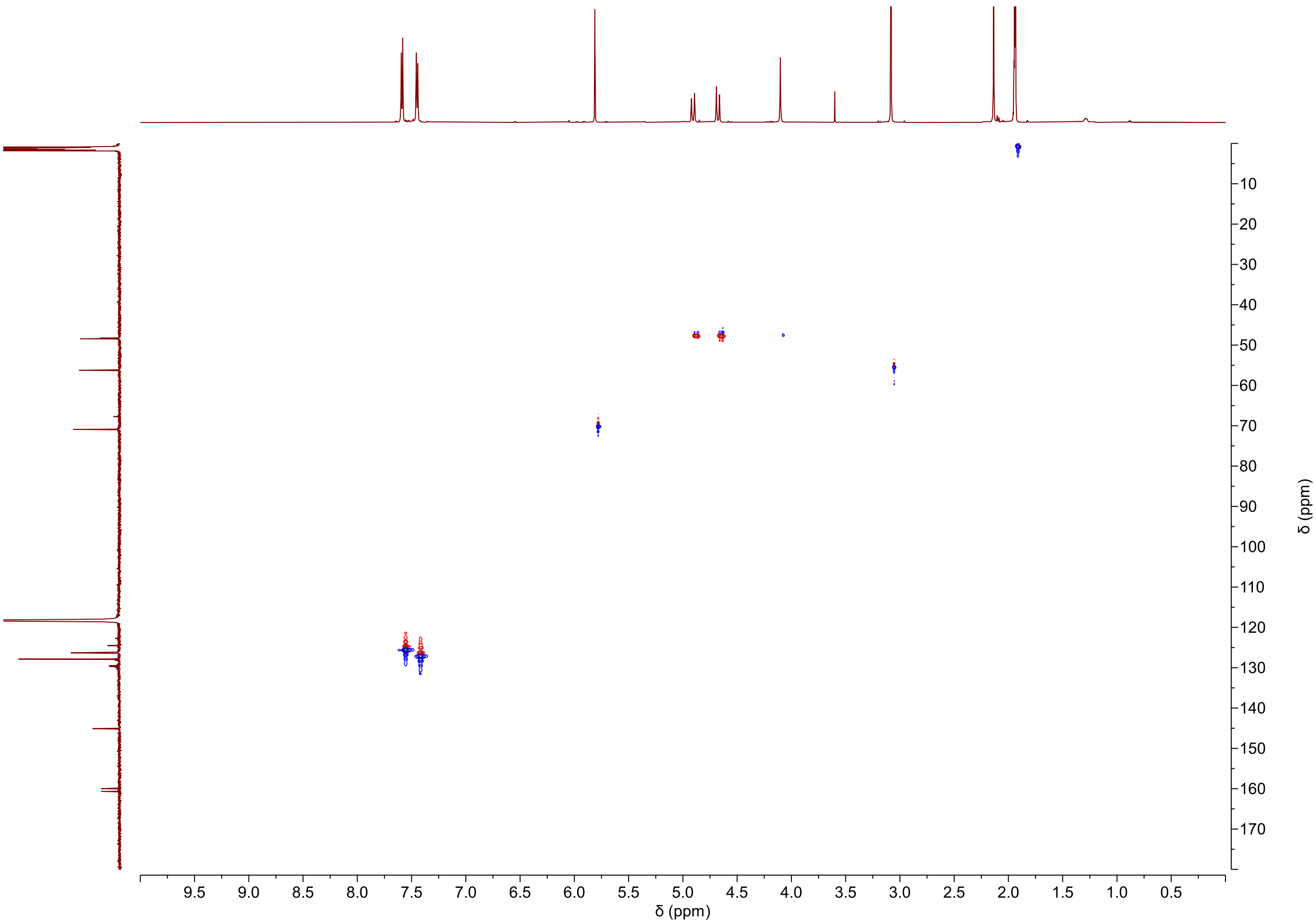

Figure S11: ^{1}H-^{13}C HSQC NMR spectrum of **6** in the presence of excess Me_4NI (600 MHz, 151 MHz, CD_3CN).

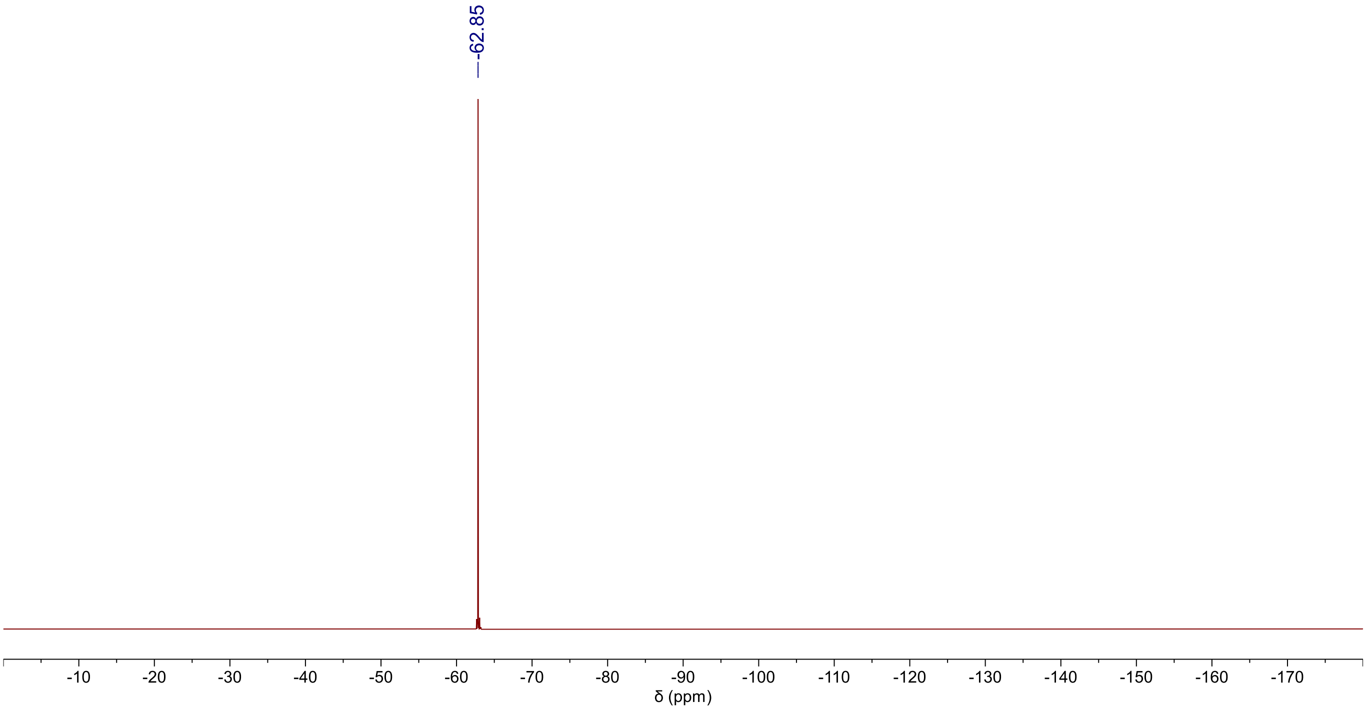

Figure S12: ^{19}F NMR spectrum of **6** in the presence of excess Me_4NI (565 MHz, CD_3CN).

2.4. 8b

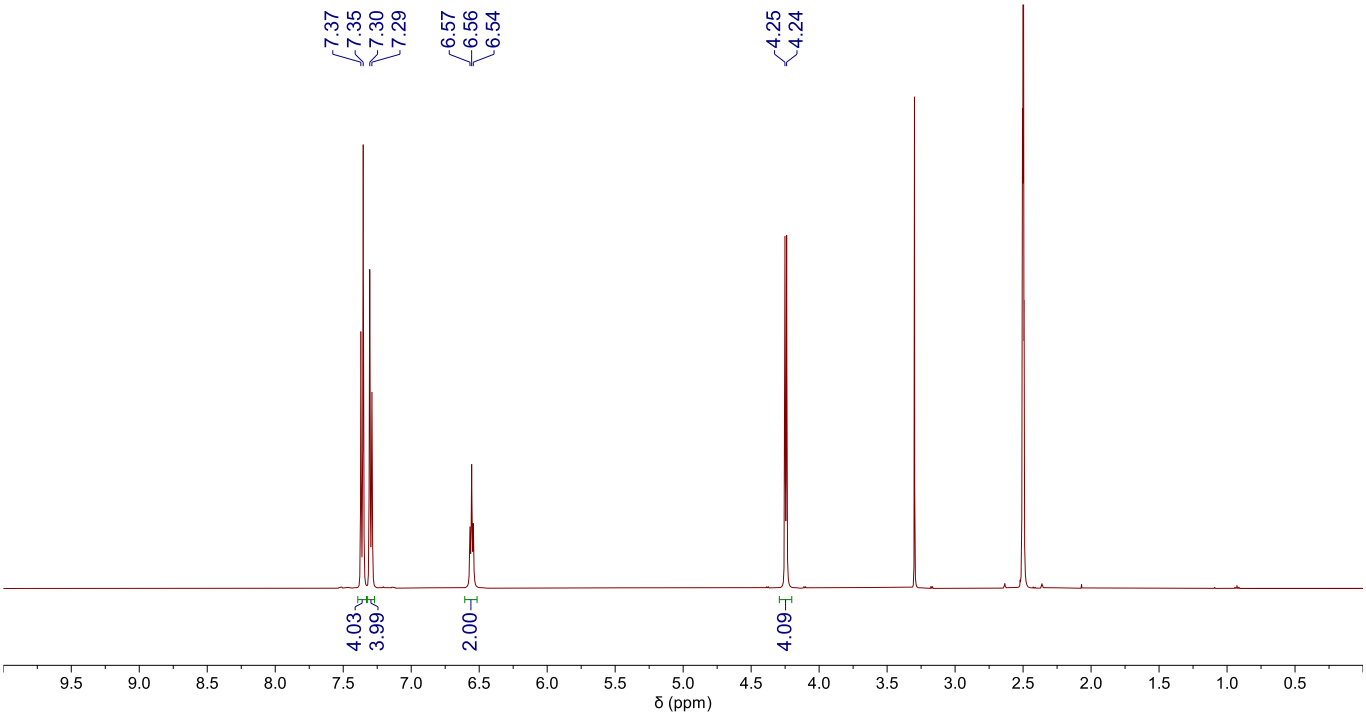

Figure S13: ^{1}H NMR spectrum of **8b** (500 MHz, DMSO-d_6).

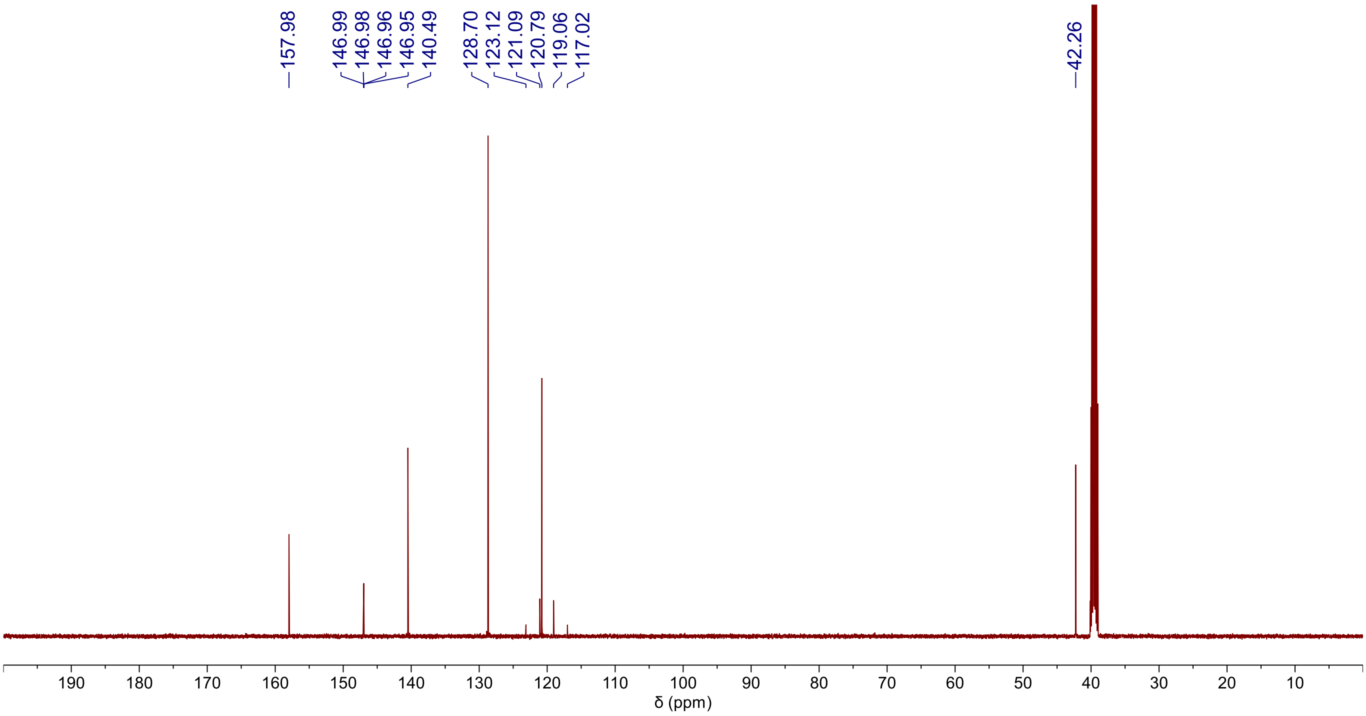

Figure S14: $^{13}C\{^{1}H\}$ NMR spectrum of **8b** (126 MHz, DMSO-d_6).

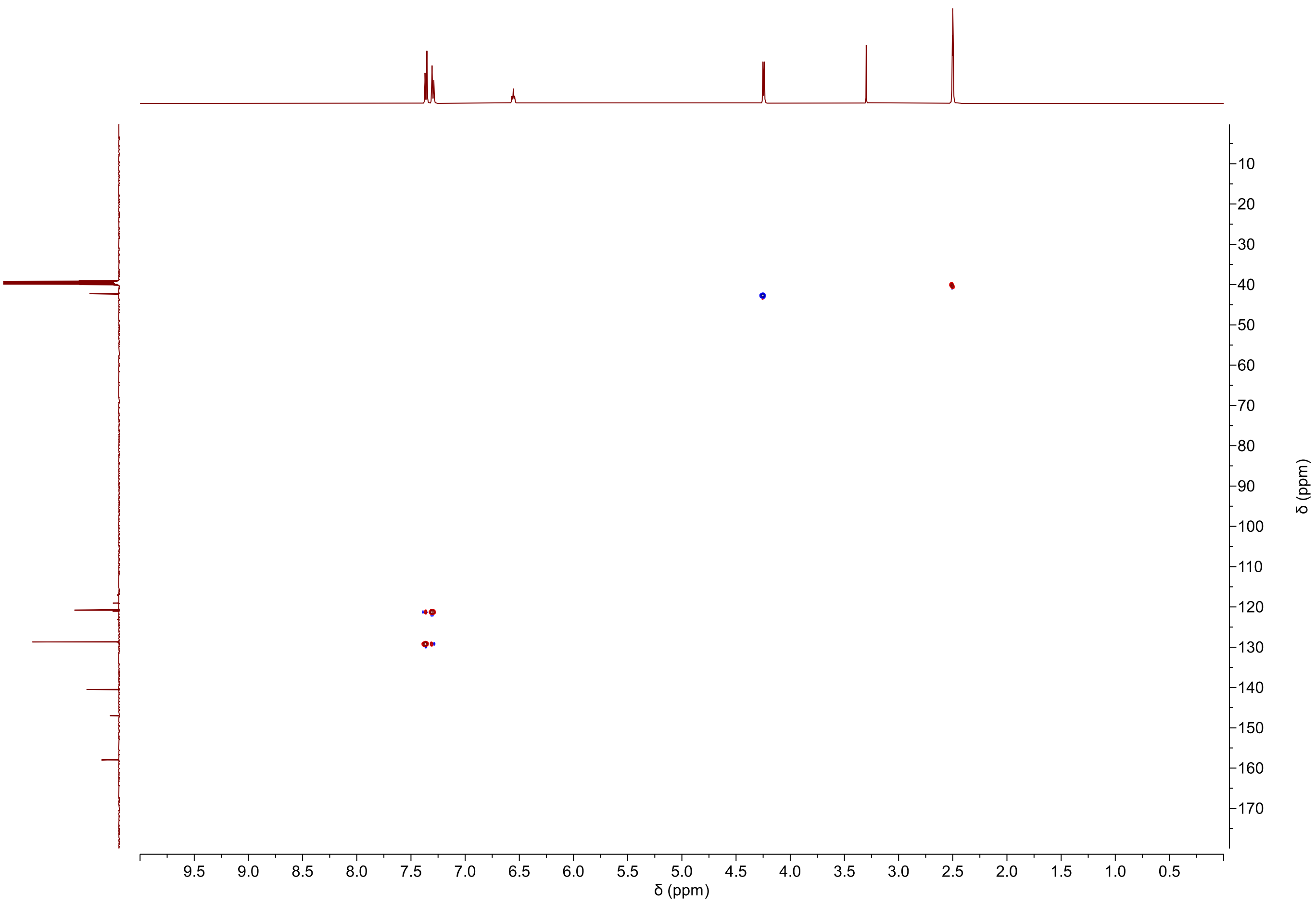

Figure S15: ^{1}H-^{13}C HSQC NMR spectrum of **8b** (500 MHz, 126 MHz, DMSO-d_6).

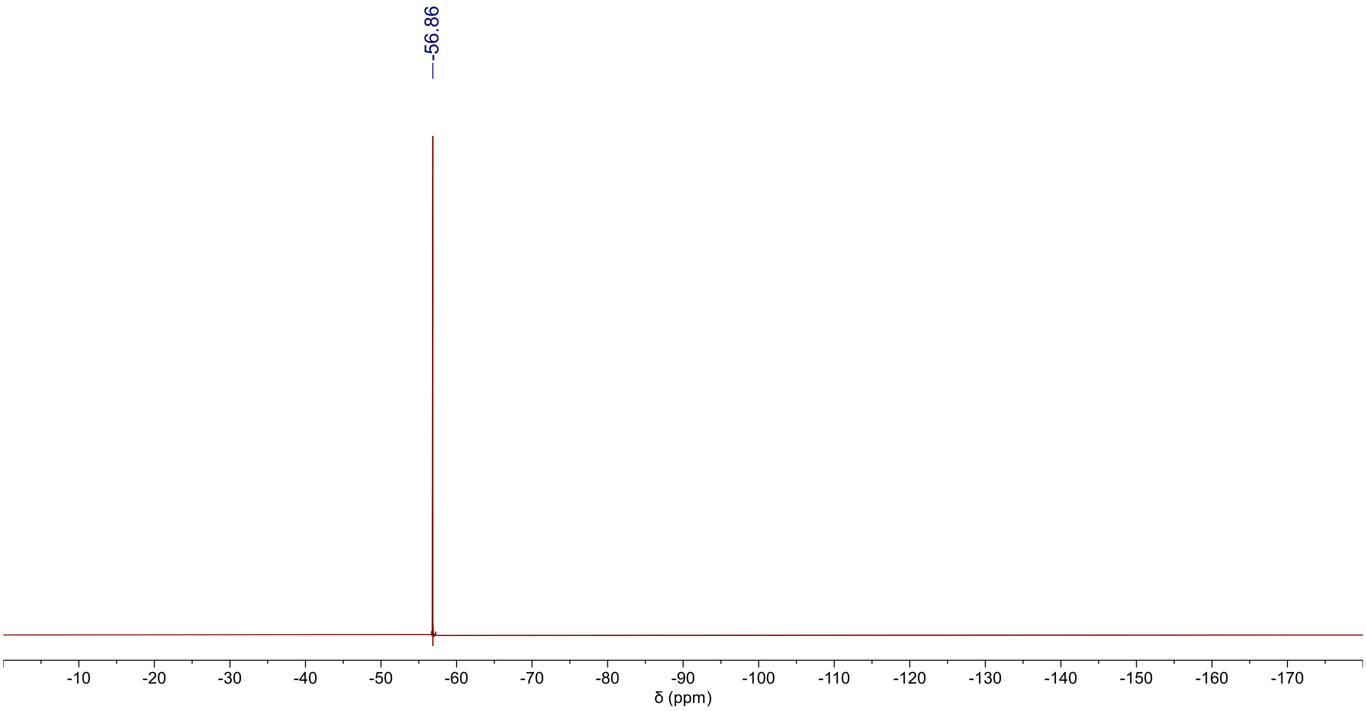

Figure S16: ^{19}F NMR spectrum of **8b** (471 MHz, DMSO-d_6).

2.5. 8c

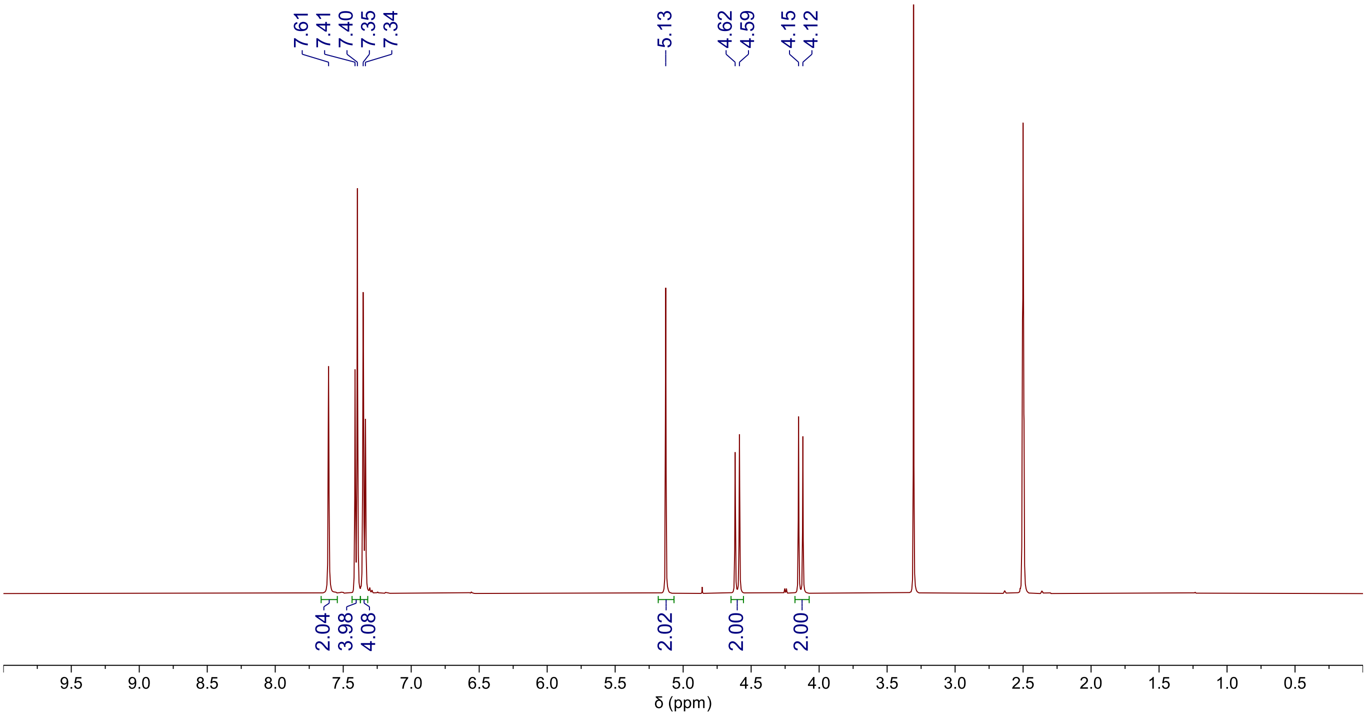

Figure S17: ^{1}H NMR spectrum of **8c** (500 MHz, DMSO-d_6).

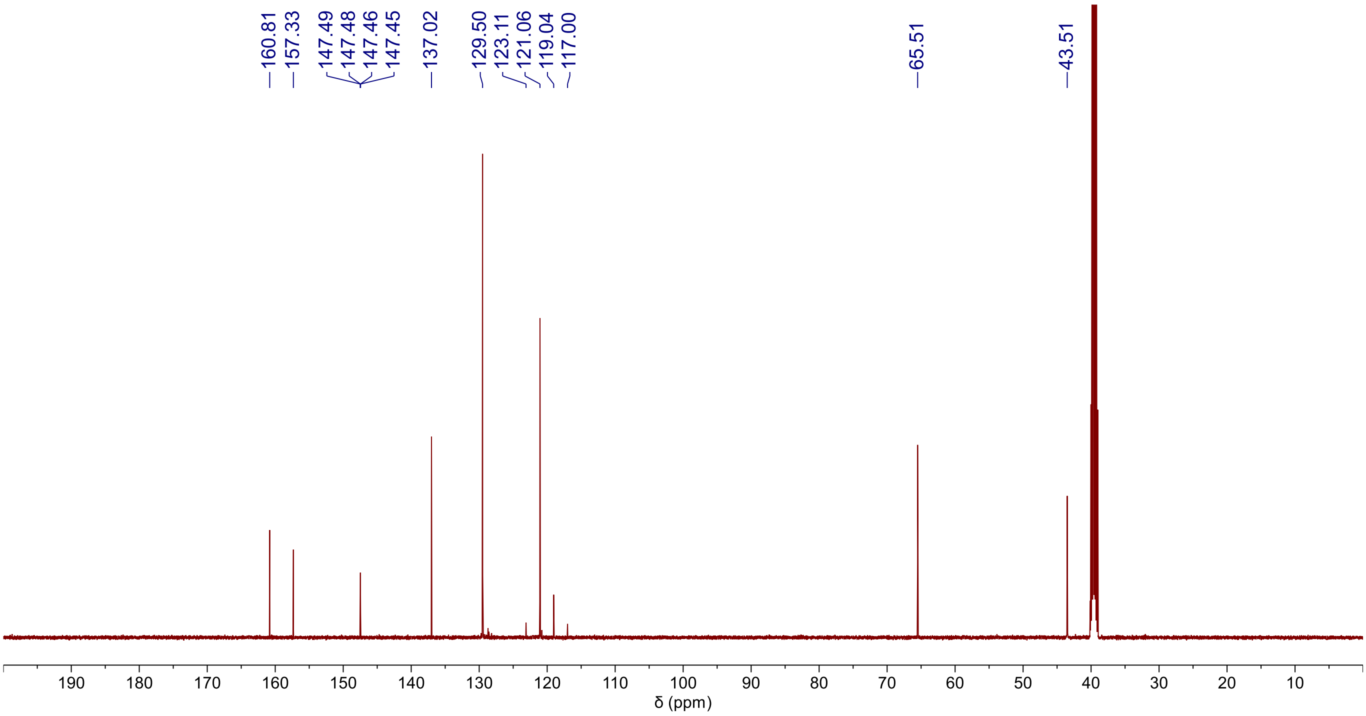

Figure S18: $^{13}C\{^{1}H\}$ NMR spectrum of **8c** (126 MHz, DMSO-d_6).

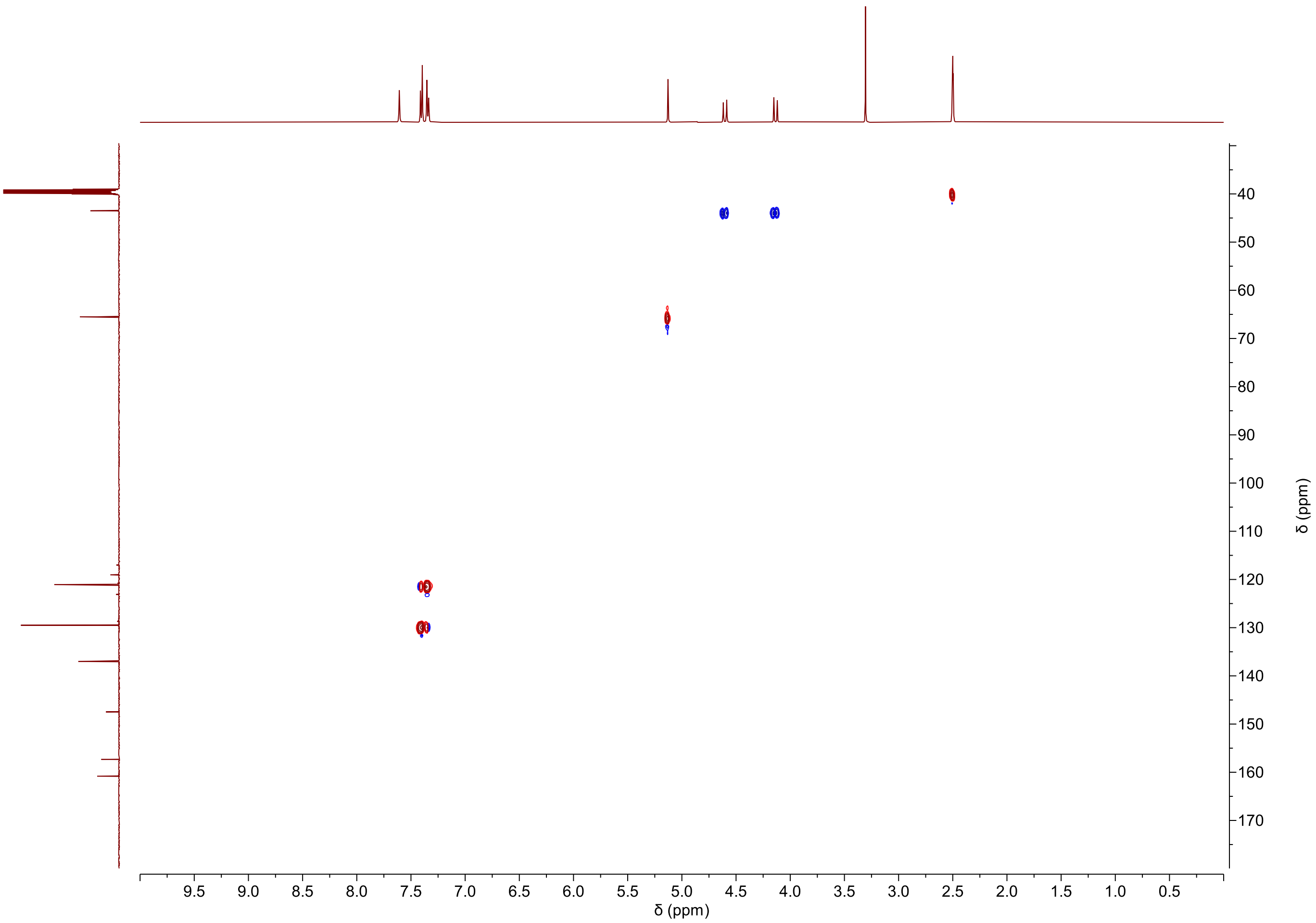

Figure S19: ^{1}H-^{13}C HSQC NMR spectrum of **8b** (500 MHz, 126 MHz, DMSO-d_6).

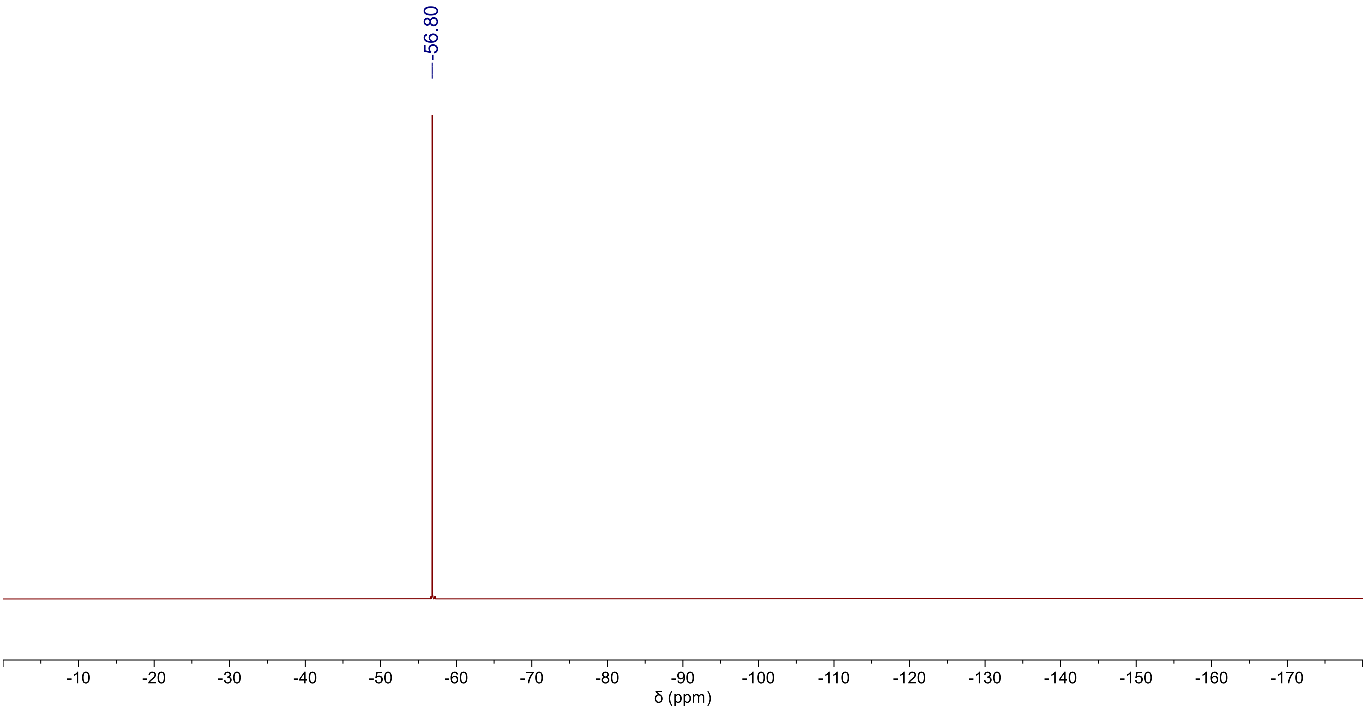

Figure S20: ^{19}F NMR spectrum of **8c** (471 MHz, DMSO-d_6).

2.6. 8

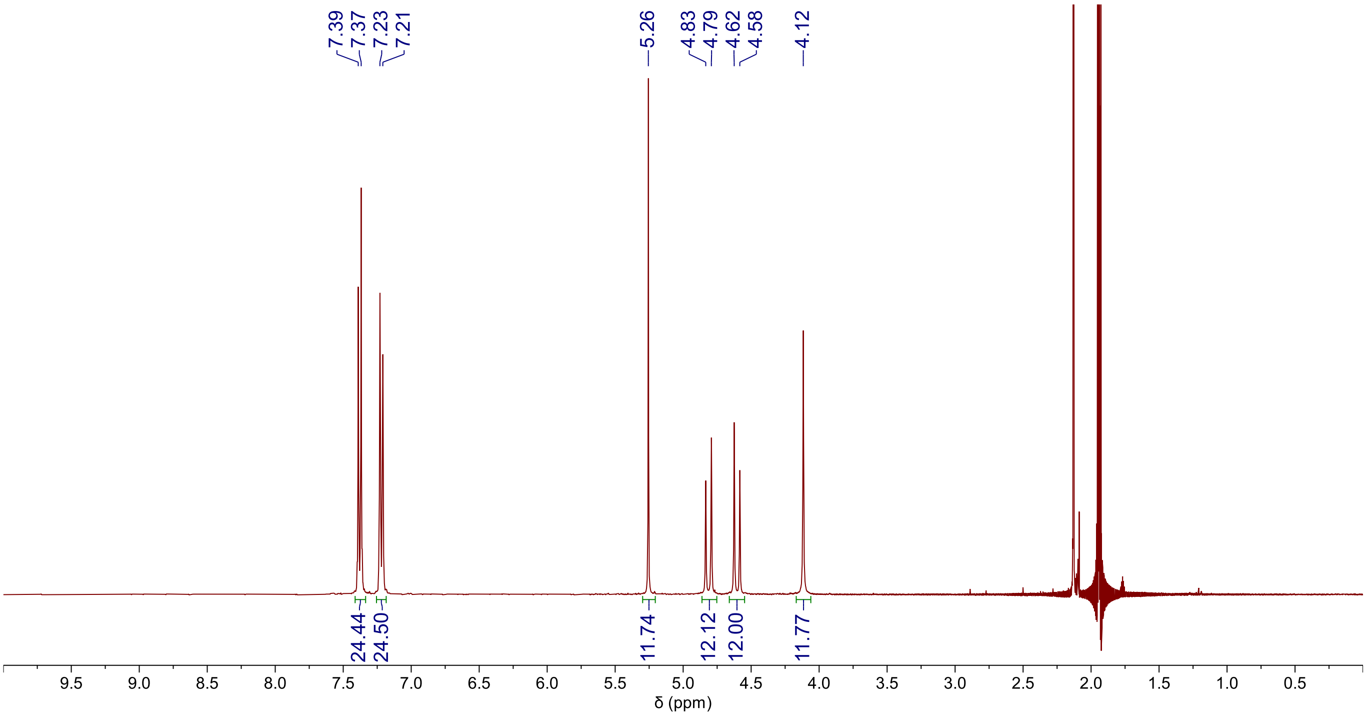

Figure S21: ^{1}H NMR spectrum of **8** (400 MHz, CD_3CN).

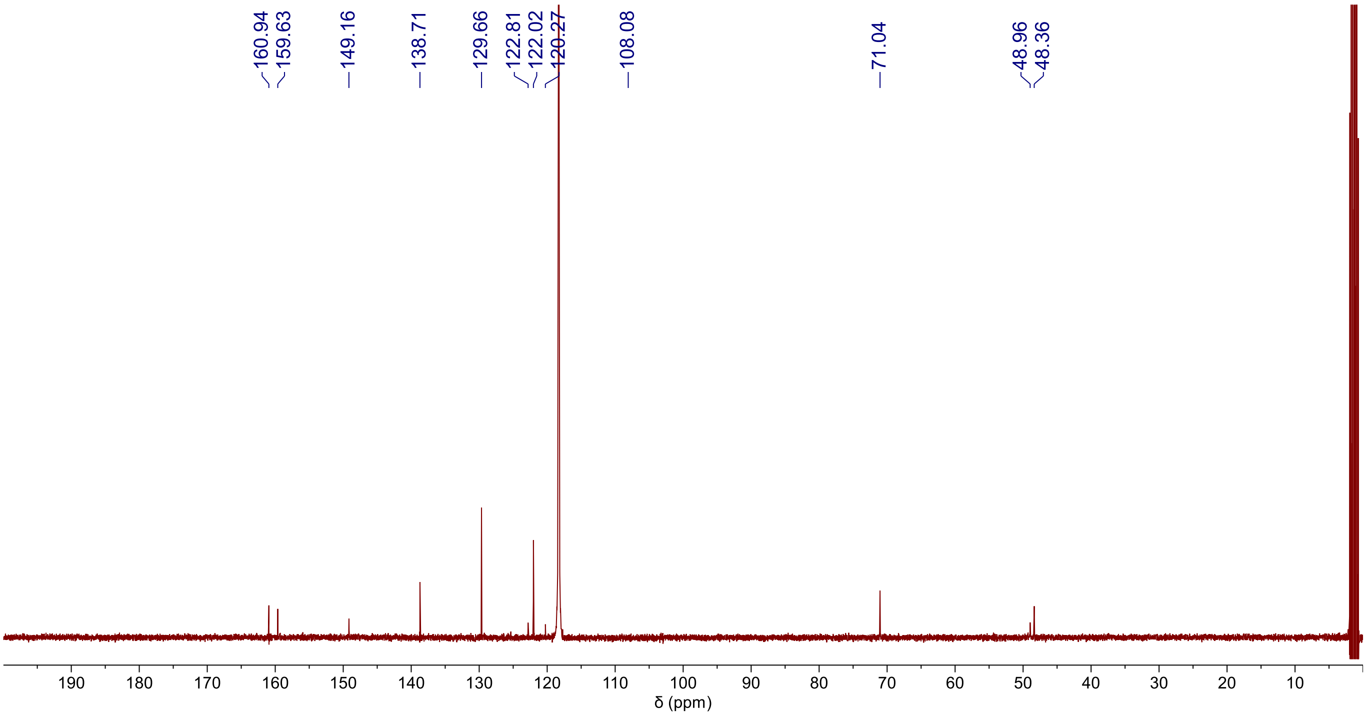

Figure S22: $^{13}C\{^{1}H\}$ NMR spectrum of **8** (101 MHz, CD_3CN).

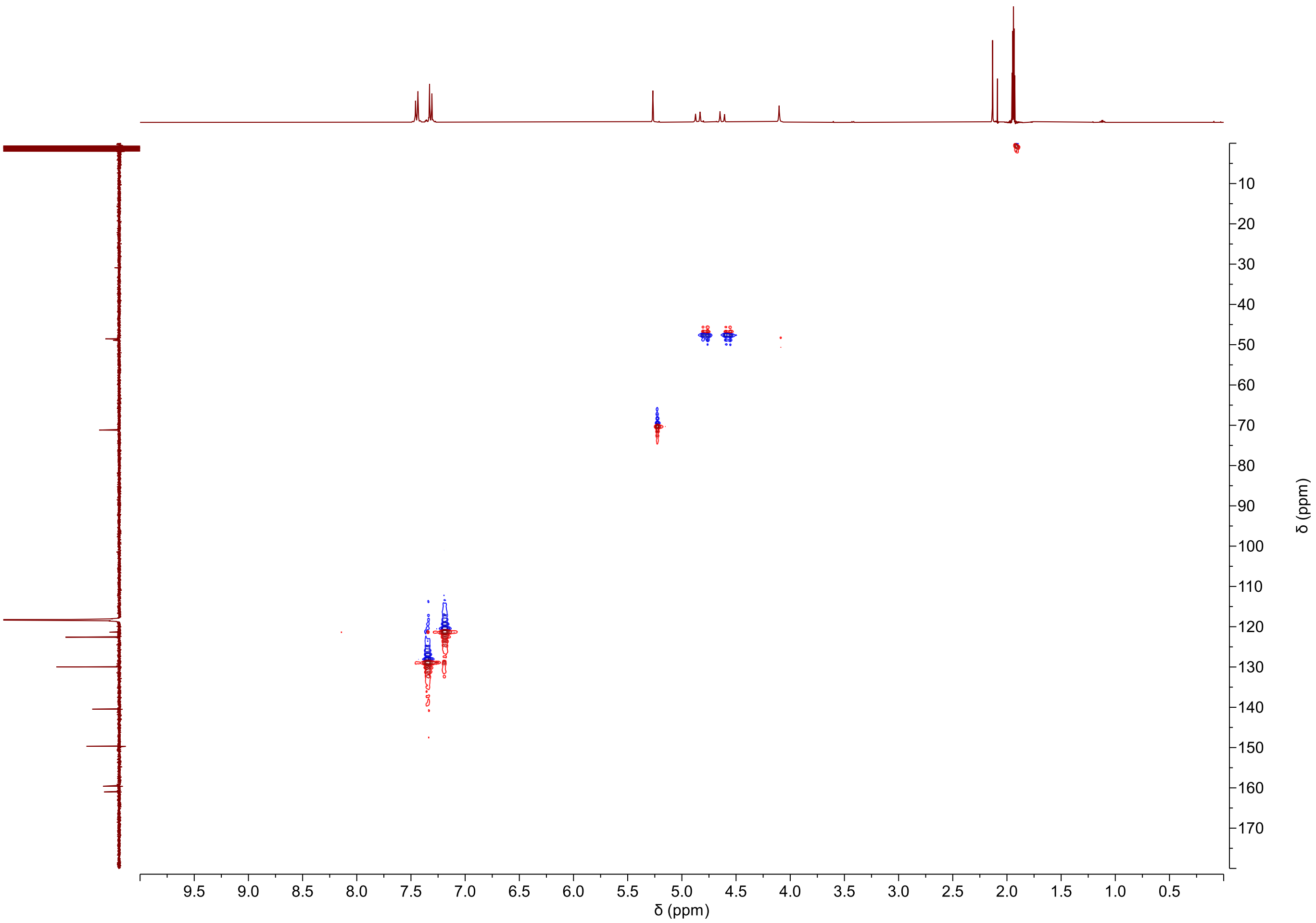

Figure S23: ^{1}H-^{13}C HSQC NMR spectrum of **8** (400 MHz, 101 MHz, CD_3CN).

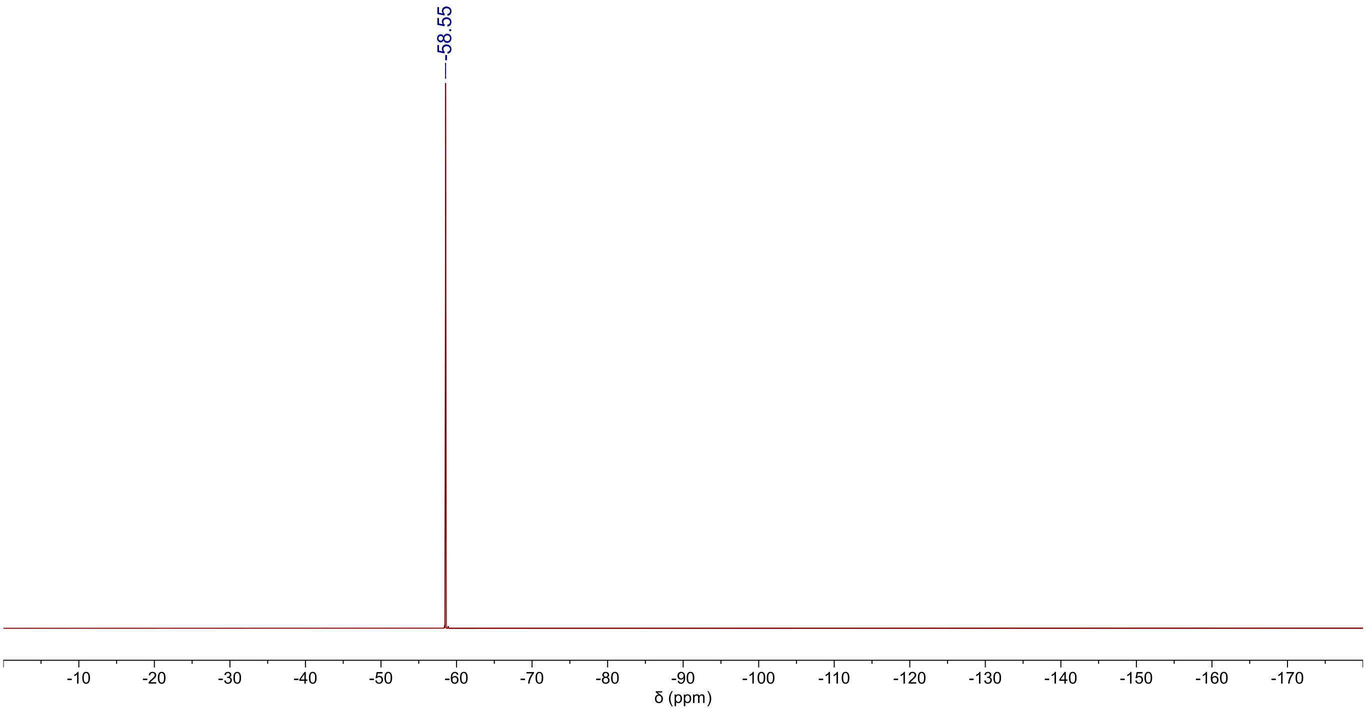

Figure S24: ^{19}F NMR spectrum of **8** (565 MHz, CD_3CN).

2.7. 11b

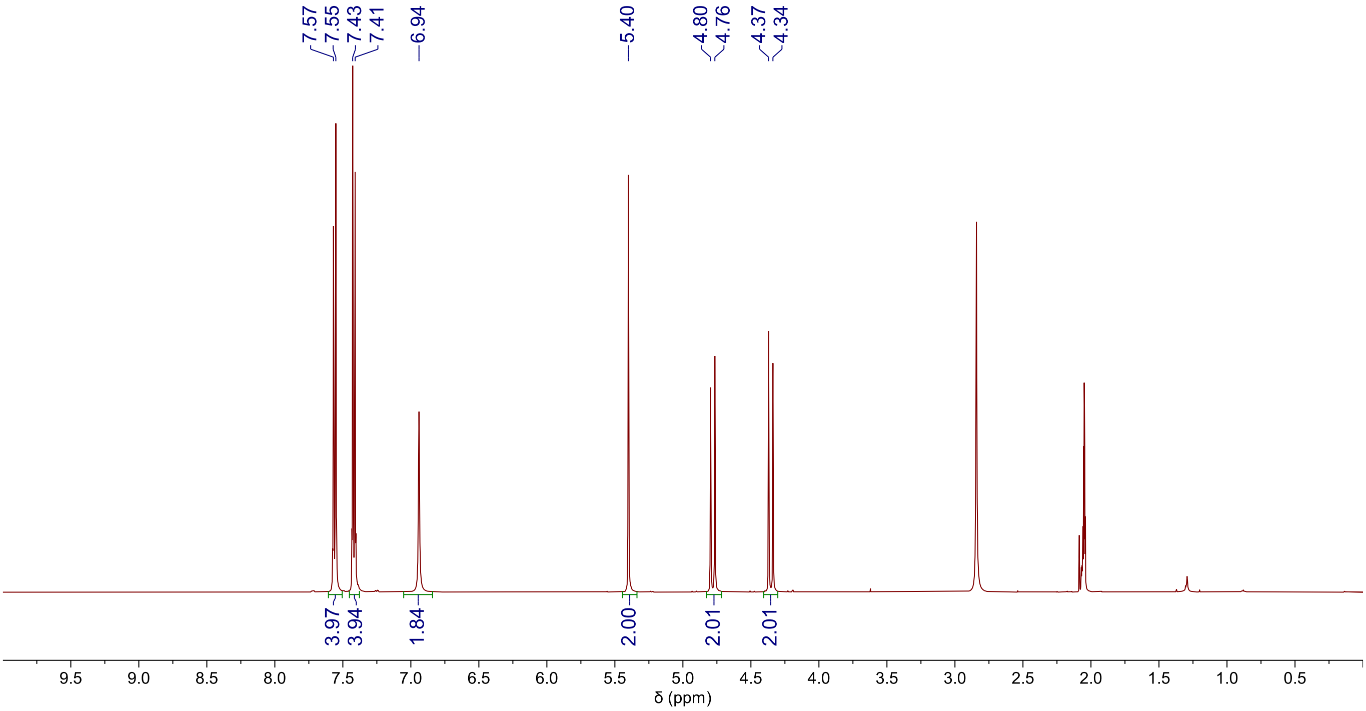

Figure S25: ^{1}H NMR spectrum of **11b** (500 MHz, acetone-d_6).

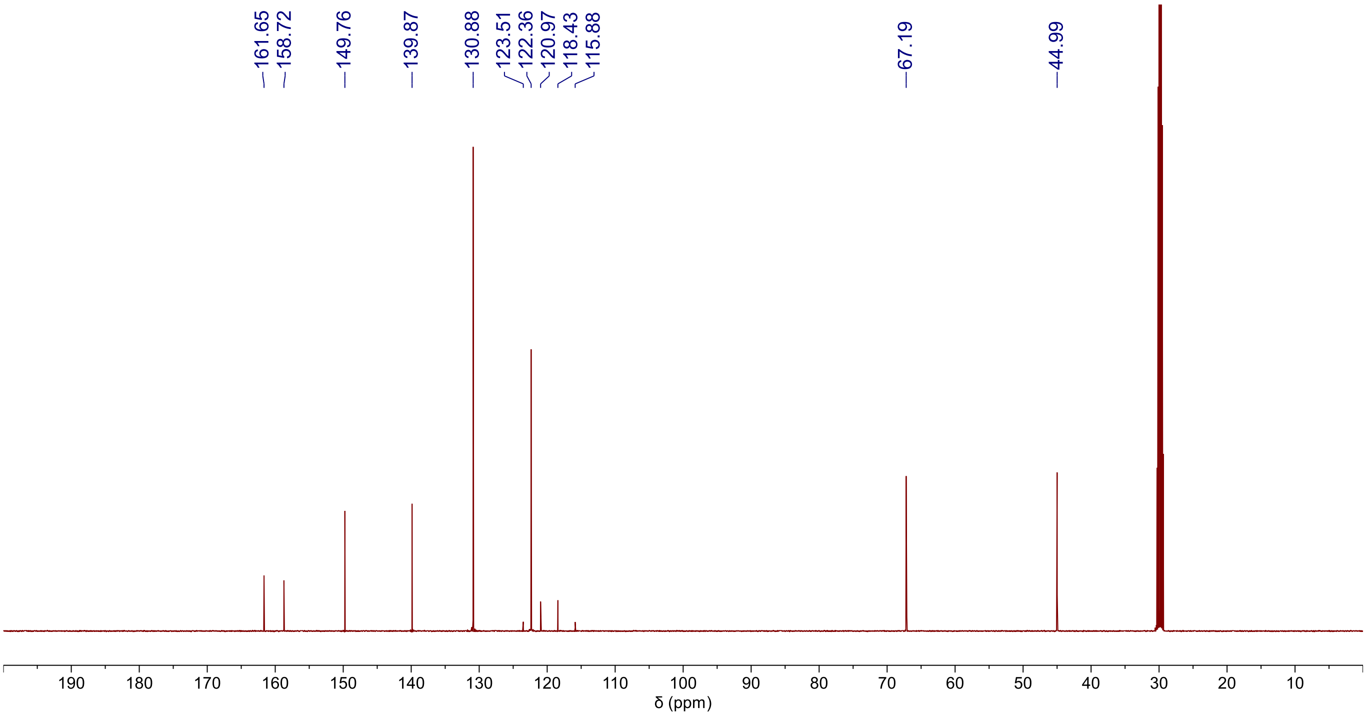

Figure S26: ^{13}C{^{1}H} NMR spectrum of **11b** (126 MHz, acetone-d_6).

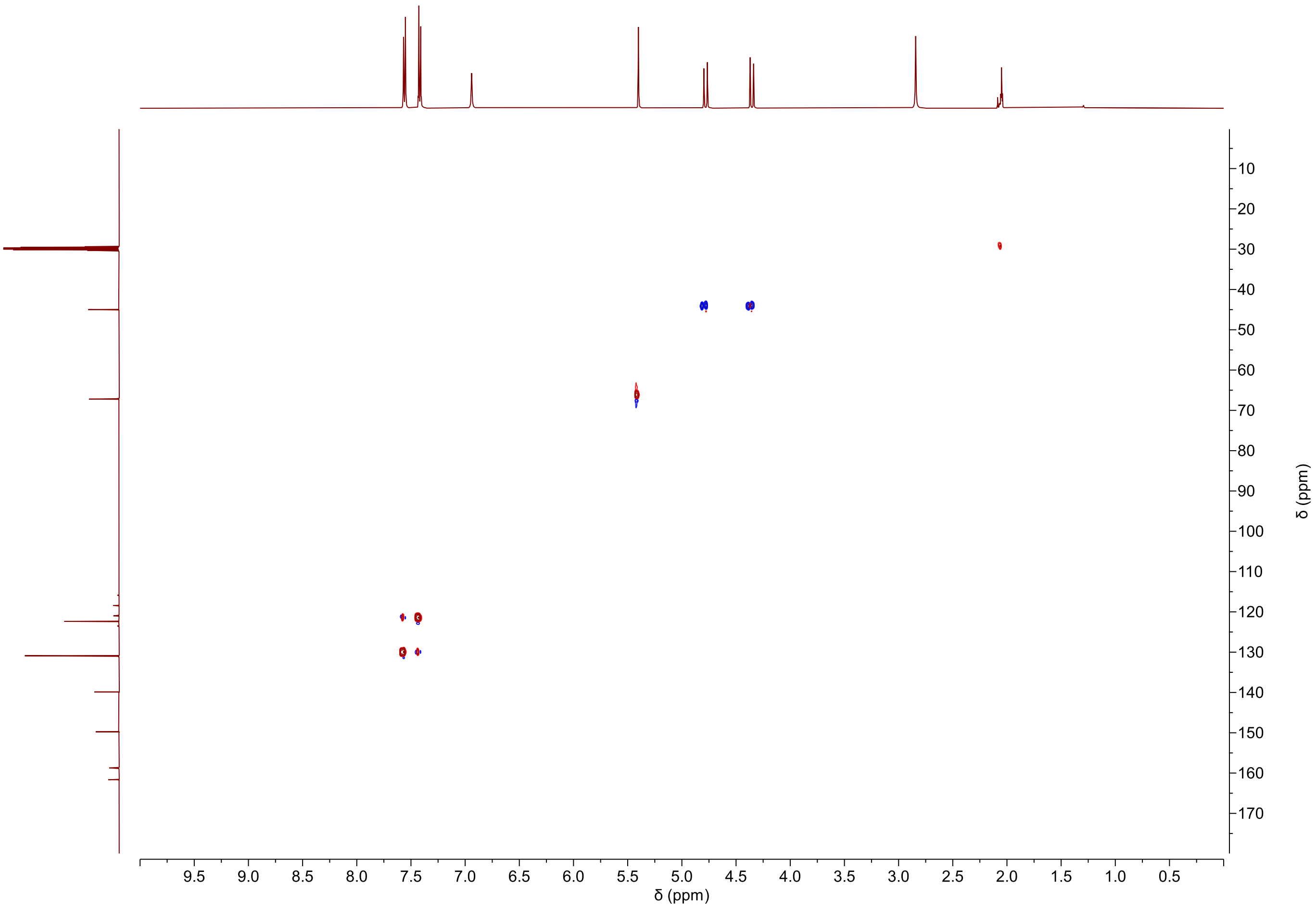

Figure S27: ^{1}H-^{13}C HSQC NMR spectrum of **11b** (500 MHz, 126 MHz, acetone-d_6).

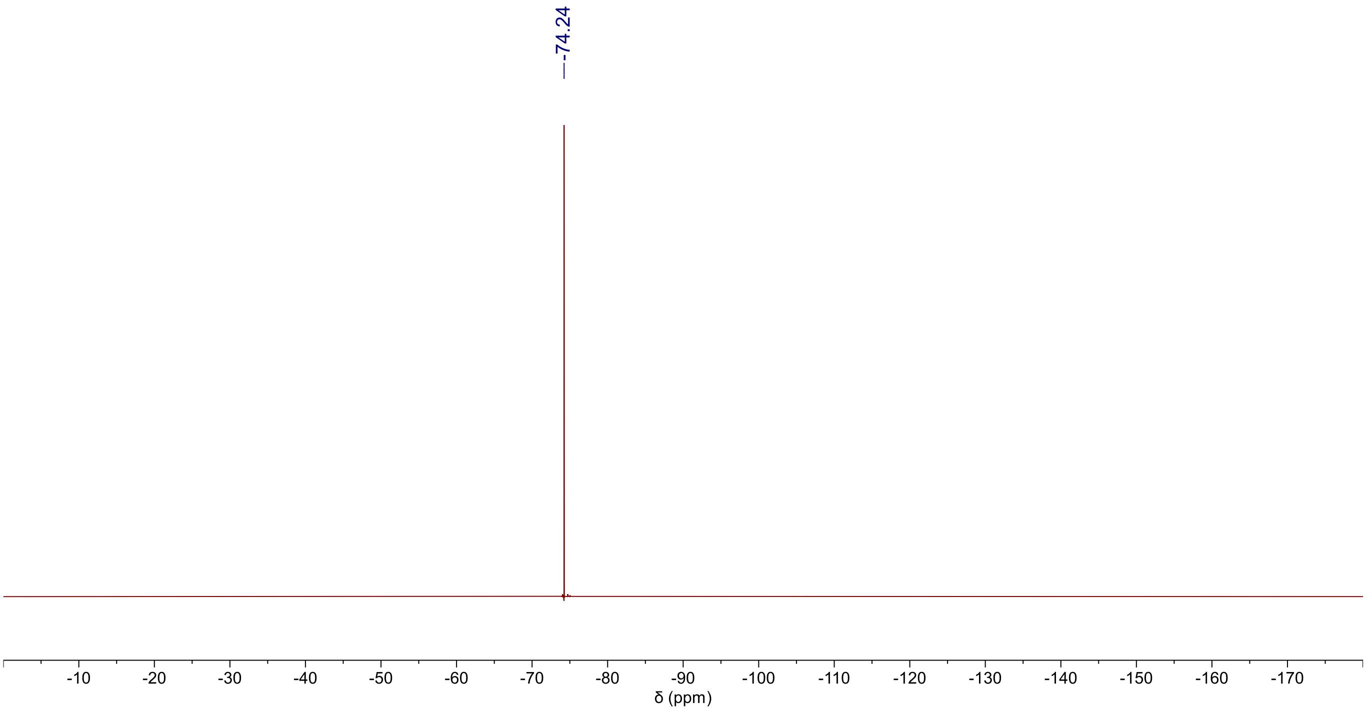

Figure S28: ^{19}F NMR spectrum of **11b** (471 MHz, acetone-d_6).

2.8. 11

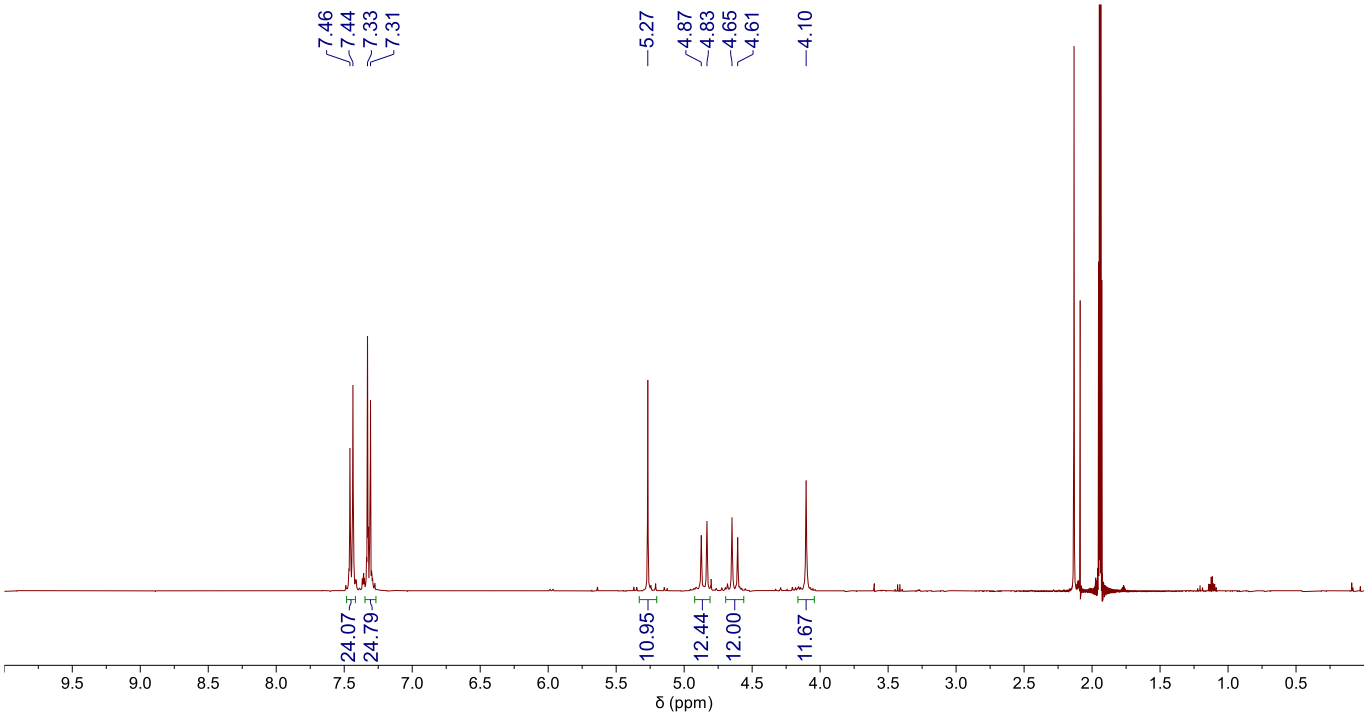

Figure S29: ^{1}H NMR spectrum of **11** (400 MHz, CD_3CN).

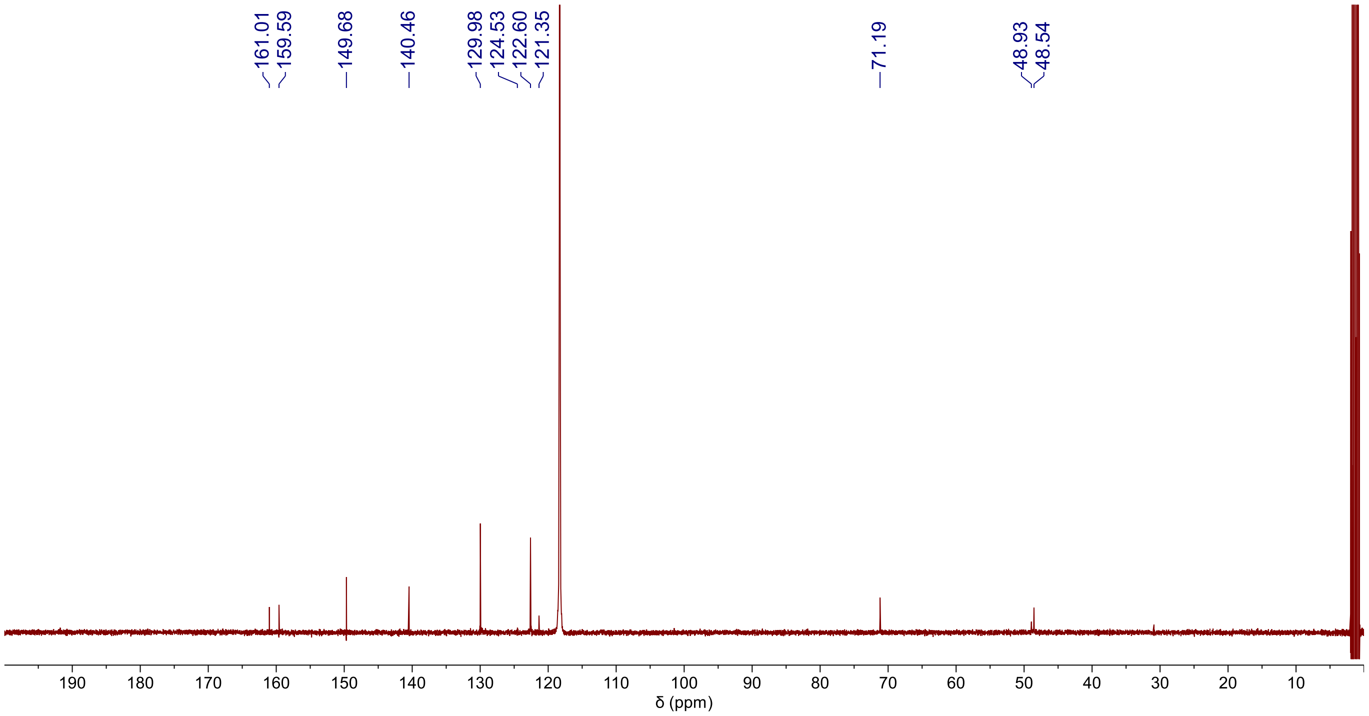

Figure S30: $^{13}C\{^{1}H\}$ NMR spectrum of **11** (101 MHz, CD_3CN).

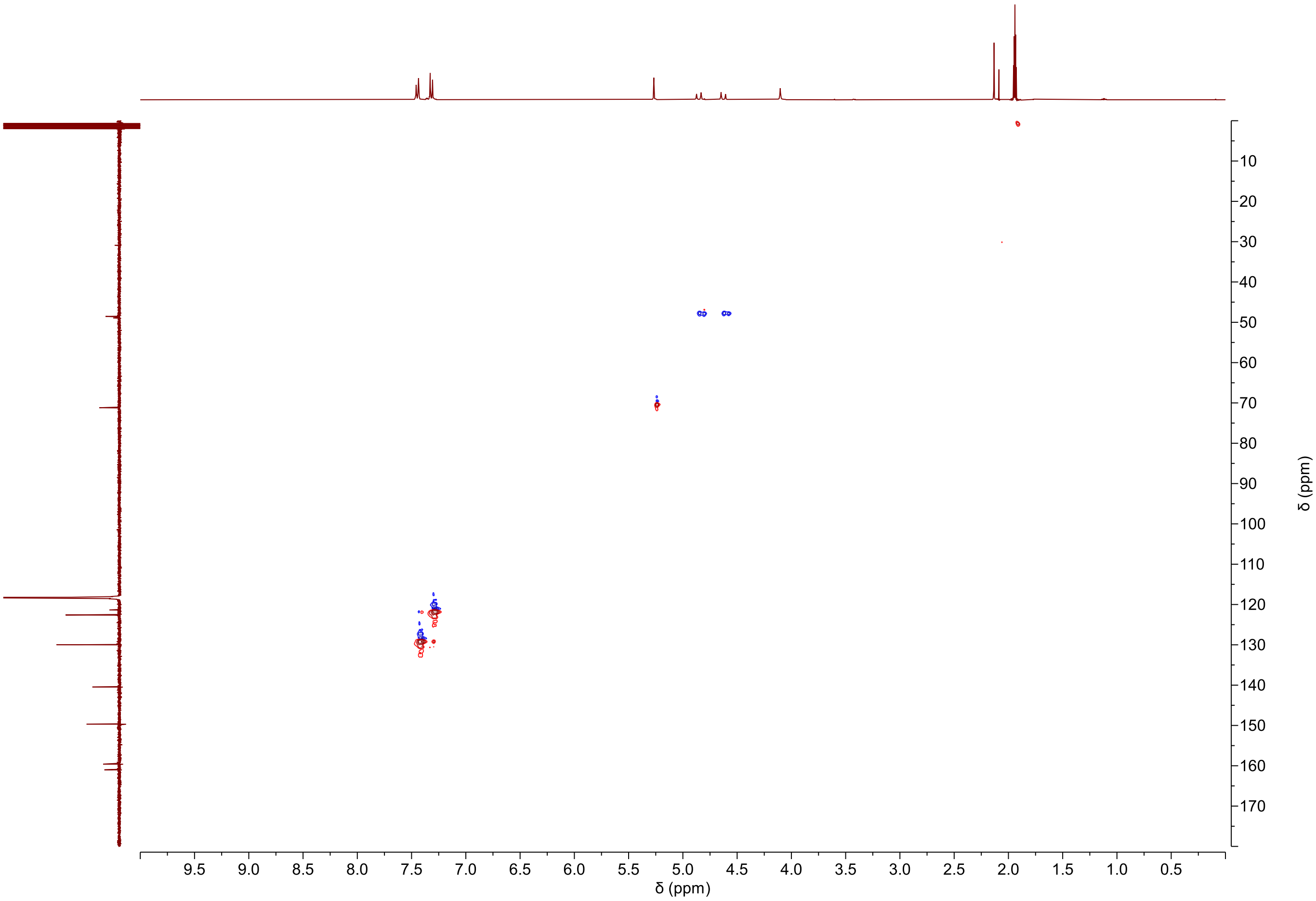

Figure S31: ^{1}H-^{13}C HSQC NMR spectrum of **11** (400 MHz, 101 MHz, CD_3CN).

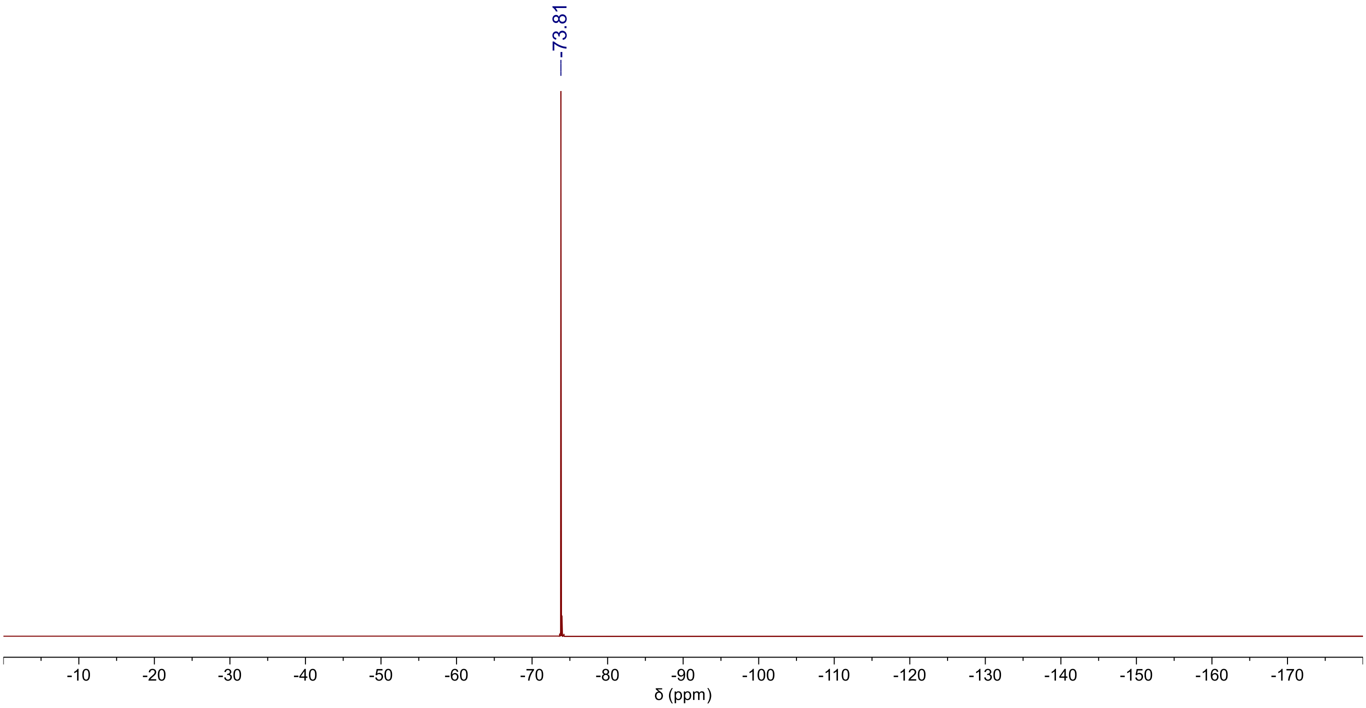

Figure S32: ^{19}F NMR spectrum of **11** (565 MHz, CD_3CN).

3. Description of the competition experiment between two BUs

To obtain binding affinities of **6**, **8**, and **11** to chloride and of **1** to iodide we have performed competition experiments with **7** as described previously.[1] We took advantage of the great resolution of ^{19}F NMR spectroscopy and the slow exchange rate of BUs with halides. As shown in Figure S34, upon mixing anion-free **7** and anion-free **8** with Bu_4NCl in CD_3CN, the chloride is distributed among these two BUs. As a result, 2 new signals form, corresponding to the Bu_4NCl complex of **7** and **8**. Simple integration of the corresponding signals and using equation (S1) gives a ratio *R* of association constants of **7** and **8**.

$$R = \frac{K_a(\mathbf{8} \cdot \mathrm{A}^-)}{K_a(\mathbf{7} \cdot \mathrm{A}^-)} = \frac{I(\mathbf{7})I(\mathbf{8} \cdot \mathrm{A}^-)}{I(\mathbf{7} \cdot \mathrm{A}^-)I(\mathbf{8})} \qquad \text{(S1)}$$

Given that the absolute association constant value for **7** is known, the absolute association constant for **8** can be determined. The uncertainty of the *R* value (described as the standard deviation σ) is obtained from several experiments using equation (S2), where R_i are the values obtained from the individual experiments and $\bar{R}$ is the average value.

$$\sigma = \sqrt{\frac{1}{N-1}\sum_{i=1}^{N}(R_i - \bar{R})^2} \qquad \text{(S2)}$$

The absolute value K_a of **8** and Bu_4NCl and related uncertainty are obtained from equations (S3) and (S4) respectively.

$$K_a(\mathbf{8}) = K_a(\mathbf{7}) \cdot R \qquad \text{(S3)}$$

$$\sigma_{K_a(\mathrm{A}^-)} = K_a(\mathrm{A}^-)\sqrt{\left(\frac{\sigma_{K_a(7)}}{K_a(\mathbf{7})}\right)^2 + \left(\frac{\sigma_R}{R}\right)^2} \qquad \text{(S4)}$$

For the logarithmic scale presented in Table 1 in the main text, the uncertainty (described by the standard deviation) was recalculated using the following equation:

$$\log_{10}\sigma_{K_a(\mathrm{A}^-)} \approx \frac{\sigma_{K_a(\mathrm{A}^-)}}{K_a(\mathrm{A}^-)\ln 10} \qquad \text{(S5)}$$

3.1. Experimental

For a typical competition experiment, two BUs are dissolved in CD_3CN and a small amount of Bu_4NX (X = Cl or I) solution in CD_3CN is added. The final concentrations of the BUs in the solution range from 50 to 400 µM. The competition is set up in such a way that the total amount of BUs is larger than the anion amount in the sample ($n(\mathbf{7})+n(\mathbf{8})>n(\mathrm{A}^-)$). The prepared sample is measured by ^{19}F qNMR spectroscopy at 298 K within an hour after its preparation. The experiments were repeated 5-7 times (Table S1) for each combination of the species.

The obtained data were processed by zero-filling to two times the original spectrum size and applying exponential apodization matching average T2*. Automatic baseline correction by polynomial fits (3rd-5th order) was used; only regions of ±10 ppm from the signals of interest were corrected. Phase correction was done when needed. Integration regions were done manually.

Anion-free and complexed forms of the individual BUs are characterized by essentially the same T1, which is lower than 1.5 seconds. The usage of a 90° pulse or precisely adjusting the pulse

frequency and relaxation delay was found not to be absolutely necessary, as only their ratio is important according to the equation (S1). On the other hand, a good signal-to-noise ratio and good separation and resolution of the integrated signals are required for the experiment. Integration regions were manually picked.

In the ^{19}F NMR spectrum of **7**, there are a few minor signals with a lower chemical shift than the main peak of the anion-free BU. The nature of these signals is explained in our previous work.[1]

For competition experiment between **6** and **7**, several experimental complications were encountered. First, complete anion removal from **6** was not successful and in the obtained sample, ca. 10% of the BU was complexed with HSO_4^-. However, as observed in our previous study,[1] the selectivity of BU towards anions does not seem to be impacted by the increase anion binding strength. Therefore, we expect that a presence of small amount of HSO_4^- anion in the competition experiment would not have a significant impact on the R value. Another complication arises from the poor resolution of the signals corresponding to the anion-free and anion-complexed forms of **6** complicating the integration of the individual signals. Therefore, the signal areas were estimated from peak fitting procedure implemented in MNova. Because of these facts, the K_a is provided as a range of values instead of a single value in Table 1 in the main text. The range was obtained as follows: σ obtained for the R value was either added to the maximal obtained or subtracted from the minimal obtained R value. These “minimal” and “maximal” R values were used in calculation of the K_a by the equation S3.

3.2. Association constants obtained from competition experiments

Table S1: Overview of all R values (ratio of association constant of **1**, **6**, **8** or **11** and association constant of **7**) obtained from the individual competition experiments.

Bambusuril	Anion	*R*
**6**	Chloride	0.578
**6**	Chloride	0.330
**6**	Chloride	0.668
**6**	Chloride	0.519
**6**	Chloride	0.291
**Average**		**0.48±0.16**
**11**	Chloride	0.658
**11**	Chloride	0.856
**11**	Chloride	0.715
**11**	Chloride	0.703
**11**	Chloride	0.732
**11**	Chloride	0.792
**11**	Chloride	0.658
**Average**		**0.74±0.07**

Bambusuril	Anion	*R*
**1**	Iodide	8.592
**1**	Iodide	9.260
**1**	Iodide	8.412
**1**	Iodide	6.972
**1**	Iodide	9.108
**1**	Iodide	8.071
**1**	Iodide	9.063
**Average**		**8.5±0.8**
**8**	Chloride	0.117
**8**	Chloride	0.096
**8**	Chloride	0.104
**8**	Chloride	0.095
**8**	Chloride	0.116
**Average**		**0.11±0.01**

Table S2: Overview of the average association constants obtained from the competition experiments.

**Bambusuril**	**Anion**	**log K_a**
**1**	Iodide	13.1±0.2
**6**	Chloride	9.7-10.5
**7**	Chloride	10.6±0.2[1]
**8**	Chloride	9.7±0.2
**11**	Chloride	10.5±0.2

Table S3: Overview of the association constant of **1** towards various anions in CD_3CN.

Anion	K_a in MeCN (M^{-1})	Method	Reference
Cl^-	$(2±1)×10^{11}$	Competition with **7**	Chvojka, 2025[2]
Cl^-	$(6±1)×10^{10}$	NMR titration*	Valkenier, 2019[4]
Br^-	$(4±2)×10^{12}$	Competition with **7**	Chvojka, 2025[2]
I^-	$(1.4±0.7)×10^{13}$	Competition with **7**	This work
HCO_3^-	$>2×10^9$ (1:1)<br>$(1.8±0.1)×10^4$ (1:2)	NMR titrations	Valkenier, 2019[4]
NO_3^-	8-times stronger than Cl^-	NMR titration, competition with Cl^-	Valkenier, 2019[4]

*Titration with $CF_3SO_3^-$, followed by competitions with ReO_4^-, SCN^-, and Cl^-.

3.3. Examples of the results obtained from the competition experiment

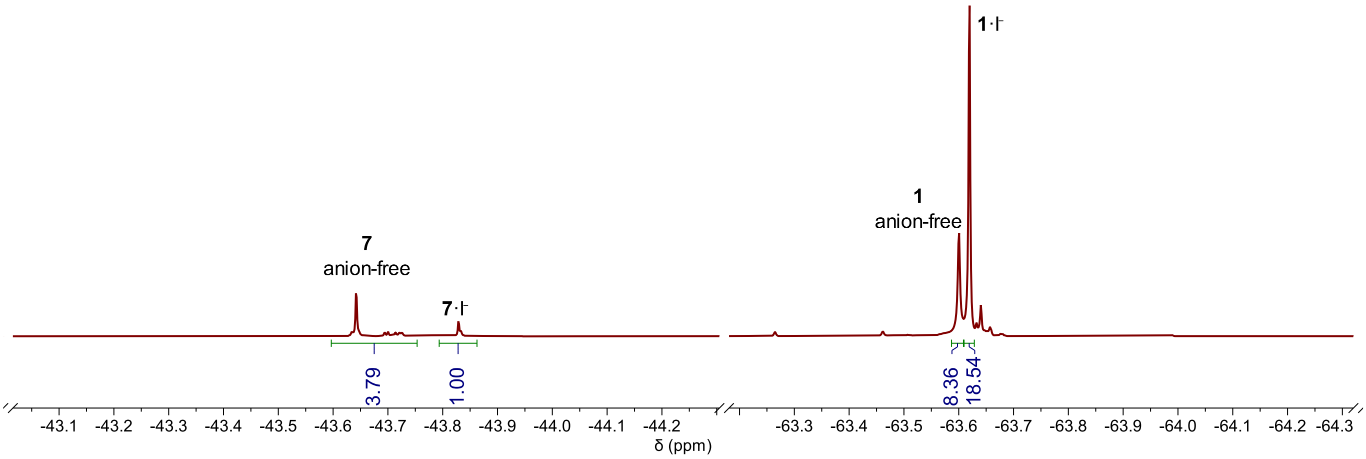

Figure S33: ^{19}F NMR spectra (565 MHz, CD_3CN) of the competition experiment between **1** with **7 for** Bu_4NI, giving $K_a(\mathbf{1})/K_a(\mathbf{7}) = 8.41$.

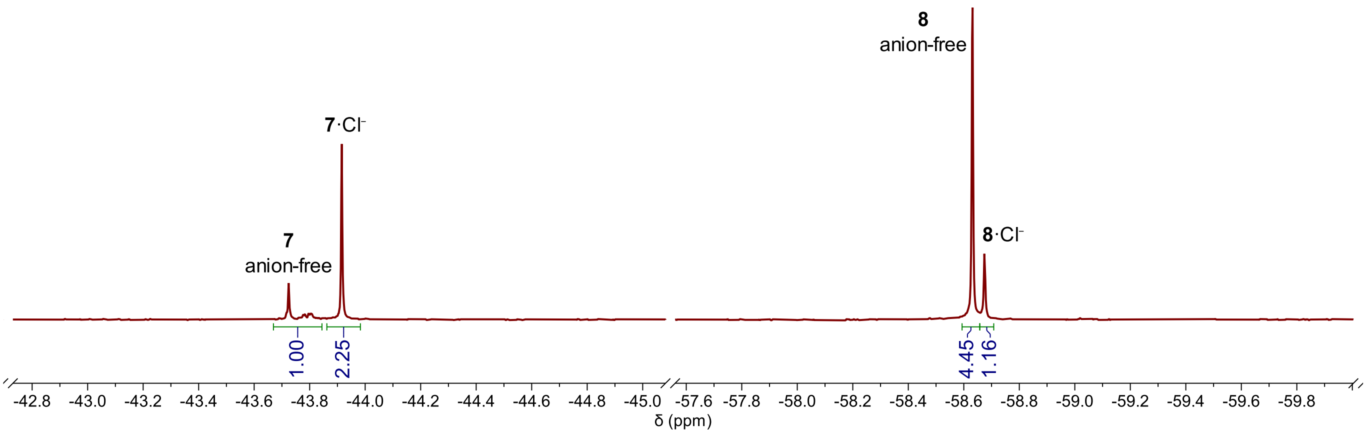

Figure S34: ^{19}F NMR spectra (471 MHz, CD_3CN) of the competition experiment between **7** with **8** for Bu_4NCl, giving K_a(**8**)/K_a(**7**) = 0.12.

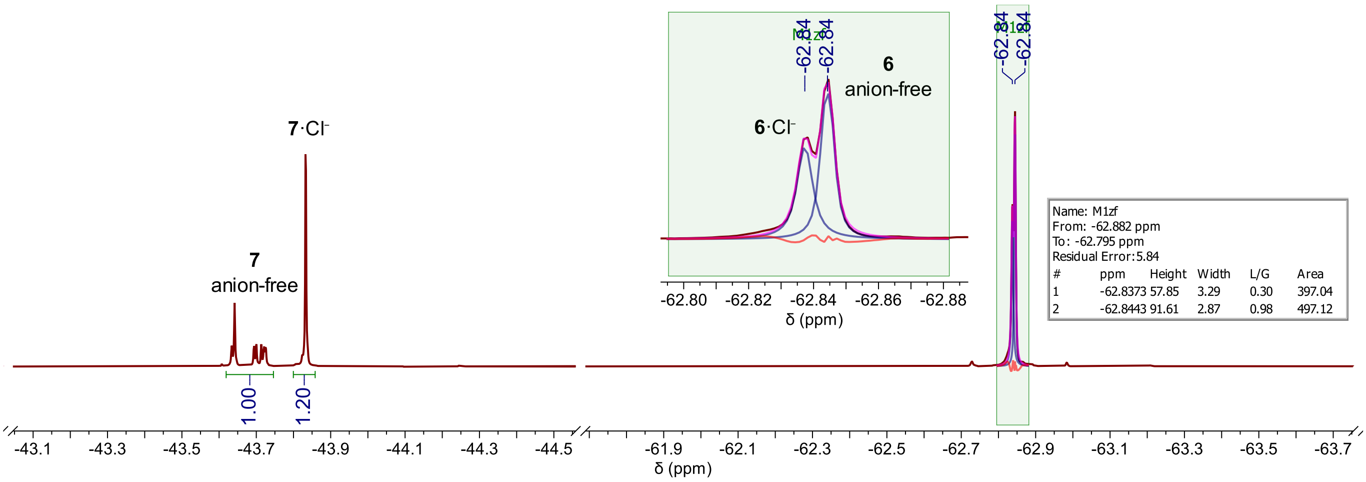

Figure S35: ^{19}F NMR spectra (565 MHz, CD_3CN) of the competition experiment between **6** with **7** for Bu_4NCl, giving K_a(**6**)/K_a(**7**) = 0.67.

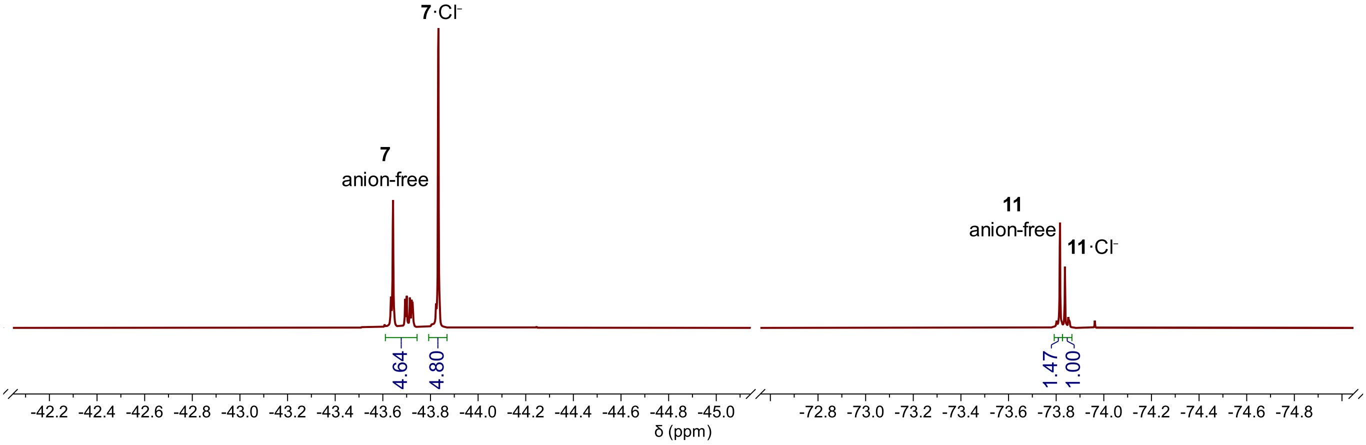

Figure S36: ^{19}F NMR spectra (565 MHz, CD_3CN) of the competition experiment between **7** with **11** for Bu_4NCl, giving K_a(**11**)/K_a(**7**) = 0.66.

4. Anion transport experiments

4.1. Materials and methods

The anion transport ability of the prepared BUs was assessed by using lucigenin, SPBA and HPTS assays.[5,6] The stock solutions of phosphatidylcholines (PC) and cholesterol were prepared in chloroform freshly deacidified by passing through a column of basic alumina. The PC solutions were stored below −20 °C, the cholesterol solution was prepared fresh. All aqueous solutions were prepared using deionized water passed through mili-Q water system.

Lipid solutions were combined with a solution of a tested BU (30-3000 μM in acetonitrile or acetone) in a 5 mL round bottom flask. The added volumes were calculated from the concentrations of the lipids in chloroform to obtain the desired PC to cholesterol ratio and the desired BU to total lipid ratio. The solvents were evaporated under a nitrogen stream and the resulting lipid film was dried under a high vacuum for at least 1 h to overnight. The lipid film was then hydrated with 500 μL of a solution containing the desired salt and probe. The resulting mixture was sonicated for ca. 30 s and stirred for 1 h to give heterogeneous vesicles. Multilamellar vesicles were disrupted by 10 freeze-thaw cycles. The mixture was diluted to 1 mL with an additional 500 μL of the salt solution and carefully extruded 29 times through a polycarbonate membrane with 200 nm pores in a mini-extruder (Avestin LiposoFast-Basic). The external probe was removed by passing the liposome solutions through a pre-packed size exclusion column (containing 8.3 mL of Sephadex G-25 medium) and eluted with the salt solution. The collected large unilamellar vesicles were further diluted with a salt solution to obtain a solution with the desired total lipid concentration (assuming no lipids were lost during the liposome preparation). The obtained liposome solutions were used for transport measurements on the same day.

For the transport experiments, the liposome solution (3 mL) was placed in a quartz cuvette with a small stir bar. For the lucigenin and SPBA assays, the temperature was allowed to stabilize at 25 °C for 3-5 min inside the sample compartment of a Fluoromax 4 spectrophotometer, then the measurement started. For the HPTS assay, the transport measurements were done using Agilent Cary Eclipse spectrophotometer at room temperature. Fluorescence data were collected for two to four runs per sample. More details for each type of experiment are given below.

The size of the liposomes was verified by Dynamic Light Scattering (DLS) measurements on a Malvern Zetasizer Ultra at 25 °C, using disposable cuvettes (Figure S37).

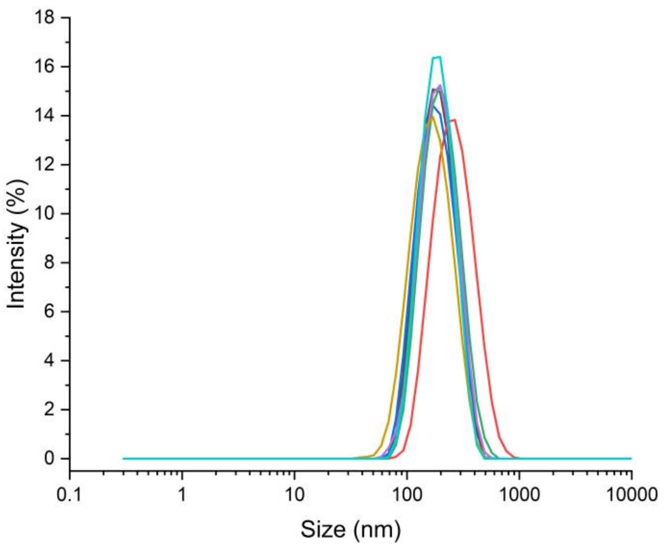

Figure S37: DLS data for 7 various batches of LUVs used in this work.

4.1.1. Lucigenin assay to study Cl^-/HCO_3^- antiport

$NaHCO_3$ buffer (225 mM) was prepared in mili-Q water and its pH was adjusted to 7.5-7.8 by the addition of concentrated H_2SO_4. It was used only the same day as it was prepared. The lipid film was hydrated with 500 µL of a solution containing $NaHCO_3$ buffer (225 mM) and 10,10'-dimethyl-9,9'-biacridinium nitrate (lucigenin, 0.8 mM). The liposomes were composed of 1-palmitoyl-2-oleoyl-*sn*-glycero-3-phosphocholine (POPC) only or POPC/cholesterol at 7:3 ratio and the tested BU. BU was preincorporated at 1:5000 or at 1:50 000 BU to total lipid ratio. The final lipid concentration was 0.4 mM.

For the transport experiment, the fluorescence intensity of lucigenin (emission 505 nm, excitation 430 nm) was measured over time at 25 °C while stirring the solution. 3.0 mL of liposome solution was used for a single run. 76.9 µL of NaCl solution (1.0 M in $NaHCO_3$ buffer) was added 30 s after the start of the measurement to obtain the final 25 mM concentration of Cl^-. For X^-/HCO_3^- antiport studies of **1**, NaBr or NaI were used instead of NaCl. The fluorescence intensity was measured for another 10 minutes, followed by lysing of the liposomes upon the addition of 50 µL of Triton X-100 (5% w/w in water).

4.1.2. Lucigenin and SPBA assays to study X^-/NO_3^- antiport by 1

$NaNO_3$ buffer (225 mM) was prepared in mili-Q water. The lipid film was hydrated with 500 µL of a solution containing $NaNO_3$ (225 mM) and lucigenin (0.8 mM) or 10,10'-bis(3-sulfonatopropyl)-9,9'-biacridinium (SPBA, 0.8 mM). The liposomes were composed of POPC/cholesterol at 7:3 ratio. **1** was preincorporated at 1:1000 **1** to total lipid ratio. The final lipid concentration was 0.4 mM.

For the transport experiment, the fluorescence intensity of lucigenin or SPBA (emission 505 nm, excitation 430 nm) was measured over time at 25 °C while stirring the solution. 3.0 mL of liposome solution was used for a single run. 76.9 µL of NaX solution (X = Cl, Br or I; 1.0 M in $NaNO_3$ buffer) was added 30 s after the start of the measurement to obtain the final 25 mM concentration of X^-. The fluorescence intensity was measured for another 10 minutes, followed by lysing of the liposomes upon the addition of 50 µL of Triton X-100 (5% w/w in water).

4.1.3. SPBA assay to study Cl^-/NO_3^- antiport by **1** in liposomes made of various length lipids

$NaNO_3$ buffer (225 mM) was prepared in mili-Q water. The lipid film was hydrated with 500 µL of a solution containing $NaNO_3$ (225 mM) and SPBA (0.8 mM). The liposomes were composed of PC/cholesterol at 7:3 ratio and **1** preincorporated at 1:1000 **1** to total lipid ratio. One of the following PCs was used: 1,2-dimyristoleoyl-*sn*-glycero-3-phosphocholine (14:1 (Δ9-Cis) PC); 1,2-palmitoleoyl-*sn*-glycero-3-phosphocholine (16:1 (Δ9-Cis) PC); 1,2-dioleoyl-*sn*-glycero-3-phosphocholine (18:1 (Δ9-Cis) PC (DOPC)); 1,2-dieicosenoyl-*sn*-glycero-3-phosphocholine (20:1 (Δ11-Cis) PC); 1,2-dierucoyl-*sn*-glycero-3-phosphocholine (22:1 (Δ13-Cis) PC). The final lipid concentration was 0.2 mM.

For the transport experiment, the fluorescence intensity of SPBA (emission 505 nm, excitation 430 nm) was measured over time at 25 °C while stirring the solution. 2.5 mL of liposome solution was used for a single run. 64.1 µL of NaCl solution (1.0 M in $NaNO_3$ buffer) was added 30 s after the start of the measurement to obtain the final 25 mM concentration of Cl^-. The fluorescence intensity was measured for another 10 minutes, followed by lysing of the liposomes upon the addition of 50 µL of Triton X-100 (5% w/w in water).

4.1.4. HPTS assay to study A^- uniport by **1**

The buffer solution was composed of NaCl or Na_2SO_4 (100 mM), 4-(2-hydroxyethyl)-1-piperazineethanesulfonic acid (HEPES) (10 mM) and adjusted to pH 6.8 by the addition of aqueous NaOH or HCl (or H_2SO_4) solutions. For the experiments with $NMDGH^+$ cation, the buffer solution was prepared by mixing a solution of *N*-methyl-D-glucamine (NMDG) (100 mM) and HEPES (10 mM) with the appropriate amounts of HCl, HNO_3 or H_2SO_4, until pH 6.8 was reached. The lipid film was then hydrated with 500 µL of a solution containing the desired salt and 8-hydroxypyrene-1,3,6-trisulfonic acid (HPTS, 1 mM). The liposomes were composed of POPC/cholesterol at 7:3 ratio and **1** preincorporated at the desired **1** to total lipid ratio. The final lipid concentration was 0.1 mM.

For the transport experiment, the fluorescence intensity was measured over time at the ambient temperature while stirring the solution. 3.0 mL of liposome solution was used for a single run. Two or three runs were measured in parallel. If the cationophore carbonyl cyanide 3-chlorophenylhydrazone (CCCP) was used, it was added as 5 µL of its stock solution in MeOH (60 µM) to reach 1:1000 transporter to lipids ratio and the solution was stirred for 3-5 min. 30.3 µL of NaOH or NMDG solution (0.5 M in water) was added 30 s after the start of the measurement to obtain the final 5 mM concentration of the base (pH 7.8). The fluorescence intensity was measured for another 3-10 minutes, followed by lysing of the liposomes upon the addition of 50 µL of Triton X-100 (5% w/w in water). The rate of the pH gradient dissipation was monitored by recording the ratio between the fluorescence emissions (510 nm) of the protonated (excitation at 403 nm) and deprotonated (excitation at 455 nm) forms of HPTS (F_{455}/F_{403}).

4.1.5. Analysis of the transport curves obtained for studies using lucigenin or SPBA assays

Fitting of the transport curves obtained from the lucigenin and SPBA assays to obtain transport rates was done as follows. The plateau (before the pulse addition) and the vertical drop (the first 1-3 seconds after the pulse addition, due to quenching of external probe) were removed.

Fluorescence values between 0-500 s from the individual runs were averaged and the data were normalized: the fluorescence value at t = 0 s (F_0) was divided by the fluorescence values at the time t (F). The normalized trace was then fitted by a single exponential:

$$\frac{F_0}{F} = y_\infty - ae^{-kt} \tag{6}$$

Where t is the time, y_∞, a and k are the fitting parameters, k being the transport rate constant.

To obtain initial transport rate values, the normalized trace was fitted by a double exponential:

$$\frac{F_0}{F} = y_\infty - ae^{-bt} - ce^{-dt} \tag{7}$$

Upon differentiation by t and substituting t = 0, the equation for the initial transport rate is obtained:

$$I = ab + cd \tag{8}$$

An example of a fitted curve is shown in Figure S38.

a) b)

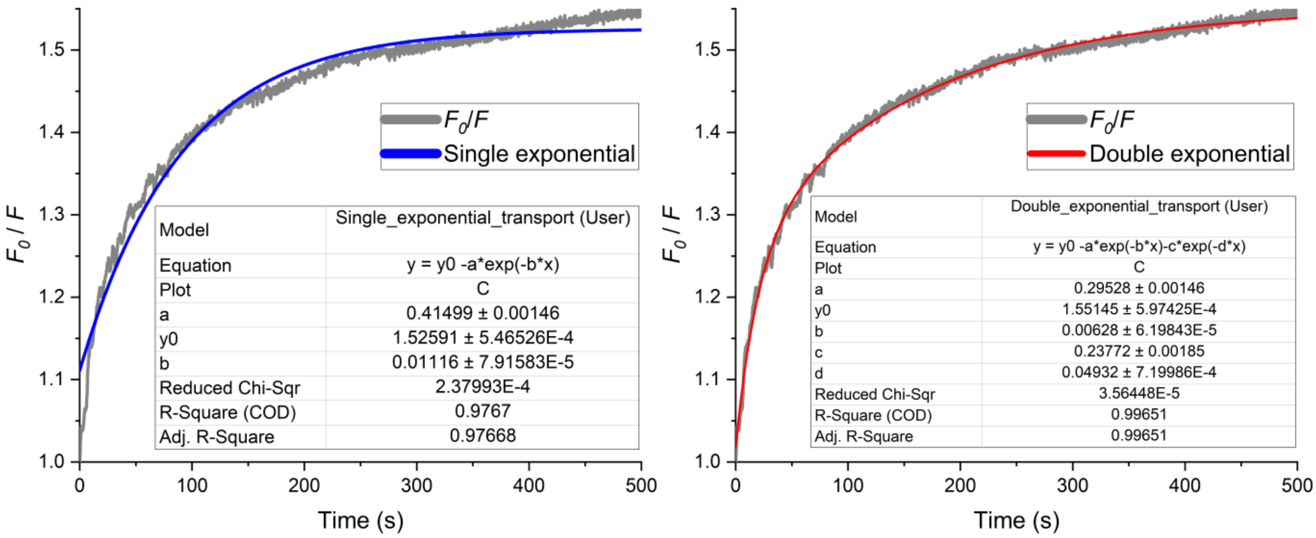

Figure S38: Fitting of transport data (Cl^-/HCO_3^- antiport) plotted as F_0/F obtained for transport of **3** in LUVs made of pure POPC by a) single exponential and b) double exponential equation.

4.2. Results obtained from the lucigenin assay

a) b)

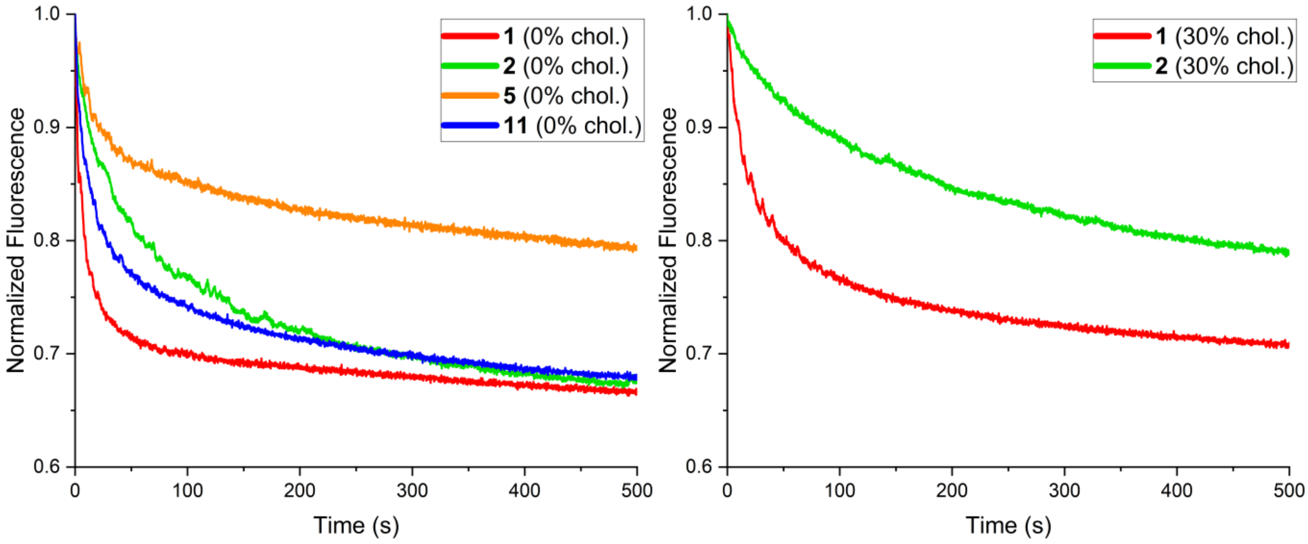

Figure S39: Cl⁻/HCO_3^- antiport by BU **1**, **2**, **5** and **11** (at 1:50k) measured by the lucigenin assay. LUVs are suspended in 225 mM $NaHCO_3$ and the transport experiment is initiated with a 25 mM NaCl pulse.

Table S4: The properties of the BU anionophores and the obtained transport rate constants and initial transport rates for Cl⁻/HCO_3^- antiport with BUs preincorporated at a 1:50000 BU:lipid ratio, determined using the lucigenin assay (**Figure S39**).

BU	Subst. *para*	Subst. *meta*	M_w (g mol^{-1})	logP of single GU[a]	SlogP[b]	σ_m/σ_p	logK_a in MeCN Cl⁻ (M^{-1})	*k* (10^3 s^{-1}) (0% chol.)	*k* (10^3 s^{-1}) (30% chol.).	*I* (10^3 s^{-1}) (0% chol.)	*I* (10^3 s^{-1}) (30% chol.)
**1**	-H	-CF_3	3638	6.6	39	0.43	11.2[c]	25	12	28	10
**2**	-H	-SCF_3	4408	11.3	54	0.40	11.7[c]	8	4	7	2
**5**	-OCH_3	-SCF_3	4768	11.1	54	-	11.7[c]	7	-[d]	8	-[d]
**11**	-OSO_2CF_3	-H	3783	5.1	18	0.53	10.5	10	-[d]	15	-[d]

[a] – calculated in ChemDraw, (**Figure S43**)

[b] – calculated using TorchLite 10.0.0

[c] – value from Chvojka, 2025[2]

[d] – No transport observed

a) b) c)

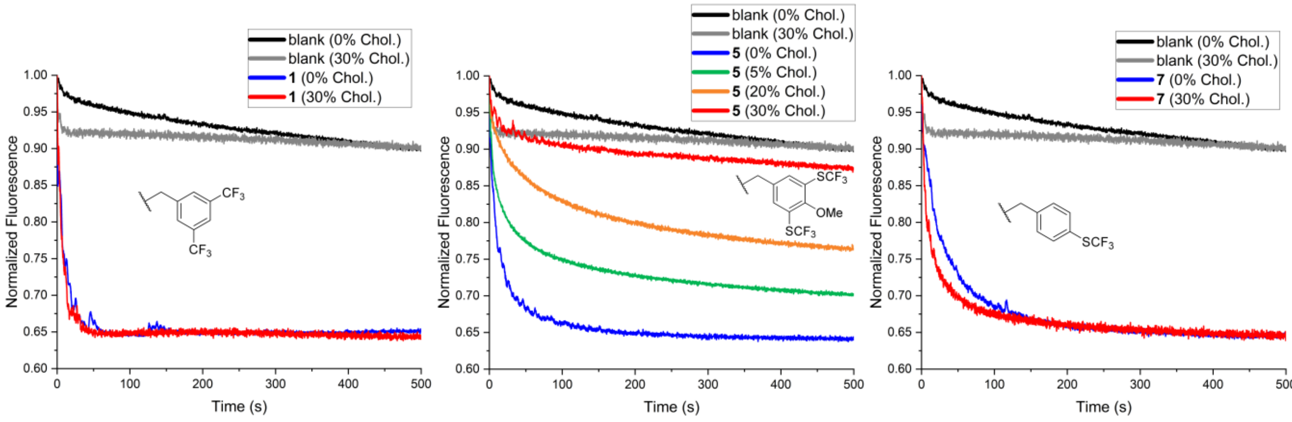

Figure S40: Examples of the effect of cholesterol presence in the membrane on transport properties of BUs: a) **1**; b) **5**; c) **7**. Cl⁻/HCO_3^- antiport monitored by the lucigenin assay. LUVs are suspended in 225 mM $NaHCO_3$ and the transport experiment is initiated with a 25 mM NaCl pulse.

4.3. Results obtained from the HPTS assay for 1

The ratios of fluorescence intensities were normalised to [0,1], as $(R\text{-}R_0)/(R_f\text{-}R_0)$, where R_t is the fluorescence ratio at time t, R_0 is the fluorescence ratio at t = 0, and R_f is the final fluorescence ratio after the addition of the detergent.

a) b)

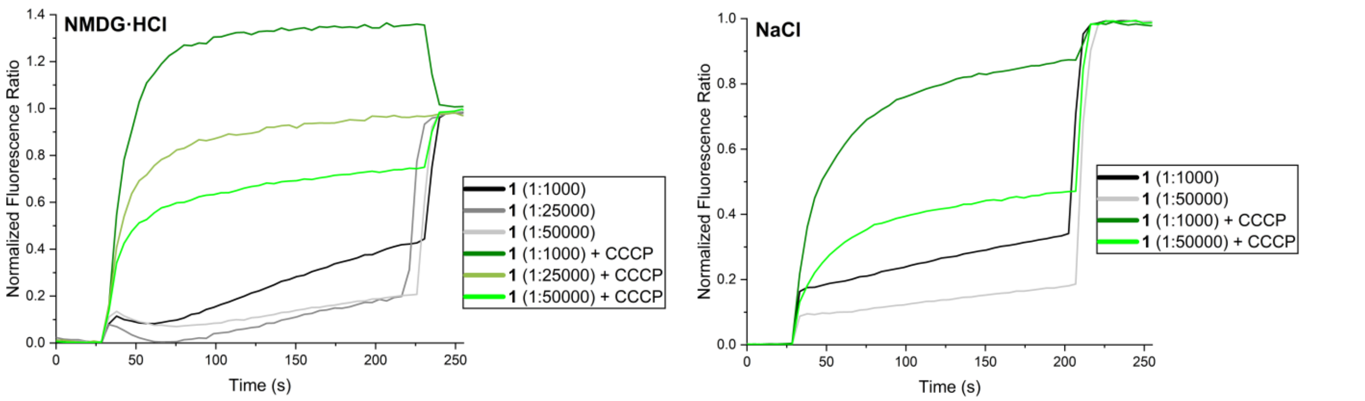

Figure S41: Cl^- uniport by **1** measured by the HPTS assay. LUVs are suspended at pH 6.8 in a) 100 mM NMDG·HCl and 10 mM HEPES or b) 100 mM NaCl and 10 mM HEPES. CCCP is post-inserted at a 1:1000 CCCP to lipid ratio. The transport experiment is initiated with a 5 mM NMDG or NaOH pulse.

a) b)

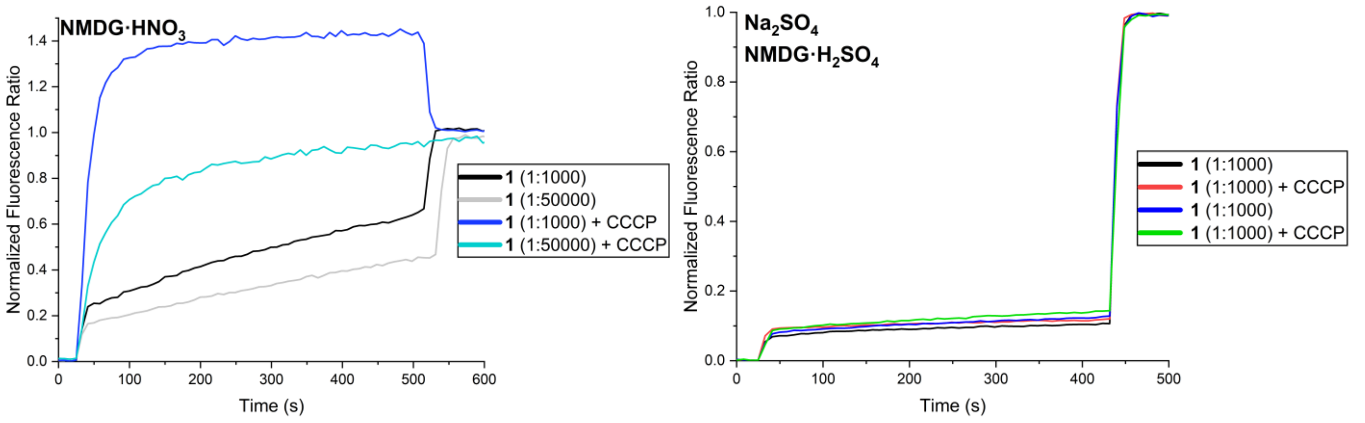

Figure S42: NO_3^- and SO_4^{2-} uniport by **1** measured by the HPTS assay. LUVs are suspended at pH 6.8 in a) 100 mM $NMDG{\cdot}HNO_3$ and 10 mM HEPES or b) 100 mM $NMDG{\cdot}H_2SO_4$ (blue and green curves) or 100 mM Na_2SO_4 (black and red curves) and 10 mM HEPES. CCCP is post-inserted at a 1:1000 CCCP to lipid ratio. The transport experiment is initiated with a 5 mM NMDG or NaOH pulse.

5. Computational studies

log P for a single GU building blocks (**Figure S43**) were calculated in ChemDraw Professional 19.1.1.21. Geometries of BU structures were optimized using semi-empirical method at PM6 level of theory in Spartan '18, Version 1.4.4. Electrostatic potential maps (**Figure S44**) are shown in range from −400 kJ mol^{-1} (red colour) to 400 kJ mol^{-1} (blue colour) at IsoVal 0.002 e au^{-3}. Optimized structures were exported as .pdb files, which were then used as input for TorchLite 10.0.0 (Cresset software) to obtain logP values.[7]

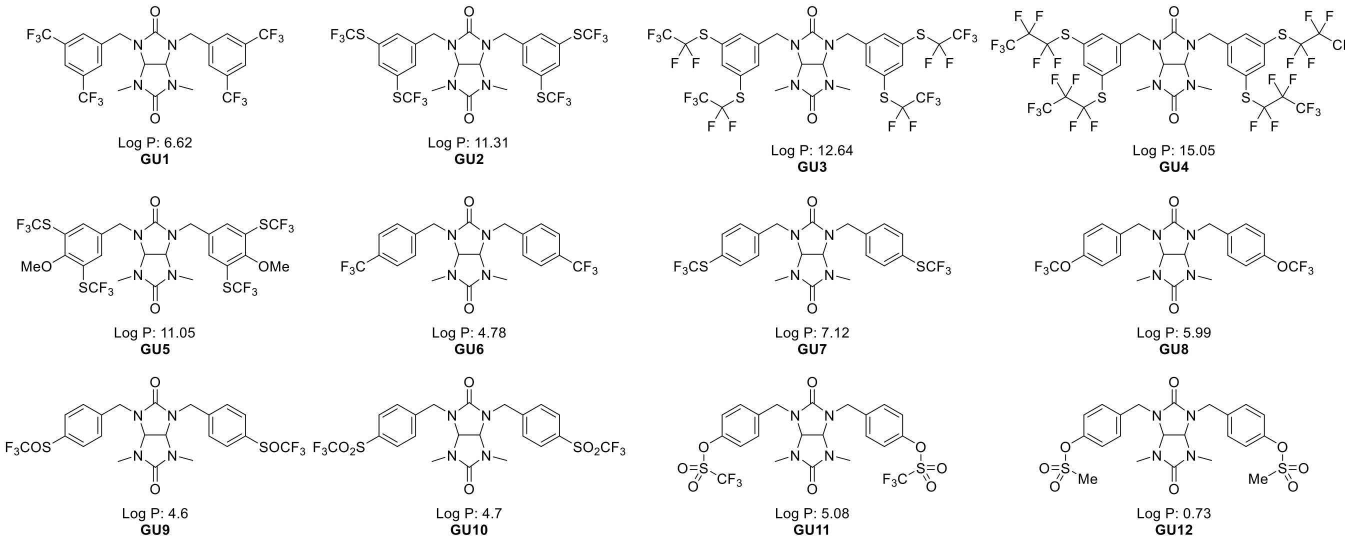

Figure S43: log P values calculated for GU building blocks using ChemDraw.

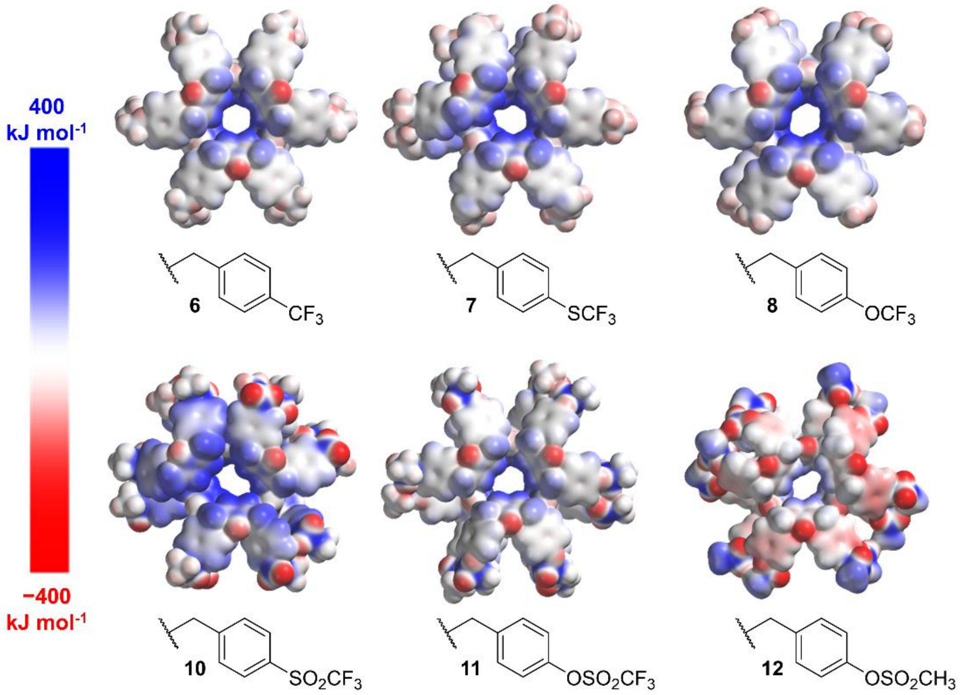

Figure S44:Calculated electrostatic potential maps of BU derivatives.

6. References

1 M. Chvojka, D. Madea, H. Valkenier and V. Šindelář, Tuning CH Hydrogen Bond-Based Receptors toward Picomolar Anion Affinity via the Inductive Effect of Distant Substituents, *Angew. Chem. Int. Ed.*, 2024, **63**, e202318261.

2 M. Chvojka, H. Valkenier and V. Šindelář, Synthesis of bambusurils with perfluoroalkylthiobenzyl groups as highly potent halide receptors, *Org. Chem. Front.*, 2025, **12**, 130–135.

3 G. R. Fulmer, A. J. M. Miller, N. H. Sherden, H. E. Gottlieb, A. Nudelman, B. M. Stoltz, J. E. Bercaw and K. I. Goldberg, NMR Chemical Shifts of Trace Impurities: Common Laboratory Solvents, Organics, and Gases in Deuterated Solvents Relevant to the Organometallic Chemist, *Organometallics*, 2010, **29**, 2176–2179.

4 H. Valkenier, O. Akrawi, P. Jurček, K. Sleziaková, T. Lízal, K. Bartik and V. Šindelář, Fluorinated Bambusurils as Highly Effective and Selective Transmembrane Cl−/HCO3− Antiporters, *Chem*, 2019, **5**, 429–444.

5 A. M. Gilchrist, P. Wang, I. Carreira-Barral, D. Alonso-Carrillo, X. Wu, R. Quesada and P. A. Gale, Supramolecular methods: the 8-hydroxypyrene-1,3,6-trisulfonic acid (HPTS) transport assay, *Supramol. Chem.*, 2021, **33**, 325–344.

6 M. Chvojka, A. Singh, A. Cataldo, A. Torres-Huerta, M. Konopka, V. Šindelář and H. Valkenier, The Lucigenin Assay: Measuring Anion Transport in Lipid Vesicles, *Anal. Sens.*, 2024, **4**, e202300044.

7 S. A. Wildman and G. M. Crippen, Prediction of Physicochemical Parameters by Atomic Contributions, *J. Chem. Inf. Comput. Sci.*, 1999, **39**, 868–873.
